# Taxonomic Revision of Neotropical *Downeshelea* Wirth and Grogan Predaceous Midges (Diptera: Ceratopogonidae)

**DOI:** 10.3390/insects11010009

**Published:** 2019-12-20

**Authors:** Maria Clara A. Santarém, Art Borkent, Maria Luiza Felippe-Bauer

**Affiliations:** 1 Coleção de Ceratopogonidae, Laboratório de Diptera, Instituto Oswaldo Cruz-Fiocruz, Av. Brasil 4365, Rio de Janeiro 21040–900, Brazil; mlfbauer@ioc.fiocruz.br; 2 Programa de Pós-Graduação em Biodiversidade e Saúde, Instituto Oswaldo Cruz-Fiocruz, Av. Brasil 4365, Rio de Janeiro 21040–900, Brazil; 3 Research Associate American Museum of Natural History, 691–8 th Ave. SE, Salmon Arm, BC V1 E 2 C2, Canada; artborkent@telus.net

**Keywords:** Ceratopogonini, new species, redescription, biting midges, aquatic

## Abstract

The genus *Downeshelea* was described by Wirth and Grogan based on the diagnostic characters of the *Monohelea multilineata* species group. The first descriptions of species were based on body coloration, which resulted in confusion and misunderstanding of their identification. The aim of this study was to provide an updated diagnosis and description of *Downeshelea*, describe 18 new species, and redescribe 10 previously poorly described species. New records of species, a key for identification of all New World species, and a table with important morphometric data to distinguish both males and females of the various species are provided along with distribution maps of the 46 known New World species.

## 1. Introduction

The Ceratopogonidae are a diverse family of Diptera with 6207 species [1] distributed worldwide, of which 1282 are known from the Neotropical Region [2]. They are quite numerous in almost all aquatic and semi-aquatic habitats but, due to their small size, have still been inadequately collected and studied [3].

As a result of the study of the New World species of *Monohelea s. lat.,* Kieffer by Wirth [4] and Wirth and Williams’ [5] study of the Nearctic species, Lane and Wirth [6] divided this genus into groups based on characters from male genitalia and coloration patterns of wings and legs. Lane and Wirth [6] recognized four distinct species groups: *fairchildi*, *hyeroglyphica*, *multilineata*, and *tesselata*. In 1988, Wirth and Grogan [7] placed the species of the *multilineata* group into a new genus *Downeshelea* based on several features of both males and females. The authors designated *Downeshelea stonei* (Wirth) as type species and maintained its position in Ceratopogonini.

The females of species of *Downeshelea* are predaceous midges that feed primarily on adult Chironomidae [8]. Because of their feeding habit, they may be important in the biological control of pests, as Yasumatsu [9] indicated took place in rice plantations in Thailand.

Currently, 42 species are included in *Downeshelea*, with 28 recorded from the Neotropical region (with two of these extending into the Nearctic) [1,10]. With the addition of 18 new species here, there are now 60 species known. The early descriptions were based, primarily, on authors’ interpretations of body coloration. This practice resulted in inconsistent species descriptions and misunderstandings in their identification. Several authors [4,6,11] observed that, in general, female specimens are indistinguishable, and the study of male genitalia is crucial for species separation. Furthermore, other researchers [12,13] demonstrated that characteristics of the head, wing, and legs of both sexes can be used to better characterize the species of this genus.

Considering the lack of studies of *Downeshelea* in the Neotropical region and the need to update the characterization of earlier described species, this work aims to redescribe and rediagnose the genus, provide descriptions of 18 new species and redescribe 10 previously known species. A key for identification and a table with important morphometric data of the males and females of species are provided, along with distribution maps of the 46 known Neotropical species.

## 2. Materials and Methods

The adult specimens studied are deposited in the following collections:
**Collection Acronym****Collection name**CCERColeção de Ceratopogonidae, Fundação Oswaldo Cruz, Rio de Janeiro, Brazil.CNCICanadian National Collection of Insects, Agriculture & Agri-Food Canada, Ottawa, Canada.FSPColeção Entomológica, Faculdade de Saúde Pública, USP, São Paulo, Brazil.INPAColeção de Invertebrados do Instituto Nacional de Pesquisa da Amazônia, Manaus, Amazonas, Brazil. LACMLos Angeles County Museum of Natural History, Los Angeles, California, United States.MLPAColección Entomológica de la División Entomología del Museo de La Plata, La Plata, Argentina.MNCRColección Entomológica, Sección de Artrópodos, Museo Nacional da Costa Rica.MNHNMuseo Nacional de Historia Natural, Cota Cota, Casilla, La Paz, Bolivia. MNRJColeção Entomológica, Museu Nacional do Rio de Janeiro, UFRJ, Rio de Janeiro, Brazil.MPEGColeção Entomológica, Museu Paraense Emilio Goeldi, Pará, Brazil.USNMEntomological Collection, Smithsonian Institution, National Museum of Natural History, Washington, United States.

Some specimens deposited in the FSP were pinned, which prevented further interpretation of some diagnostic characters. They could not be slide mounted, due to institutional policies of retaining the original preservation and the risk of damaging the ancient specimens fixed in celluloid strips during the mountage process.

Diagnostic characters were illustrated or photomicrographed using a Nikon Eclipse E 200 (Nikon, NY, USA) and a Zeiss AX10 microscope (Zeiss, Germany), except those from FSP that were taken with a cellphone attached to the microscope. Photomicrography and illustrations were prepared using Combine Z (www.combinezp.software.informer.com) and Photoshop GIMP Portable (www.gimp.org), respectively. The aedeagus and parameres of some species were drawn separately from the remainder of the male terminalia for better visualization. The distribution maps were created using QGis 2.18.19 (https://qgis.org) based on slide label coordinates or as approximate coordinates obtained in FallinGrain (www.fallingrain.com). The ISO Alpha03 code was used to identify the countries. Species distributions are presented on maps to better visualize their distribution, using an alphabetical arrangement, when possible. Morphological terms are from Borkent et al. [14]. The antennal ratio was obtained by dividing the combined lengths of the distal five flagellomeres by the combined lengths of the preceding eight flagellomeres for females and the distal four by the preceding nine flagellomeres for males, as was done in our earlier species description of *Downeshelea* [10,15,16,17,18,19,20,21]. The distribution of spines on each of tarsomeres 2–4 of all legs are separated by hyphens. Measurement of spermathecae are given in micrometers and those of the wings in millimeters. Meristic information is given as range, following by the mean and number of specimens examined. A table including the measurements important for identification of all Neotropical species is provided. Some females can be diagnosed and are distinctive while others can only be identified when associated with their respective males. The features described in the key are all figured when possible. When the feature is present in several species, we selected only one to represent it.

Neary all leg figures were those of males and, due to the similarity between both sexes, the figures from *Downeshelea lanei* Felippe-Bauer and Borkent and *D. stonei* were those of the female, which were in better condition. The specimens of *D. balboa* (Lane and Wirth), *D. blantoni* (Lane and Wirth) and *D. chiapasi* (Lane and Wirth) were pinned and only wing and leg coloration and male genitalia were redescribed; data from head, thorax, halter, and some female measurements were taken from their original descriptions. Due to the lack of data available for comparison in pinned specimens, the females from *D. balboa* and *D. blantoni* were not included in the key and diagnoses. The female redescription of *D. chiapasi* was based on additional material from Costa Rica. For species described or redescribed recently [15,16,17,18,19,20,21], we include only the diagnoses, distributions, updated measurements in the table, figures of wing, legs, and spermathecae and illustrations of male genitalia taken from their original publications. Characters to distinguish them from other *Downeshelea* are in the key.

## 3. Results and Discussion

### 3.1. Downeshelea genus Wirth and Grogan

*Downeshelea* Wirth and Grogan, 1988 [7]: 50. Type species: *Monohelea stonei* Wirth, by original designation.

**Diagnosis**. Male: the only Ceratopogonidae with the following combination of character states: wing with two distinctive black spots, one on r-m extending over the medial fork, the other in r_3_ from the apex of 2nd radial cell to M_1_ (Figure 3a); other grayish markings generally present. Claws paired, equal-sized; male sternite 9 with median convex lobe bearing several long setae (Figure 9j) and aedeagus a single piece, without sclerotized basal loop. Female: the only Ceratopogonidae with the following combination of character states: wing as described for male; fore-, mid legs claws paired, equal-sized (Figure 11g), with internal, external basal teeth and hind leg claw single (Figure 11g) without a basal tooth.

**Description.** Small to large sized specimens; wing length 0.75–2.00 mm. Eyes bare, contiguous in lower portion in females (Figure 11e), separate (Figure 3b) or contiguous (Figure 17c) in males. Antenna with distal 3 segments elongate in male (Figure 5b), distal 5 segments elongate in female (Figure 31c); male antenna with well-developed plume (Figure 5b), segments not fused. Third palpal segment with round sensory pit variable in size and depth (Figure 3f). Female mandible with 7–15 well-developed teeth. Thorax moderately broad, convex; small humeral depressions pale; scutellum brown mesally. Wing hyaline, covered with fine microtrichia, with scattered macrotrichia on the margin of radius cell and at wing margin (Figure 23a), dark bristles on costa (Figure 23a), also in radius in females (Figure 32d); two distinctive black spots, one on r-m extending over medial fork (Figure 3a), other in r_3_ from the apex of 2nd radial cell to M_1_ (in *D. unimaculata* (Debeham) it is a faint shaded area) (Figure 3a); other grayish, irregular markings present (except in *D. venus* sp. nov.); media petiolate, forking just beyond r-m crossvein. Legs slender, unarmed; hind leg slightly stouter (Figure 5c); femorotibial joint areas yellowish (except in *D. nigra* (Tokunaga)) (Figure 5c); legs brown (Figure 3c), pale brown (Figure 42b) or yellowish with dark bands (Figure 15d), some specimens with color gradient; fore-, hind tibia with apical spur, longer on foreleg; hind tibial comb with 6–8 spines (except in *D. whartoni* (Ratanaworabhan and Wirth) with 4 spines). Tarsi pale with scattered setae; fore-, midtarsomere 4 cylindrical (Figure 8f), hind tarsomere 4 greatly elongate (Figure 8f); fore-, midtarsomere 5 basoventrally swollen (Figure 12g); foretarsomere 1 with basal, apical spines (except in *D. venus* sp. nov.), tarsomeres 2–4 with apical spines, basal spines absent; midtarsomere 1 with two apical, two basal spines (*D. bimaculata* (Clastrier and Delécolle) with 1 basal), other ventral spines generally present; hind tarsomere 1 with one basal (Figure 26b), one apical spine; ventral palisade setae in a single row on hind tarsomere 1 (Figure 26b); females with hind tarsomere 4 with two apical spines (Figure 7g); Neotropical species with one apical spine in hind tarsomeres 2–3 (except in *D. magna* sp. nov.). Male claws paired, smaller, equal-sized. Female claws paired, long, curved, equal-sized, with internal and external basal teeth on fore-, mid legs (Figure 11g); hind leg claw single, elongate, without basal tooth (Figure 11g). Male genitalia with tergite 9 gradually tapering distally, distal ½ usually with sclerotized band laterally (Figure 6e), apicolateral process with apical setae; sternite 9 with a median convex lobe bearing several long setae (Figure 9j); gonostylus with basal ½ pilose, apical setae, sometimes with median setae, often tapering to pointed tip; parameres separate (Figure 9c) or fused (Figure 6b) of various shapes, with basal arch heavily sclerotized; median (Figure 9f), subapical processes (Figure 19g) or distal portion (Figure 6b) bent ventrally, when present; aedeagus undivided, without sclerotized basal loop. Female genitalia with median sclerite between tips of sternite 9, forming a triangular genital sclerite (Figure 3g); two ovoid spermathecae variable in size, with short sclerotized necks (Figure 4g); 3rd rudimentary spermatheca present (except in *D. venus* sp. nov.) (Figure 4g).

**Distribution and bionomics**. Most species of this genus are Neotropical, where 46 species are recorded. A further 14 species are known from the Afrotropical, Australasian, Nearctic, and Oriental Regions [22]. Adults female are predaceous midges and species are often found associated with coastal and/or humid forested areas. The immatures are unknown [23] but, as member of the subfamily Ceratopogoninae, the larvae are likely to be aquatic (as are all other known species in this group).

**Remarks**. Our study reveals that females are generally indistinguishable, corroborating the observations of Wirth [4], Lane and Wirth [6], and Ratanaworabham and Wirth [11]. However, the species that have yellowish brown legs with dark bands can be distinguished more easily. Considering this, for a confident identification of females, it is often crucial to have associated males, and this is particularly true for those species with uniform leg coloration. *Downeshelea* was included in the *Monohelea* complex by Wirth and Grogan [7] with *Allohelea* Kieffer, *Austrohelea* Wirth and Grogan, *Isthmohelea* Ingram and Macfie, *Monohelea* Kieffer and *Schizohelea* Kieffer, considering they were all originally considered to be *Monohelea*. *Austrohelea*, *Isthmohelea*, and *Schizohelea* can be readily distinguished from the other three genera by the unpatterned wing. Species of *Monohelea* and *Downeshelea* are generally more similar, differing from *Allohelea* primarily by the aspect of legs and claws and aedeagus without proximal and distal pieces. Adult *Downeshelea* can be easily separated from *Monohelea* by the pattern of wing spots and the shape of the aedeagus. Although Wirth and Grogan [7] cited the equal-sized spermathecae as a difference between these genera, we observed that females from *Downeshelea* have spermathecae variable in size. Other differences observed in relation to the original description of *Downeshelea* [7] were already cited by Santarém et al. [10] for the multilineata species group.

### 3.2. Identification Key to Male and Female *Downeshelea* from the Neotropical Region

1 r_3_ with apical grayish spot (Figure 13a) ……………………………………………………………….2

1′ r_3_ without apical grayish spot (Figure 3a) …………………………………………………………**16**

2 Wing length 1.63–2.00 mm; wing with white spot in cua_1,_ not extending to wing margin (Figure 31a); female with 2-2-3 apical spines on hind tarsomeres 2–4 …………………… ***D. magna* sp. nov.**

2′ Wing length 0.85–1.57 mm; wing with white spot in cua_1_ extending to wing margin (Figure 41a); female with 1-1-2 apical spines on hind tarsomeres 2–4 ………………………………………………**3**

3 Hind tibial comb with eight spines; 3rd rudimentary spermatheca long (18 µm) (Figure 13g); parameres separated, stem straight, distal portion mesally curved (Figure 14l) ……………………

…………………………………………………………………… ***D. charrua*** **Felippe-Bauer and Spinelli**.

3′ Hind tibial comb with 6–7 spines; 3rd rudimentary spermatheca short to moderately long (5–15 µm) (Figure 30f); parameres stem and distal portion not as above ……………………………………**4**

4 CuA_1_ pale (Figure 16a); hind femur with distinct subapical dark band, hind tibia with distinct dark bands subbasally, apically (Figure 16c) ……………………………………………………………….**5**

4′ CuA_1_ grayish (Figure 30e); hind femur without distinct subapical dark band, hind tibia without distinct dark bands subbasally, apically (Figure 27c) …………………………………………………….**6**

5 Yellowish species; female hind leg claw 1.06–1.30 length of 5th tarsomere (Figure 16h; Table 1); spermathecae pale (Figure 16g); male terminalia yellowish; gonocoxite brown, darkening apically (Figure 16d); gonostylus light brown at basal ½, distal ½ dark brown (Figure 16d) ………………………………………………………………………..…. ***D. chirusi* (Lane and Wirth).**

5′ Brown species; female hind leg claw 1.33–1.44 length of 5th tarsomere (Figure 34g; Table 1); spermathecae dark (Figure 34h); male terminalia brown; gonocoxite uniformly dark brown (Figure 34h), gonostylus uniformly dark brown (Figure 34d) ……………………….……***D. pulla* sp. nov.**

6 Hind femur darker subapically (Figure 41c) ……………………………………………….………….**7**

6′ Hind femur not darker subapically (Figure 23c) ……………………………………………………**11**

7 Distal portion of each paramere short, bent ventrally (Figure 43g) ……. ***D. tripunctata* sp. nov.**

7′ Distal portion of each paramere long (Figure 14i) or bifid (Figure 14f), not bent ventrally ……….**8**

8 Legs pale brown, hind tibia darker subbasally (Figure 13c); paramere with distal process (Figure 14i); aedeagus rectangular (Figure 14h) …………………………………………………………………**9**

8′ Legs brown, hind tibia not darker subbasally (Figure 27c); paramere without distal process (Figure 28j); aedeagus triangular (Figure 14e) or subtriangular (Figure 28i) ……………………………………**10**

9 Fore-, midfemur darker subapically, hind tibia darker apically (Figure 30g); unequal spermathecae (Figure 30f); male distal portion of paramere medially directed (Figure 33d); aedeagus without admedian sclerotized areas (Figure 33c) ……………………. ***D. lanei* Felippe-Bauer and Borkent.**

9′ Fore-, midfemur not darker subapically, hind tibia not darker apically (Figure 13c); subequal spermathecae (Figure 13d); male distal portion of paramere posteriorly directed (Figure 14i); aedeagus with two admedian sclerotized areas (Figure 14h) …………***D. cebacoi* (Lane and Wirth).**

10 Female mandible with 11 teeth (Table 1); equal-sized spermathecae (Figure 27g); gonostylus moderately long (Figure 28i; Table 1); paramere stem sinuous, with short median process (Figure 28j), distal portion single (Figure 28j) ……………………………………………………… ***D. jurgeni* sp. nov.**

10′ Female mandible with 9 teeth (Table 1); unequal spermathecae (Figure 12f); gonostylus short (Figure 14d; Table 1); paramere stem nearly straight, without median process, distal portion bifid (Figure 14f) ……………………………………………………. ***D. castroi* (Tavares and Silva-Pereira).**

11 Gonocoxite slender (Figure 45a; Table 1); distal portion of paramere very long (Figure 45b) ……………………………………………………………………………………. ***D. wirthiana* sp. nov.**

11′ Gonocoxite stout or moderately stout (Figure 9g; Table 1); paramere without distal process (Figure 33f) or with a short one (Figure 33b) ……………………………………………………………………**12**

12 2nd radial cell 2.5 length of first (Figure 30a); male tergite 9 with short apicolateral processes (Figure 33a); distal portion of paramere curled up, forming a spiral process (Figure 33b) …………………………………………………………………………………………. ***D. kuna* sp. nov.**

12′ 2nd radial cell twice length of first; male tergite 9 with long apicolateral processes (Figure 37h); distal portion of paramere simple pointed (Figure 33f) or tapering to pointed tip, externally directed (Figure 24j) ………………………………………………………………………………………………..**13**

13 Equal or subequal spermathecae; paramere with median horn-like process (Figure 37i) ………**14**

13′ Unequal spermathecae; paramere with pointed (Figure 33f) or hook-like median process (Figure 19i) ……………………………………………………………………………………………………………**15**

14 Grayish spot in CuA_1_ extending into cua1 (Figure 23a,d); paramere with median process delicate, straight (Figure 24j), distal portion greatly bent externally … ***D. fuscipennis* (Lane and Wirth).**

14′ Grayish spot in CuA_1_ not extending into cua1 (Figure 36a,d); paramere with median process stout, curved (Figure 37i), distal portion bent externally to a short, sharp point … ***D. rodriguezi* sp. nov**.

15 CuA_1_ with subapical grayish spot (Figure 31f); 3rd rudimentary spermatheca moderately long (15 µm); male tergite 9 long (Figure 33e); paramere with clear, bulbous ventral lobe at midportion (Figure 33f), with pointed median process (Figure 33f), distal portion simple pointed (Figure 33f) ……………………………………………………………………………… ***D. oliveirai* Felippe-Bauer.**

15′ CuA_1_ with widely grayish spot (Figure 18a,d); 3rd rudimentary spermatheca short (8 µm); male tergite 9 short (Figure 19h); paramere without ventral lobe at midportion, with hook-like median process (Figure 19i), distal portion tapering to pointed tip externally directed (Figure 19i) …………………………………………………………………………………………. ***D. curta* sp. nov.**

16 Hind tibia with distinct subbasal, apical dark brown band (Figure 4c) ……………………….**17**

16′ Hind tibia without distinct subbasal, apical dark brown band (Figure 22c) ………………….**21**

17 CuA_2_ without grayish spot (Figure 4a,e); female hind leg claw 1.69 as long as 5th tarsomere (Figure 4h) …………………………………………………………………………………………. ***D. avizi* sp. nov.**

17′ CuA_2_ with grayish spot (Figure 17a,d); female hind leg claw 1.29–1.50 as long as 5th tarsomere (Figure 15e) ………………………………………………………………………………………………..**18**

18 Hind tibial comb with eight spines; equal-sized spermathecae (Figure 15f); parameres separated (Figure 19c); aedeagus distal portion with two non-serrate processes (Figure 19b) …………………………………………………………………………. ***D. chiapasi* (Lane and Wirth).**

18′ Hind tibial comb with 6–7 spines; spermathecae not equal in size; male parameres fused (Figure 19g); aedeagus distal portion with two serrate processes (Figure 28 a) …………………………….**19**

19 Hind femur with basal dark band (Figure 38c); male terminalia entirely pale brown (Figure 43a) ………………………………………………………………………………………… ***D. spatha* sp. nov.**

19′ Hind femur without basal dark band (Figure 25c); male terminalia brown or yellowish ……….**20**

20 Male terminalia entirely brown; parameres stem with subapical process deeply curved (Figure 19g) ………………………………………………………………………. ***D. colombiae* (Lane and Wirth).**

20′ Male terminalia yellowish, distal ½ of gonostylus brown (Figure 28a); parameres stem tapering apically, with sinuous apical process (Figure 28b) ………………………………. ***D. gladius* sp. nov.**

21 Wing with indistinct grayish areas (Figure 42a,d); aedeagus Y- shaped with large base, rectangular basally, triangular distally (Figure 43h) …………………………………………….. ***D. venus* sp. nov.**

21′ Wing with distinct grayish areas (Figure 3a,d); aedeagus not Y-shaped ……………………….**22**

22 Male paramere stem divergent from near base (Figure 9i) or for distal half (Figure 24e), apical portion as a bifid process (Figure 9i) ……………………………………………………………………**23**

22′ Male paramere stem straight (Figure 6h), sinuous (Figure 6d) or curved (Figure 9c); apical portion not as above ………………………………………………………………………………………….…….**25**

23 Hind tibia pale apically (Figure 35e); distal portion of paramere with outer process spirally (Figure 37g); aedeagus with distal sclerotized dorsal lobe (Figure 37f) ……………………***D. quechua* sp. nov.**

23′ Hind tibia not pale apically (Figure 7c); paramere not as above; aedeagus without sclerotized dorsal lobe (Figure 9h) ……………………………………………………………………………….……**24**

24 Fore-, midtibia distinctly lighter brown than hind tibia (Figure 7c); gonocoxite stout (Figure 9g; Table 1); aedeagus rectangular with two elliptical sclerotized area (Figure 9h) …***D. bifida* sp. nov.**

24′ Fore-, midtibia as brown as hind tibia (Figure 22c); gonocoxite slender (Figure 24d; Table 1); aedeagus subtriangular with ventral membrane projection (Figure 24d) …***D. divergentis* sp. nov.**

25 Parameres separate (Figure 24h) ……………………………………………………………………**26**

25′ Parameres fused (Figure 28h) …………………………………………………………………………**29**

26 Paramere with median process (Figure 24h); aedeagus triangular (Figure 24g) …………………**27**

26′ Paramere without median process (Figure 9c); aedeagus rectangular (Figure 9b) or quadrangular (Figure 37d) ……………………………………………………………………………………………….**28**

27 Paramere with beak-shaped processes (Figure 24h); aedeagus with two basal, anteriorly-directed, horn-like processes (Figure 24g) ……………………… ***D. fluminensis* Felippe-Bauer and Quintelas.**

27′ Paramere with greatly curved processes (Figure 14c); aedeagus without median horn-like processes (Figure 14b) …………………………………………. ***D. carioca* (Tavares and Silva-Pereira).**

28 Paramere stem sinuous, distal portion as membranous lobe (Figure 37e); aedeagus quadrangular with median horn-like processes (Figure 37d) …… ***D. quasidentica* Felippe-Bauer and Quintelas.**

28′Paramere stem externally curved, distal portion tapering (Figure 9c); aedeagus rectangular without median horn-like processes (Figure 9b) ………………………… ***D. balboa* (Lane and Wirth).**

29 Paramere without median, subapical or distal processes (Figure 28e,h) ……………………….**30**

29′ Paramere with median (Figure 9f), subapical (Figure 33h) or distal processes (Figure 9l) …**31**

30 Midtarsomere 1 with 1–4 ventral spines (Table 1); paramere apex pointed, posterolaterally directed (Figure 28e); aedeagus triangular (Figure 28d) …… ***D. grogani* Huerta, Felippe-Bauer and Spinelli.**

30′ Midtarsomere 1 without ventral spines (Table 1); paramere apex foot-shaped, externally directed (Figure 28h); aedeagus subrectangular (Figure 28g) ………………………………***D. guianae* (Wirth).**

31 Paramere subapical process hyaline, slender basally, expanded on midportion, abruptly tapering to a long pointed tip (Figure 6h); aedeagus subtriangular, expanded mesally, tapering distally to two sclerotized pointed processes (Figure 6g) …………………………………………***D. bahiana* sp. nov.**

31′ Paramere and aedeagus not as above ………………………………………………………………**32**

32 Female flagellomeres brown (Figure 32e); paramere with subapical process straight, directed anteriorly (Figure 33h) ……………………………………………… ***D. panamensis* (Lane and Wirth).**

32′ Female flagellomeres 2–8 or 1–7 pale basally (Figure 8e); paramere without subapical process (Figure 9l) …………………………………………………………………………………………………**33**

33 Third palpal segment with pit small (Figure 20b); paramere with medial process (Figure 24c) ……………………………………………………………………………………………………………**34**

33′ Third palpal segment with pit broad (Figure 3f); paramere with apical process (Figure 6d) …**35**

34 Paramere apex and median horn processes equal in size and shape (Figure 9f) ……………………………………………………………. ***D. bicornis* Felippe-Bauer and Quintelas.**

34′ Paramere apex more delicate and sharp than the median horn processes, the right longer than the left one (Figure 24c) ………………………………………. ***D. deanei* Felippe-Bauer and Quintelas.**

35 Female flagellomeres brown, 2–8 paler basally (Figure 3e); distal portion of paramere bent on two directions (Figure 43e) …………………………………………………………………………………….**36**

35′ Female flagellomeres brown, 1–7 paler basally; distal portion of paramere bent on one direction, tapering to pointed tip (Figure 6b) ***multilineata* species group** (Figures in Santarém et. al. [10], except *D. blantoni*) ………………………………………………………………………………………….**38**

36 Paramere stem slightly enlarged subapically (Figure 43e); aedeagus rectangular (Figure 43d) ………………………………………………………………………………………… ***D. stonei* (Wirth).**

36′ Paramere stem enlarged (Figure 6d) or tapering apically (Figure 9l); aedeagus subtriangular (Figure 9k) ……………………………………………………………………………………………….**37**

37 Hind tibia slightly darker subbasally (Figure 8c); gonostylus nearly straight (Figure 9j); paramere stem tapering apically (Figure 9l); posterodistal portion internally directed, forming horn-like process (Figure 9l) ……………………………………………………………………………… ***D. capra* sp. nov.**

37′ Hind tibia not darker subbasally (Figure 3c); gonostylus swollen on basal ½ (Figure 6c); paramere expanded laterodistally (Figure 6d); posterodistal portion externally directed, not forming horn-like process (Figure 6d) …………………………………………………………………….. ***D. alia* sp. nov.**

38 Hind femur uniformly brown …………………………………………………………………………**39**

38′ Hind femur darker subapically ……………………………………………………………………**41**

39 Aedeagus rectangular (Figure 6a); gonocoxite stout (Table 1); parameres without a posterior projection on the medially fused portion (Figure 6b) …………………. ***D. blantoni* (Lane and Wirth).**

39′ Aedeagus subtriangular; gonocoxite slender (Table 1); parameres with a posterior projection on the medially fused portion ……………………………………………………………………………….**40**

40 Tergite 9 with short, broad apicolateral process; paramere stem sinuous on midportion, expanded subapically forming a broad lobe laterally directed; aedeagus without elliptical sclerotized anterior areas ………………………………… ***D. eclectica* Santarém, Borkent, Spinelli and Felippe-Bauer.**

40′ Tergite 9 with long, slender apicolateral process; paramere stem sinuous, basal 2/3 directed posterolaterally, distal 1/3 slender directed posteromesally; aedeagus with two elliptical sclerotized anterior areas …………………………. ***D. moravia* Santarém, Borkent, Spinelli and Felippe-Bauer.**

41 CuA_1_ pale; distal portion of paramere short, not reaching the medially fused portion ………**42**

41′ CuA_1_ grayish; distal portion of paramere long, reaching the medially fused portion ………**44**

42 Legs without contrasting darker areas; gonostylus long (Table 1); paramere stem straight, gradually swollen to apex; aedeagus basal arch deep, V-shaped ……………………………………… ……………………………………… ***D. casimirensis* Santarém, Borkent, Spinelli and Felippe-Bauer.**

42′ Legs with contrasting darker areas; gonostylus moderately long; paramere stem not as above; aedeagus basal arch shallow, not V-shaped …………………………………………………………**43**

43 Legs pale brown, fore-, hind tibia darker apically; paramere stem basolaterally expanded, distal portion nearly straight; aedeagus subtriangular, without sclerotized anteromesal areas ………….

*……………………………………………….. **D. jarina*** Santarém, Borkent, Spinelli and Felippe-Bauer.

43′ Legs brown, fore-, hind tibia not darker apically; paramere stem not expanded basolaterally, distal portion strongly curved; aedeagus rectangular, with two large elliptical sclerotized anteromesal areas reaching midlength ……………. ***D. marambaia* Santarém, Borkent, Spinelli and Felippe-Bauer.**

44 Midtibia not paler on basal third; paramere stem expanded distally in inner portion, distal portion abruptly curved to tip …………………. ***D. litorale* Santarém, Borkent, Spinelli and Felippe-Bauer.**

44′ Midtibia paler on basal third; paramere stem straight distally or enlarged in outer portion, distal portion slightly or not curved to tip …………………………………………………………………….**45**

45 Foretibia brown; paramere stem straight; aedeagus with two elliptical sclerotized anterior areas ………………………………….……………………………………………… ***D. multilineata* (Lutz).**

45′ Foretibia paler basally; paramere stem sinuous, enlarged apically; aedeagus without sclerotized anterior areas ……………………. ***D. costaricensis* Santarém, Borkent, Spinelli and Felippe-Bauer**

### 3.3. Downeshelea multilineata *group*

*Downeshelea multilineata* group Santarém et al., 2018 [10]: 510.

**Diagnosis.** Male: only species group of *Downeshelea* in the Americas with the following combination of character states: wing with three grayish areas: first near apex of M_1_, second sigmoid-shaped extending from m_1_ to wing margin in m_2_, third over CuA_2_, (also in CuA_1_ in some species); legs brown or pale brown, without bands; apical spines of hind tarsomeres 2–4: 1-1-1; distal portion of paramere abruptly bent ventrally, directed anteromesally, tapering to pointed tip (Figure 6b), overlapping in some species. Female: only species group of *Downeshelea* in the Americas with the following combination of character states: wing and legs as described for male; antenna brown, basal portion of flagellomeres 1–7 pale.

A detailed study of this species group is available in Santarém et al. (2018) [10]. It did not, however, include *D. blantoni*.

#### 3.3.1. *Downeshelea blantoni* (Lane and Wirth)

**Figure 1:**
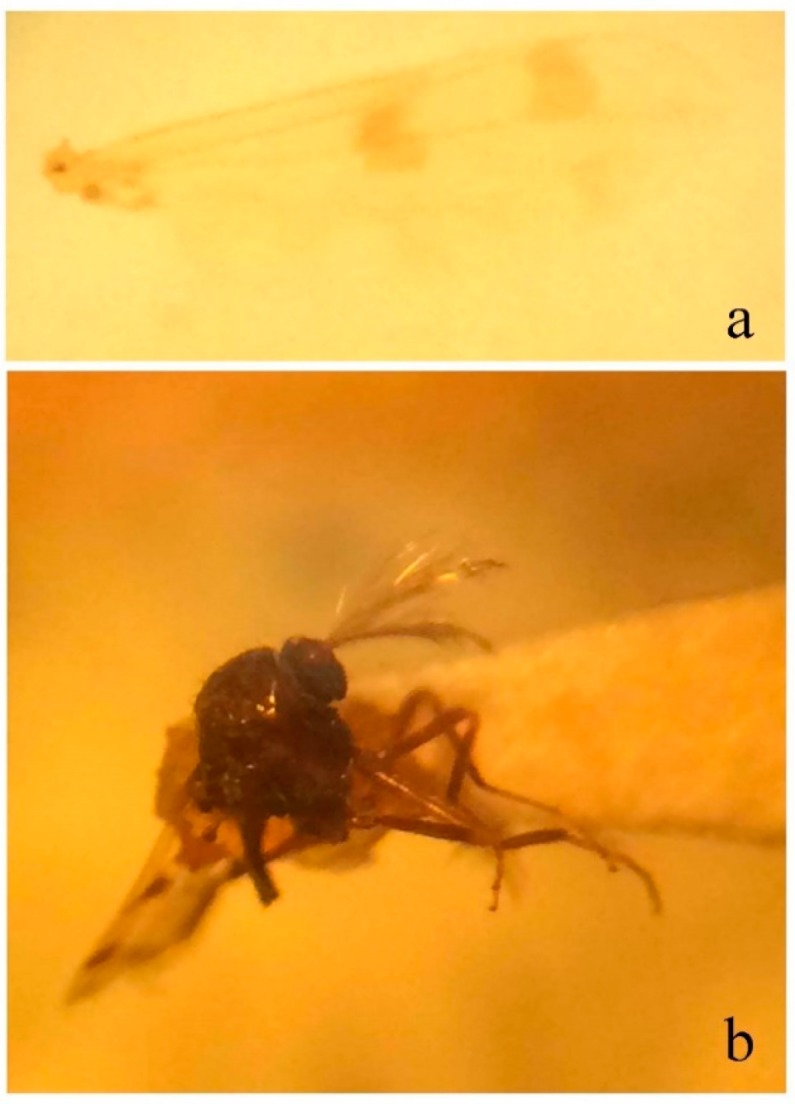
*Downeshelea blantoni*: (**a**) wing; (**b**) paratype male pinned specimen.

**Figure 2:**
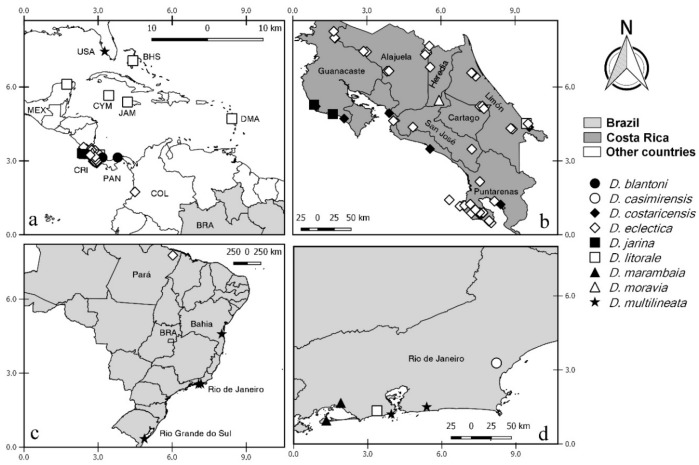
Geographic distribution of species in the *Downeshelea multilineata* species group: *D. blantoni* (**a**); *D. casimirensis* (**d**); *D. costaricensis* (**a**,**b**); *D. eclectica* (**a**–**c**); *D. jarina* (**a**,**b**); *D. litorale* (**a**,**b**,**d**); *D. marambaia* (**d**); *D. moravia* (**b**); D. multilineata (**c**,**d**).

Figure 1a,b, Figure 2, and Figure 6a,b; Table 1.

*Monohelea blantoni* Lane and Wirth, 1964 [6]: 217 (male, female; Panama); Wirth, 1974 [3]: 40 (in catalog south of the USA; distribution).

*Downeshelea blantoni*: Wirth and Grogan, 1988 [7]: 51 (combination); Borkent and Wirth, 1997 [24]: 97 (in World catalog); Borkent and Spinelli, 2000 [25]: 47 (in catalog south of the USA; distribution); Borkent and Spinelli, 2007 [26]: 80 (in Neotropical catalog; distribution); Borkent, 2016 [22]: 124 (in World catalog).

**Diagnosis**. Male: only species of *Downeshelea multilineata* group in the Americas with the following combination of character states: parameres fused on basal portion for 0.33 of total length, stem sinuous, basal 2/3 stout, directed posterolaterally, distal 1/3 slender, directed posteromesally (Figure 6b); distal portion sharp, 0.56–0.61 of total length (Figure 6b); aedeagus rectangular, basal arch U-shaped (Figure 6a), with two anterior sclerotized areas (Figure 6a). Female not diagnosable.

**Redescription. Male** (Figure 1b). Wing (Figure 1a) with three grayish areas: first near apex of M_1_, not reaching wing margin; second sigmoid-shaped, extending from m_1_ to wing margin in m_2_; third over CuA_1_, CuA_2_ extending into cua_1,_ anal cell, reaching wing margin; 2nd radial cell twice length of 1st; length 1.08 (n = 1) mm; width 0.37 (n = 1) mm; costal ratio 0.70 (n = 1). Halter knob infuscated. Legs brown, hind leg darker. **Abdomen.** Brown. Terminalia (Figure 6a): tergite 9 not visible in slide mounted specimens, sternite 9 with anterior margin not visible in slide mounted specimens, posterior margin with large convex median lobe bearing 2 long setae. Gonocoxite brown 1.90–2.00 (1.95, n = 2) times longer than basal width; gonostylus brown, nearly straight, truncated tip, 0.63–0.64 (0.63, n = 2) length of gonocoxite. Parameres (Figure 6b) 1.06–1.13 (1.10, n = 2) length of aedeagus, fused on basal portion for 0.33 (n = 1) of total length, each with basal arm trilobed, knob slender; stem sinuous, basal 2/3 stout, directed posterolaterally, distal 1/3 slender, directed posteromesally; distal portion (Figure 6b) sharp, anteriorly directed, 0.56–0.61 (0.58, n = 2) of total length. Aedeagus (Figure 6a) rectangular, slightly sclerotized laterally, basal arch U-shaped, with two anterior sclerotized areas (Figure 6a), extending to 0.29 (n = 2) of total length; distal portion with deep mesal notch and in two pointed processes.

**Female.** Similar to male with usual sexual differences. Wing length 1.11–1.20 (1.15, n = 2) mm; width 0.45 (n = 1); costal ratio 0.81 (n = 1).

**Specimens examined.** 1 male, 1 female, pinned, with wing and male genitalia in drop of Canada Balsam on celluloid strip on pin, labeled “Paratype, *Monohelea blantoni* Lane and Wirth, described”, “Panama, Mojinga Swamp, Ft Sherman, Canal Zone, 24 October 1951, light trap, FS Blanton col.” (FSP); 2 males, pinned, with male genitalia in drop of Canada Balsam on celluloid strip on pin, labeled “Paratype, *Monohelea blantoni* Lane and Wirth” same data (FSP); 1 male, pinned, with male genitalia in drop of Canada Balsam on celluloid strip on pin, labeled “Paratype, *Monohelea blantoni* Lane and Wirth” same data except “8 November 1951” (FSP); 4 females, pinned, labeled “Paratype, *Monohelea blantoni* Lane and Wirth” same data except “July 1952” (FSP); 1 male, pinned, with male genitalia in drop of Canada Balsam on celluloid strip on pin, labeled “Paratype, *Monohelea blantoni* Lane and Wirth”, same data except “Loma Boracho, 23 October 1951” (FSP).

**Distribution and bionomics.** This species is known only from coastal areas in Panama occurring at sea level (Figure 2).

**Taxonomic discussion.** This species is included in the *multilineata* species group because of its wing and leg pigmentation patterns and having a paramere with its distal portion long and abruptly bent ventrally, directed anteromesally. It most closely resembles *D. moravia* in leg pigmentation pattern and general aspect of parameres. *Downeshelea blantoni* can be distinguished from *D. moravia* by its smaller size (1.37 mm in *D. moravia*) (Table 1), the aedeagus rectangular without elliptical sclerotized anterior areas and distal portion non-serrate (subtriangular with elliptical sclerotized anterior areas, distal portion serrate in *D. moravia*) and sternite 9 with a large convex median lobe (poorly developed in *D. moravia*). Specimens of *Downeshelea blantoni* are only recorded from coastal areas in Panama at lower altitudes while *D. moravia* is restricted to higher altitudes (1600 m) in forested areas of Costa Rica [10] (Figure 2). Unfortunately, only one female wing was available for study from the paratypes listed herein. We therefore cannot make further comparisons with females of other species. The holotype and allotype of *D. blantoni* are pinned specimens (USNM #66436) and no further observations were made of them.

#### 3.3.2. *Downeshelea casimirensis* Santarém, Borkent, Spinelli, and Felippe-Bauer

*Downeshelea casimirensis* Santarém et al., 2018 [10]: 517 (male, Brazil); Santarém and Felippe-Bauer, 2019 [2]: 16 (Brazilian distribution).

**Distribution and bionomics.** This species is known only from forested area in Brazil (Rio de Janeiro) at 45 m above sea level (Figure 2).

#### 3.3.3. *Downeshelea costaricensis* Santarém, Borkent, Spinelli, and Felippe-Bauer

*Downeshelea costaricensis* Santarém et al., 2018 [10]: 518 (male, female, Costa Rica).

**Distribution.** This species is known only from coastal and mangrove areas in Costa Rica (Guanacaste, Puntarenas and Limón) from 5 to 100 m above sea level (Figure 2).

#### 3.3.4. *Downeshelea eclectica* Santarém, Borkent, Spinelli, and Felippe-Bauer

*Downeshelea eclectica* Santarém et al., 2018 [10]: 521 (male, female, Costa Rica, Colombia and Brazil); Santarém and Felippe-Bauer, 2019 [2]: 16 (Brazilian distribution).

**Distribution.** This species is known from forested, coastal, and mangrove areas in Costa Rica (Alajuela, Heredia, Puntarenas, San José, Cartago, and Limón), Colombia and Brazil (Pará) (Figure 2). It occurs in Costa Rica up to 1850 m above sea level. In other countries it has been found up to 15 m above sea level.

#### 3.3.5. *Downeshelea jarina* Santarém, Borkent, Spinelli, and Felippe-Bauer

*Downeshelea jarina* Santarém et al., 2018 [10]: 526 (male, female, Costa Rica).

**Distribution.** This species is known only from coastal and mangrove areas in Costa Rica (Guanacaste and Puntarenas), occurring 5 m above sea level (Figure 2).

#### 3.3.6. *Downeshelea litorale* Santarém, Borkent, Spinelli, and Felippe-Bauer

*Downeshelea multilineata* Huerta et al., 1999 [27]: 494 (misidentification; Mexico record).

*Downeshelea litorale*: Santarém et al., 2018 [10]: 533 (male, female, Mexico, Bahamas, Grand Cayman, Jamaica, Dominica, Costa Rica and Brazil); Santarém and Felippe-Bauer, 2019 [2]: 16 (Brazilian distribution).

**Distribution**. This species is known from Mexico (Yucatan), Bahamas, Grand Cayman, Jamaica, Dominica, Costa Rica (Limón) and Brazil (Rio de Janeiro) (Figure 2). It has been found in forests near coastal areas, occurring up to 35 m above sea level.

#### 3.3.7. *Downeshelea marambaia* Santarém, Borkent, Spinelli, and Felippe-Bauer

*Downeshelea marambaia* Santarém et al., 2018 [10]: 535 (male, Brazil); Santarém and Felippe-Bauer, 2019 [2]: 16 (Brazilian distribution).

**Distribution.** This species is known only from coastal areas in Brazil (Rio de Janeiro) occurring at sea level (Figure 2).

#### 3.3.8. *Downeshelea moravia* Santarém, Borkent, Spinelli, and Felippe-Bauer

*Downeshelea moravia* Santarém et al., 2018 [10]: 536 (male, Costa Rica).

**Description. Female**. Similar to male described by Santarém et al. [10]. Antenna damaged in slide mounted specimen; palpal ratio 2.0; mandible with 11 teeth. Wing as in male; length 1.57 mm; width 0.62 mm; costal ratio 0.79. Legs as in male. Foretarsomere 1 with one basal, one apical spine; midtarsomere 1 without ventral spines; apical spines of tarsomeres 2–4 of fore-, mid legs: 2-2-4, 2-2-4; foretarsal ratio 2.46, midtarsal ratio 2.42, hind tarsal ratio 2.62; fore-, midleg claws 0.64 length of their respective tarsomeres 5; hind leg claw 1.29 as long as tarsomere 5. Two equal-sized rounded spermathecae, 75 by 73 µm. Third rudimentary spermatheca 10 µm.

**Specimen examined.** Female, on microscope slide “Costa Rica, Prov. San José, Moravia, Zurquí de Moravia, Tower path, 1600 m (84°0′57″ W; 10°02′58″ N), 26 July–2 August 2013, malaise, 15 m, Proyecto ZADBI col.” (MNCR).

**Distribution.** This species is known only from forested areas in Costa Rica (San José) at 1600 m above sea level (Figure 2).

#### 3.3.9. *Downeshelea multilineata* (Lutz)

*Palpomyia multilineata* Lutz, 1914 [28]: 93 (male; Rio de Janeiro, Brazil).

*Monohelea multilineata*: Macfie, 1940 [29]: 137 (Guyana record; misidentification); Johannsen, 1943 [30]: 781 (combination); Lane, 1945 [31]: 368 (redescription; in part male specimen); Wirth, 1953 [4]: 149 (notes; in key; misidentification); Lane and Wirth, 1964 [6]: 224 (distribution; in key; misidentification); Wirth, 1974 [3]: 41 (in catalog south of the USA; distribution, Guyana record).

*Monohelea* (*Allohelea*) *multilineata*: Wilkening et al., 1985 [32]: 524 (Florida records).

*Downeshelea multilineata*: Wirth and Grogan, 1988 [7]: 52 (combination); Borkent and Wirth, 1997 [24]: 98 (in World catalog); Borkent and Spinelli, 2000 [25]: 47 (in catalog south of the USA; distribution); Borkent and Spinelli, 2007 [26]: 80 (in Neotropical catalog; distribution); Borkent and Grogan, 2009 [33]: 20 (in Nearctic catalog; distribution); Grogan et al., 2010 [34]: 35 (in Florida species list; distribution); Borkent, 2016 [22]: 124 (in World catalog); Santarém and Felippe-Bauer, 2019 [2]: 17 (Brazilian distribution); Santarém et al., 2018 [10]: 513 (redescription).

**New record**. 1 male, on microscope slide, labeled “Brazil, Rio Grande do Sul, Rio Grande, Ilha da pólvora, −32.033611 S −52.179167 W, 24 November 2011, D. Carrasco col.” (CCER); 2 females, in glycerol, same data (CCER).

**Distribution.** This species is known from USA (Florida), Guyana (?) and Brazil (Bahia, Rio de Janeiro, Rio Grande do Sul) (Figure 2). It has been found in coastal and forested areas and in cocoa plantations, occurring up to 150 m above sea level.

### 3.4. Other Neotropical *Downeshelea* species

#### 3.4.1. *Downeshelea alia* sp. nov.

**Figure 3:**
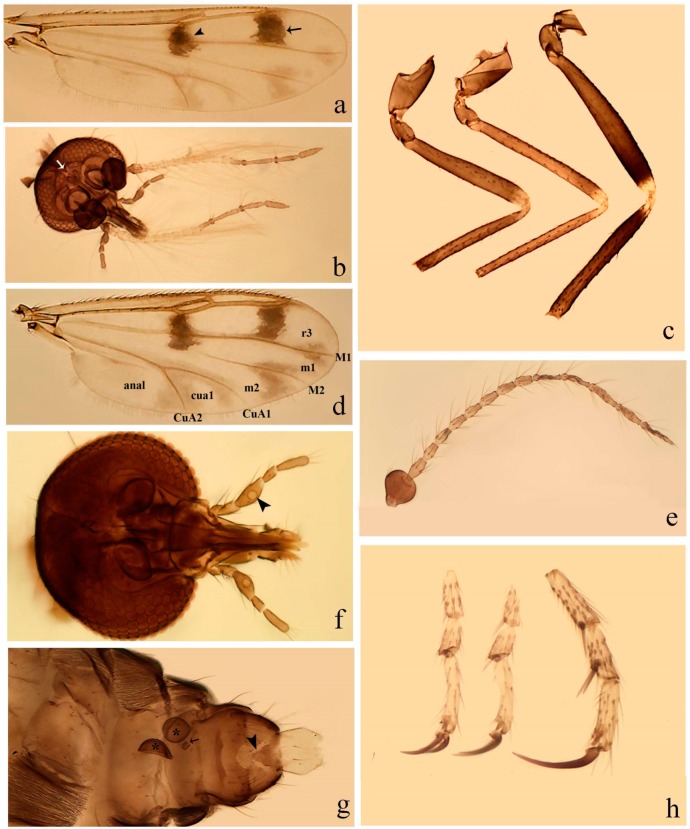
*Downeshelea alia* sp. nov., male: (**a**) wing; arrow: black spot in r_3_; arrowhead: black spot over r-m crossvein; (**b**) head, anterior view; arrow: eyes separation; (**c**) fore-, mid-, hind legs (left to right), lateral view. Female: (**d**) wing; (**e**) antenna; (**f**) head, anterior view; arrowhead: palpal pit; (**g**) apex of abdomen, ventral view; asterisks: spermathecae; arrow: 3rd rudimentary spermatheca; arrowhead: genital sclerite; (**h**) fore-, mid-, hind legs claws (left to right), lateral view.

Figure 3a–h, Figure 6c,d, and Figure 10; Table 1.

*Monohelea stonei:* Lane and Wirth, 1964 [6]: 222 (in part, Colombia record).

**Diagnosis**. Male: only species of *Downeshelea* in the Americas with the following combination of character states: r_3_ without apical grayish spot (Figure 3a); legs brown, hind femur darker subapically (Figure 3c); gonostylus swollen in basal ½ (Figure 6c); paramere stem expanded laterodistally (Figure 6d); distal portion bent on two directions, the posterior one externally directed (Figure 6d); aedeagus subtriangular; distal portion forming a bilobed fringed apex (Figure 6c). Female: only species of *Downeshelea* in the Americas with the following combination of character states: wing (Figure 3d) and legs as described for male; hind leg claw 1.16–1.63 as long as tarsomere 5 (Figure 3h); slightly unequal spermathecae (Figure 3g).

**Description. Male. Head** (Figure 3b). Eyes separated dorsomedially by 1.5× width of an ommatidium (Figure 3b). Antenna pale, except basal portion of flagellomere 1, distal portion of 10, flagellomeres 11–13 brown; antennal ratio 0.88–1.00 (0.96, n = 14). Palpus with segment 3 slightly swollen on midportion with broad, deep sensory pit (as in female, Figure 3f); palpal ratio 2.20–2.80 (2.53, n = 15). **Thorax.** Scutum brown, without definite pattern in slide mounted specimens; pleura pale brown. Wing (Figure 3a) with three grayish areas: first near apex of M_1_ extending into r_3_, m_1_ not reaching wing margin, second large, extending from m_1_ to wing margin in m_2_, third over mediocubital fork, CuA_1_, CuA_2_ extending into cua_1_, anal cell, reaching wing margin; 2nd radial cell twice length of 1st; length 0.95–1.15 (1.04, n = 16) mm, width 0.30–0.40 (0.35, n = 16) mm, costal ratio 0.76–0.82 (0.79, n = 16). Halter pale, distal portion of knob darker. Legs (Figure 3c) brown, hind leg darker, hind femur darker subapically; hind tibial comb without spines. Foretarsomere 1 with one basal, one apical spine; midtarsomere 1 without ventral spines; apical spines of tarsomeres 2–4 of fore-, mid-, hind legs 2-2-3, 2-2-3 (4), 1-1-3 (2); foretarsal ratio 2.30–2.50 (2.41, n = 16), midtarsal ratio 2.43–2.70 (2.51, n = 16), hind tarsal ratio 2.09–2.31 (2.18, n = 16); claws 0.40 (n = 16) length of their respective tarsomere 5. **Abdomen.** Brown. Terminalia (Figure 6c): tergite 9 with quadrate apex, apicolateral process short; sternite 9 concave anteriorly, posterior margin with prominent convex median lobe bearing 2–3 long setae. Gonocoxite dark brown, stout, 2.03–2.48 (2.30, n = 15) times longer than basal width; gonostylus brown, nearly straight, swollen in basal ½ bearing one short setae, 0.65–0.78 (0.71, n = 16) length of gonocoxite. Parameres (Figure 6d) 0.85–1.00 (0.93, n = 16) length of aedeagus, fused on basal portion for 0.19–0.24 (0.22, n = 16) of total length, each with trilobed basal arm; knob bulbous; stem stout, sinuous, expanded laterodistally; distal portion (Figure 6d) curved, slightly broader basally, bent on two directions, the posterior one externally directed, tapering to curved tip, 0.35–0.50 (0.44, n = 15) of total length. Aedeagus (Figure 6c) subtriangular, sclerotized laterally, basal arch U-shaped, extending to 0.25–0.39 (0.32, n = 15) of total length; distal portion with deep mesal notch, forming a bilobed fringed apex (Figure 6c).

**Female**. Similar to male with usual sexual differences; antenna (Figure 3e) brown, basal portion of flagellomeres 2–8 slightly pale; antennal ratio 1.00–1.13 (1.06, n = 9); palpal ratio 2.00–2.40 (2.21, n = 11) (Figure 3f); mandible with 8–10 teeth. Wing as in Figure 3d; length 0.95–1.15 (1.06, n = 11) mm; width 0.40–0.47 (0.46, n = 11) mm, costal ratio 0.80–0.84 (0.83, n = 11). Foretarsomere 1 with one apical, two basal spines; midtarsomere 1 without ventral spines; apical spines of tarsomeres 2–4 of fore-, mid legs: 3-3-4, 2-2-3; foretarsal ratio 2.42–2.65 (2.52, n = 11), midtarsal ratio 2.40–2.84 (2.62, n = 11), hind tarsal ratio 2.45–2.77 (2.56, n = 11); fore-, mid legs claws 0.63–0.75 (0.68, n = 11) length of their respective tarsomeres 5; hind leg claw 1.16–1.63 (1.40, n = 11) as long as tarsomere 5 (Figure 3h). Two slightly unequal spermathecae (Figure 3g), measuring 55–70 (62, n = 10) by 45–52 (47, n = 3) and 47–60 (55, n = 9) by 40–47(43, n = 6) µm. Third rudimentary spermatheca measuring 8.7 µm (Figure 3g).

**Specimens examined.** Holotype male, on microscope slide, labeled “Holotype *Downeshelea alia* Santarém, Borkent and Felippe-Bauer”, “Costa Rica, Limón, Parque Nacional Tortuguero, Sendero real a Agua fría, 98 m, 14–21 August 2004, Malaise, Porras, Gamboa, Briceno, Moraga and Cardenas cols.”(MNCR); allotype female, on microscope slide, labeled “Allotype *Downeshelea alia* Santarém, Borkent and Felippe-Bauer”, “*Downeshelea stonei* (Wirth)”, “Colombia, Rio Raposo, May 1964, light trap, V.H. Lee col.”(USNM). Paratypes labeled as follows: 1 male, on microscope slide, same data as holotype except, “3 km E. Cahuita, 30 October 1993, Borkent” (CNCI); 1 male, on microscope slide, “Puntarenas, Golfito, RVS Rio Piro, Estación Tuva, 40 m, 18 April 2004, Red Noyes, Moraga col.” (MNCR); 1 male on microscope slide, same data except “Finca Catalino, 200 m, 14–21 September 2004, malaise, Gamboa, Briceño, Moragas, Cardenas cols.” (MNCR); 1 male, on microscope slide, same data except “Parque Nacional Corcovado, Estación Los Patos, Sendero a Sirena, 70 m, 25 December 2000–13 February 2001, Malaise, Azofeifa col.” (CCER); 1 male, on microscope slide, same data except “San Pedrillo, Osa pensinsula, 12 August 2001, A. Borkent col.” (CNCI); 2 males, on microscope slide, “Guanacaste, Nosara, Reserva Privada Nosara, Estación, 5 m, 15 June 2004, Red Noyes, Briceno col.” (1 CCER; 1 MNCR); 2 males, on microscope slide, same data except “Desembocadura Rio Nosara, red golpe, Cardenas col.” (MNCR); 1 male, on microscope slide, same data except “Ostional, Rio Montana, 100 m, 13 June 2004, light tap, Gamboa, Briceno, Moraga and Cardenas cols.” (MNCR); 1 male, 2 females, on microscope slide, “Heredia, La selva Biol. Sta. Pto. Viejo de la Sarapiqui, 23 April 1989, Brown col.” (CNCI); 1 female, on microscope slide, same data except “40 m, 18 May 1989” (CCER); 1 female, on microscope slide, same data except “7 May 1989” (MNCR); 1 female, on microscope slide, same data as allotype (USNM); 1 male, on microscope slide, same data except “July 1963” (USNM); 2 males, 2 females, on microscope slide, same data except “August 1963” (1 male CCER; 1 male, 2 females USNM); 1 male, 1 female, on microscope slide, same data except “July 1963” (USNM); 1 male, on microscope slide, same data except “I.1964” (USNM); 4 females, on microscope slide, same data except “March 1964” (2 CCER; 2 USNM); 1 female, on microscope slide, same data except “April 1964” (USNM); 1 male, on microscope slide, same data except “15 April 1964” (USNM); 2 males, on microscope slide, same data except “1 July 1964” (1 CCER; 1 USNM); 1 female, on microscope slide, same data except “28 July 1964” (USNM); 1 male, on microscope slide, same data except “February 1965” (USNM); 2 males, Canada, Ontario, 5 km E. Erieau, Rondeau Provincial Park, 10 July 1984, A. Borkent” (CNCI).

**Distribution and bionomics.** This species is known from Canada, Costa Rica (Guanacaste, Heredia, Puntarenas, and Limón) and Colombia in forested and mangrove areas (Figure 10). In Costa Rica, it has been found from 5 to 100 m above sea level. In other countries it has been found at sea level.

**Etymology.** This species name refers to its similarity to *D. stonei* but being another species (Latin—alia = other).

**Taxonomic discussion.** This species is similar to congeneric species *D. capra* sp. nov. and *D. stonei* by virtue of body and legs coloration and paramere with their distal portion bent on two directions. It can be distinguished by the smaller length of the wing (wing longer in *D. stonei*), the gonostylus swollen on its basal ½ (nearly straight in *D. capra* and *D. stonei*), paramere expanded laterodistally (tapering apically in *D. capra* and expanded subapically in *D. stonei*) and with its posterodistal portion externally directed (internally directed, forming a horn-like process in *D. capra* and without modification in *D. stonei*), and the distal portion of the aedeagus with a bilobed, fringed apex (bifid, not fringed in *D. capra* and *D. stonei*). Although we also observed some differences in some measurements of these three species, we recognize that these may be due to geographical variation. The Canadian specimens were previously identified as *D. stonei* and this record indicates that the distribution of *D. alia* is broad. Thus, more studies are needed to clarify if *D. alia* and *D. stonei* occur in sympatry, primarily in the Nearctic region, where *D. stonei* is widely distributed.

#### 3.4.2. *Downeshelea avizi* sp. nov.

**Figure 4:**
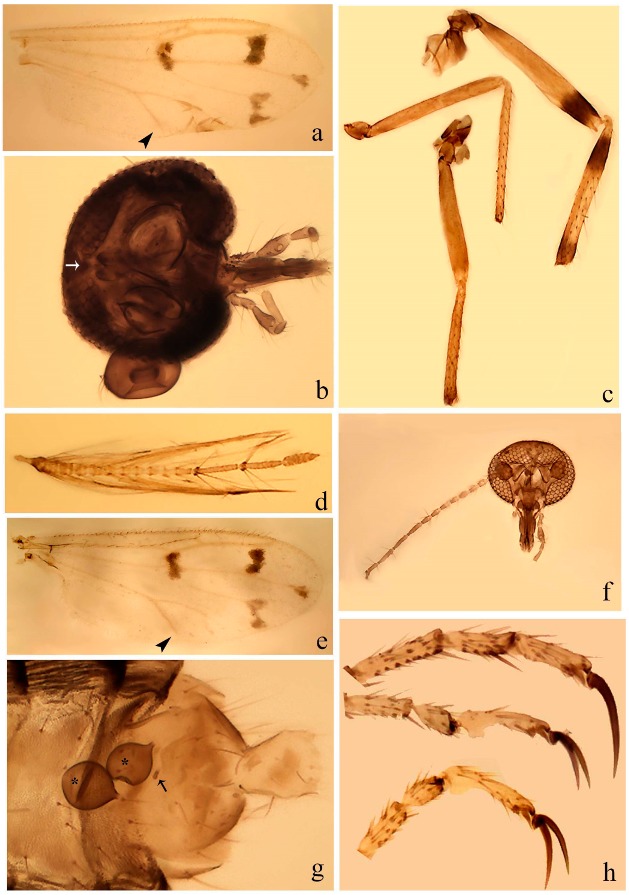
*Downeshelea avizi* sp. nov., male: (**a**) wing; arrowhead: CuA_2_; (**b**) head, anterior view; arrow: eyes separation; (**c**) fore-, mid-, hind legs (left to right), lateral view; (**d**) antenna. Female: (**e**) wing, arrowhead: CuA_2_; (**f**) head, anterior view; (**g**) apex of abdomen, ventral view; asterisks: spermathecae; arrow: 3rd rudimentary spermatheca; (**h**) fore-, mid-, hind legs claws (bottom to top), lateral view.

Figure 4a–h, Figure 6e,f, and Figure 10; Table 1.

**Diagnosis**. Male: only species of *Downeshelea* in the Americas with the following combination of character states: wing without grayish areas in CuA_1,_ CuA_2_ (Figure 4a); r_3_ without apical grayish spot (Figure 4a,e); legs yellowish brown, hind femur with distinct dark band basally, subapically, hind tibia subbasally, apically (Figure 4c); paramere stem expanded basally, tapering medially, gradually expanded apically (Figure 6f); distal portion (Figure 6f) bent, anteriorly directed, broad; aedeagus basal arch V-shaped (Figure 6e). Female: only species of *Downeshelea* in the Americas with the following combination of character states: wing (Figure 4e) and legs as described for male; hind leg claw 1.69 as long as 5th tarsomere (Figure 4h); unequal spermathecae (Figure 4g).

**Description. Male**. **Head** (Figure 4b). Eyes separate dorsomedially by 2× width of an ommatidium (Figure 4b). Antenna (Figure 4d) pale, except basal portion of flagellomere 1, flagellomeres 11–13 brown; antennal ratio 1.13 (n = 1). Palpus with segment 3 slightly swollen on midportion with small, deep sensory pit; palpal ratio 2.60 (n = 1). **Thorax**. Scutum yellowish brown with two anteromedian, two posteromedian elliptical pale areas, pleura yellowish brown. Wing (Figure 4a) with two grayish areas: first near apex of M_1_, not reaching wing margin; second, I-shaped, extending from m_1_ to wing margin in m_2_; 2nd radial cell 2.7 length of 1st; length 1.35 (n = 2) mm; width 0.52 (n = 2) mm; costal ratio 0.80–0.81 (0.81, n = 2). Halter pale, distal portion of knob darker. Legs (Figure 4c) yellowish brown, hind femur with dark band basally, strong dark band subapically, hind tibia with subbasal, apical dark bands; hind tibial comb with 6 spines. Foretarsomere 1 with one basal, two apical spines; midtarsomere 1 with three ventral spines; apical spines of tarsomeres 2–4 of fore-, mid-, hind legs: 3-3-3, 2-2-3, 1-1-2; foretarsal ratio 2.16–2.17 (2.17, n = 2), midtarsal ratio 2.20 (n = 1), hind tarsal ratio 2.03–2.10 (2.07, n = 2); claws 0.39–0.43 (0.41, n = 2) length of their respective tarsomere 5. **Abdomen**. Pale brown. Terminalia (Figure 6e): tergite 9 nearly concave medially, apicolateral process long, broad; sternite 9 straight anteriorly, posterior margin with prominent convex median lobe bearing two long setae. Gonocoxite brown, moderately stout, 2.32–2.40 (2.36, n = 2) times longer than basal width; gonostylus brown, nearly straight, swollen apically, tip blunt, 0.71–0.73 (0.72, n = 2) length of gonocoxite. Parameres (Figure 6f) 1.35–1.43 (1.39, n = 2) length of aedeagus, fused on basal portion for 0.18–0.21 (0.19, n = 2) of total length, each with basal arm trilobed, knob bulbous; stem expanded basally in inner portion, tapering medially, gradually expanded apically; distal portion (Figure 6f) abruptly bent, straight, broad, tapering to pointed tip, directed anteriorly, 0.47 (n = 1) of total length. Aedeagus (Figure 6e) subrectangular, heavily sclerotized laterally, basal arch V-shaped, extending to 0.40–0.48 (0.44, n = 2) of total length; distal portion with deep mesal notch and two sclerotized, slightly serrate processes.

**Female**. Similar to male with usual sexual differences; antenna brown; basal portion of flagellomeres 2–8 paler; antennal ratio 1.12 (n = 1); palpal ratio 2.33–2.80 (2.51, n = 3) (Figure 4f), mandible with 11 teeth. Wing as in Figure 4e; length 1.35–1.37 (1.36, n = 2) mm; width 0.53–0.55 (0.54, n = 2) mm; costal ratio 0.83–0.84 (0.83, n = 2). Foretarsomere 1 with one basal, one apical spine; midtarsomere 1 with 3–4 ventral spines; apical spines of tarsomeres 2–4 of fore-, mid legs: 2-2-3, 2-2-3; foretarsal ratio 2.33 (n = 2), midtarsal ratio 2.37–2.52 (2.47, n = 3), hind tarsal ratio 2.46–2.60 (2.53, n = 3); fore-, mid legs claws 0.73 (n = 1) length of their respective tarsomeres 5; hind leg claw 1.69 (n = 1) as long as tarsomere 5 (Figure 4h). Two unequal spermathecae (Figure 4g) measuring 60–63 (61, n = 2) by 50–53 (51, n = 2) µm and 48–53 (50, n = 2) by 45–48 (46, n = 2) µm. Third rudimentary spermatheca measuring 8.7 µm (Figure 4g).

**Specimens examined.** Holotype male, on microscope slide, labeled “Holotype *Downeshelea avizi* Santarém, Borkent and Felippe-Bauer”, “Brazil, Pará, Tracuateua, Vila de Santa Maria (01°01′45″ S; 46°57′21″ W), 28–29 February 2007, CDC light trap, peridomicilio, Trindade, Gorayeb and Guimarães col.” (MPEG). Allotype female labeled “Allotype *Downeshelea avizi* Santarém, Borkent and Felippe-Bauer”, same data as holotype except “27–28 February 2007, Gorayeb and Guimarães col.” (MPEG). Paratypes labeled as follows: 1 male, 1 female, on microscope slide, same data as holotype (1 male CCER; 1 female MPEG); 2 females, on microscope slide, same data as allotype (CCER; MPEG).

**Distribution and bionomics.** This species is known only from the Brazilian state of Pará. It has been found in forested areas (Figure 10). The locality of Tracuateua is up to 20 m above sea level.

**Etymology.** This species name refers to Gregório Aviz, owner of the property where it was collected.

**Taxonomic discussion.***Downeshelea avizi* most closely resembles *D. chiapasi*, *D. colombiae*, *D. gladius*, and *D. spatha*, in the pattern of dark bands on the hind legs and by the absence of a grayish spot in the distal portion of r_3_. The females of *D. avizi* can be easily distinguished by the pale CuA_2_ (CuA_2_ with grayish spot in the other aforementioned species), presence of basal dark band on hind femur (absent in *D. chiapasi*, *D. colombiae*, and *D. gladius*), the unequal spermathecae (equal-sized in *D. chiapasi*, subequal in *D. spatha*) and by the long hind leg claw (shorter in the other species). The male can be distinguished by the fused parameres (parameres separated in *D. chiapasi*), apical process of the paramere straight (nearly straight and subapical in *D. chiapasi*, deeply curved and subapical in *D. colombiae*, sinuous and apical in *D. gladius*) and the paramere longer than aedeagus (nearly similar in length *D. colombiae, D. gladius,* and *D. spatha*).

#### 3.4.3. *Downeshelea bahiana* sp. nov.

**Figure 5:**
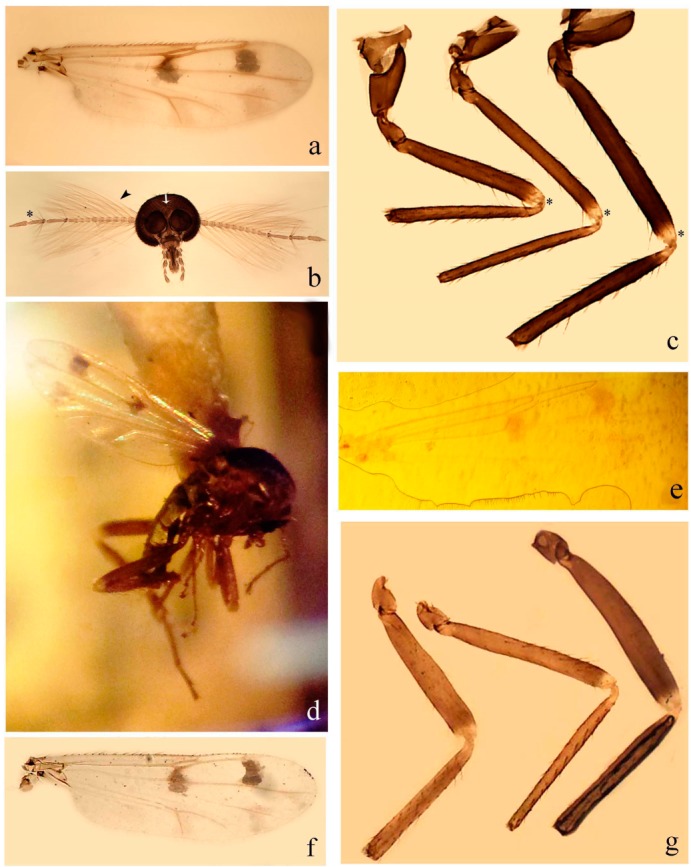
*Downeshelea bahiana* sp. nov., male: (**a**) wing; (**b**) head, anterior view; arrow: eyes separation; asterisk: antennal distal segments; arrowhead: antennal plume; (**c**) fore-, mid-, hind legs (left to right), lateral view; asterisks: femorotibial joint areas. *Downeshelea balboa*, male: (**d**) paratype male pinned specimen; (**e**) wing. *Downeshelea bicornis*, male: (**f**) wing; (**g**) fore-, mid-, hind legs (left to right), lateral view.

**Figure 6:**
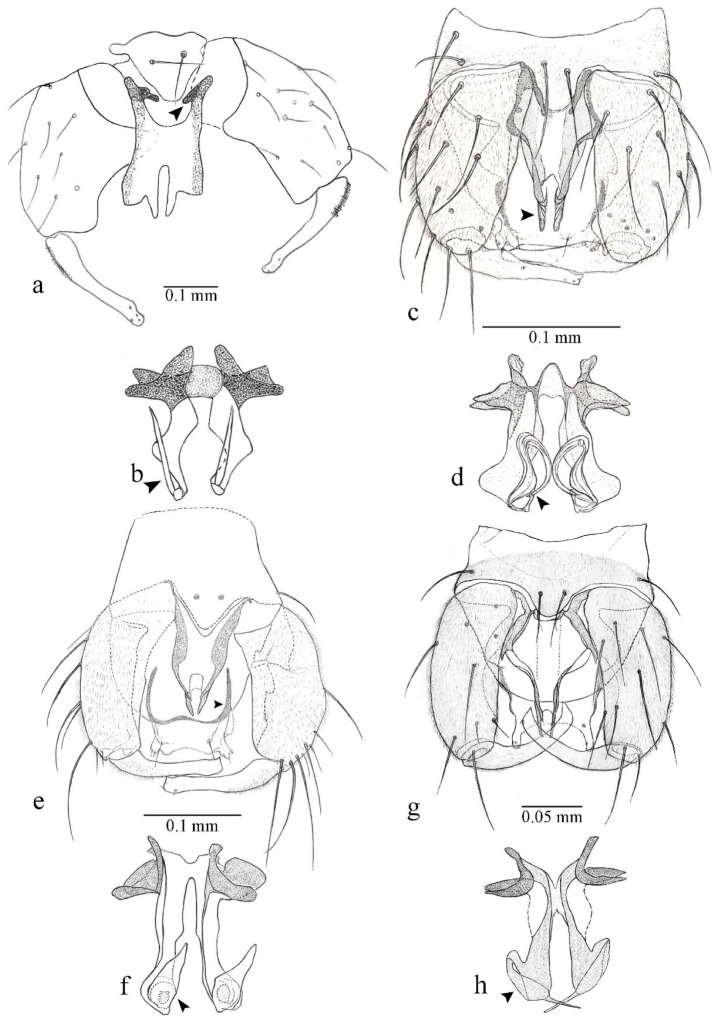
*Downeshelea blantoni*, male: (**a**) terminalia with aedeagus, ventral view; arrowhead: sclerotized area; (**b**) parameres, ventral view; arrowhead: distal portion. *Downeshelea alia* sp. nov., male: (**c**) terminalia with aedeagus, ventral view; arrowhead: aedeagus fringed apex; (**d**) parameres, ventral view; arrowhead: distal portion. *Downeshelea avizi* sp. nov., male: (**e**) terminalia with aedeagus, ventral view; arrowhead: tergite 9 lateral sclerotized band; (**f**) parameres, ventral view; arrowhead: distal portion. *Downeshelea bahiana* sp. nov., male: (**g**) terminalia with aedeagus, ventral view; (**h**) parameres, ventral view; arrowhead: distal portion.

Figure 5a–c, Figure 6g,h, and Figure 10; Table 1.

**Diagnosis.** Male: only species of *Downeshelea* in the Americas with the following combination of character states: r_3_ without apical grayish spot (Figure 5a); legs brown (Figure 5c); paramere stem expanded distally (Figure 6h); subapical portion with long process bent, slender basally, expanded on midportion, abruptly tapering to long pointed tip, posteromesally directed (Figure 6h); aedeagus subtriangular, expanded mesally, tapering distally; basal arch U-shaped (Figure 6g). Female unknown.

**Description. Male**. **Head** (Figure 5b). Eyes contiguous in lower portion (Figure 5b). Antenna pale except basal portion of flagellomere 1, distal portion of flagellomere 10, flagellomeres 11–13 brown (Figure 5b); antennal ratio 0.96. Palpus with segment 3 slightly swollen on midportion with small, shallow sensory pit; palpal ratio 2.50. **Thorax**. Scutum, postscutellum brown; pleura pale brown. Wing (Figure 5a) with three grayish areas: first near apex of M_1_, not reaching wing margin; second extending from m_1_ to wing margin in m_2_; third over CuA_1_, CuA_2_ extending from mediocubital fork into cua_1,_ anal cell, reaching wing margin; 2nd radial cell 1.8 length of 1st; length 1.22 mm; width 0.40 mm; costal ratio 0.73. Halter brown. Legs (Figure 5c) brown, hind leg darker; hind tibial comb with 7 spines. Foretarsomere 1 with one basal, one apical spine; midtarsomere 1 with three ventral spines; apical spines of tarsomeres 2–4 of fore-, mid-, hind legs: 2-2-3, 2-2-3, 1-1-2; foretarsal ratio 2.32, midtarsal ratio 2.48, hind tarsal ratio 2.25; claws 0.38 length of their respective tarsomere 5. **Abdomen**. Brown. Terminalia (Figure 6g): tergite 9 with quadrate apex, apicolateral process long, slender; sternite 9 slightly concave anteriorly, posterior margin with moderately convex median lobe bearing two long setae. Gonocoxite brown, moderately stout, 2.03 times longer than basal width; gonostylus brown, curved, broad basally, 0.71 length of gonocoxite. Parameres (Figure 6h) 1.00 length of aedeagus, fused on basal portion for 0.19 of total length, each with basal arm trilobed, knob slender; stem expanded distally, subapical portion (Figure 6h) with long hyaline process bent, slender basally, expanded on midportion, abruptly tapering to long pointed tip, posteromesally directed, 0.48 of total length. Aedeagus (Figure 6g) subtriangular, expanded mesally, tapering distally; basal arch U-shaped, heavily sclerotized, extending to 0.25 of total length; distal portion with deep mesal notch and two sclerotized pointed processes.

**Female**. Unknown.

**Specimens examined.** Holotype male, on microscope slide, labeled “Holotype *Downeshelea bahiana* Santarém, Borkent and Felippe-Bauer”, “Brazil, Bahia, Estação Ecológia Wenceslau Guimarães” (CCER).

**Distribution and bionomics.** This species is known only from Brazilian state of Bahia (Figure 10). It has been found in forested areas up to 150 m above sea level.

**Etymology.** This species name refers to Bahia, the Brazilian state where it was collected.

**Taxonomic discussion.** The shape of the aedeagus of this species is unique in the genus. The wing and leg patterns resemble those in the *multilineata* species group, but in *D. bahiana* the paramere has a subapical process posteriorly directed, not a distal one anteriorly directed as in the *multilineata* group.

#### 3.4.4. *Downeshelea balboa* (Lane and Wirth)

Figure 5d,e, Figure 9a–c, and Figure 10; Table 1.

*Monohelea balboa* Lane and Wirth, 1964 [6]: 225 (male, female, Panama); Wirth, 1974 [3]: 40 (in catalog south of the USA; distribution).

*Downeshelea balboa* Wirth and Grogan, 1988 [7]: 51 (combination); Borkent and Wirth, 1997 [24]: 97 (in World catalog); Borkent and Spinelli, 2000 [25]: 47 (in catalog south of the USA; distribution); Borkent and Spinelli, 2007 [26]: 80 (in Neotropical catalog; distribution); Borkent, 2016 [22]: 124 (in World catalog).

**Diagnosis**. Male: only species of *Downeshelea* in the Americas with the following combination of character states: r_3_ without apical grayish spot (Figure 5e); legs brown (Figure 5d); parameres separated, basal arm bilobed, stem curved externally, tapering to apex (Figure 9c); aedeagus rectangular, poorly sclerotized, basal arch quadrate (Figure 9b). Female not diagnosable.

**Redescription. Male** (Figure 5d). Wing (Figure 5e) with three grayish areas: first near apex of M_1_, second sigmoid-shaped, extending from m_1_ to wing margin in m_2;_ third over CuA_2_ reaching wing margin; 2nd radial cell twice length of 1st; length 1.11 mm; width 0.36 mm; costal ratio 0.76. Halter knob infuscated. Legs brown, hind leg darker. **Abdomen**. Brown. Terminalia (Figure 9a): tergite 9, sternite 9 not visible in slide mounted specimen. Gonocoxite brown, stout, 1.80 times longer than basal width; gonostylus brown, nearly straight, 0.78 length of gonocoxite. Parameres (Figure 9c) 1.05 length of aedeagus, separated, each with basal arm bilobed, stem curved externally, tapering to apex. Aedeagus (Figure 9b) rectangular, poorly sclerotized, basal arch quadrate, extending to 0.23 of total length, distal portion with mesal notch ending in two lateral rounded lobes.

**Female.** Similar to the male with usual sexual differences. Antennal ratio 1.02 (n = 1). Wing length 1.13–1.26 (1.19, n = 2) mm; costal ratio 0.79 (n = 1). Hind tarsal ratio 2.50 (n = 1).

**Specimens examined.** 1 male, pinned, with genitalia and wing in drop of Canada Balsam on celluloid strip on pin, labeled “Paratype, *Monohelea balboa* Lane and Wirth, drawn”, “Panama, Aquadulce, Cocle Prov., 25 April 1951, light trap, FS Blanton col.”(FSP); 1 female, pinned, with wing in drop of Canada Balsam on celluloid strip on pin, labeled “Paratype, *Monohelea balboa* Lane and Wirth”, same data except “Mojinga Swamp, Fort Sherman, Canal Zone, 24 October 1951” (FSP); 1 female, pinned, labeled “Paratype, *Monohelea balboa* Lane and Wirth”, same data except “La Jolla, September 1951” (FSP).

**Distribution and bionomics.** This species is known only from coastal areas in Panama (Figure 10), occurring up to 30 m above sea level.

**Taxonomic Discussion.** This species has a relatively simple male genitalia that readily distinguishes it from other species in the genus. *Downeshelea carioca, D. fluminensis* and *D. quasidentica* also have legs without bands and separate parameres, but in these three species the CuA_1_ is grayish. *Downeshelea balboa* has a rectangular aedeagus (triangular in *D. fluminensis* and *D. carioca*, quadrangular in *D. quasidentica*) and simple paramere (paramere with median processes in *D. fluminensis* and *D. carioca*). We studied the three paratypes deposited in Faculdade de Saúde Pública de São Paulo. The holotype and allotype of *D. balboa* are pinned specimens in the USNM (#66441) and no further observations were made of them. Some slide mounted specimens from Belize, El Salvador and Honduras, previously labeled as *D. balboa* by Wirth, are here identified as *D. fluminensis*.

#### 3.4.5. *Downeshelea bicornis* Felippe-Bauer and Quintelas

Figure 5f,g, Figure 9d–f, and Figure 10; Table 1.

*Downeshelea bicornis* Felippe-Bauer and Quintelas, 1993 [15]: 185 (male, Brazil); Borkent and Wirth, 1997 [24]: 97 (in World catalog); Borkent and Spinelli, 2000 [25]: 47 (in catalog south of the USA; distribution); Borkent and Spinelli, 2007 [26]: 80 (in Neotropical catalog; distribution); Borkent, 2016 [22]: 124 (in World catalog); Santarém and Felippe-Bauer, 2019 [2]: 16 (Brazilian distribution).

**Diagnosis.** Male: only species of *Downeshelea* in the Americas with the following combination of character states: r_3_ without apical grayish spot (Figure 5f); legs brown (Figure 5g); paramere (Figure 9f) submedian area with a channel, midportion swollen, with slender median, apical (Figure 9f) horn-like processes equally sized; aedeagus rectangular, distal portion with two longs, narrow sclerotized lobes (Figure 9e). Female unknown.

**Specimens examined.** 1 male, on microscope slide, labeled “Holotype, *Downeshelea bicornis* Felippe-Bauer and Quintelas”, “Brazil, Rio de Janeiro, Rio Bonito, Centro, R. Getúlio Vargas 109, 29 March 1989, FEEMA cols.” (CCER).

**Distribution.** This species is known only from Brazil (Rio de Janeiro) occurring in a humid area at 60 m above sea level (Figure 10).

#### 3.4.6. *Downeshelea bifida* sp. nov.

**Figure 7:**
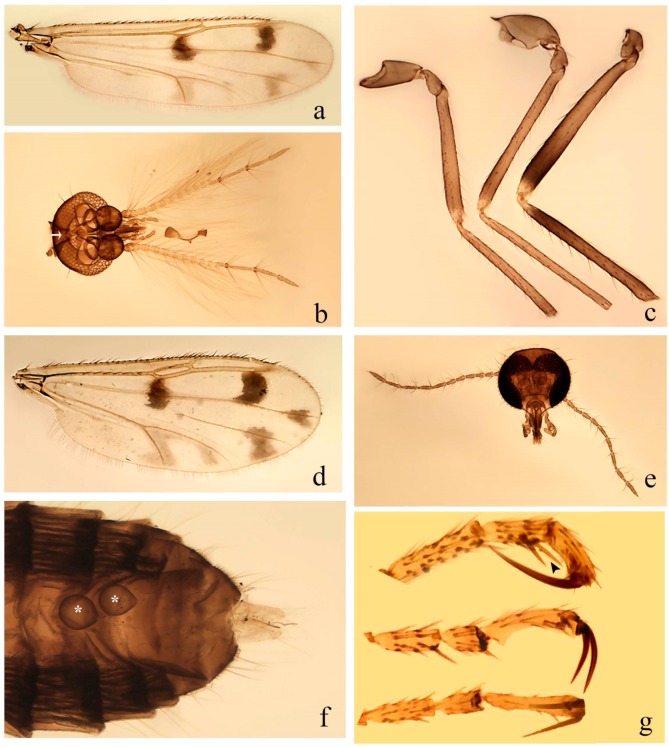
*Downeshelea bifida* sp. nov., male: (**a**) wing; (**b**) head, anterior view; arrow: eyes separation; (**c**) fore-, mid-, hind legs (left to rigth), lateral view. Female: (**d**) wing; (**e**) head, anterior view; (**f**) apex of abdomen, ventral view; asterisks: spermathecae; (**g**) fore-, mid-, hind legs claws (bottom to top), lateral view; arrowhead: apical spines of tarsomere 4.

Figure 7a–g, Figure 9g–I, and Figure 10; Table 1.

**Diagnosis**. Male: only species of *Downeshelea* in the Americas with the following combination of character states: r_3_ without apical grayish spot (Figure 7a); legs brown, fore-, mid-, hind femur darker subapically, hind tibia darker subbasally (Figure 7c); paramere (Figure 9i) divergent with distal portion bifid, the inner projection with tip bent ventrally to sharp point (Figure 9i); aedeagus (Figure 9h) rectangular, with pair of submedian sclerotized elliptical areas (Figure 9h). Female: only species of *Downeshelea* in the Americas with the following combination of character states: wing (Figure 7d) and legs as described for male, hind leg claw 1.18–1.43 as long as tarsomere 5 (Figure 7g); equal-sized spermathecae (Figure 7f).

**Description. Male**. **Head** (Figure 7b). Eyes separate dorsomedially by width of one ommatidium (Figure 7b). Antenna pale, except basal portion of flagellomere 1, distal portion of flagellomere 10, flagellomeres 11–13 brown; antennal ratio 1.00–1.12 (1.05, n = 8). Palpus with segment 3 swollen on midportion with broad, deep sensory pit; palpal ratio 2.00–2.50 (2.30, n = 8). **Thorax**. Scutum brown, without definite pattern in slide mounted specimens; pleura pale brown. Wing (Figure 7a) with three grayish areas: first near apex of M_1_, not reaching wing margin; second extending from m_1_ to wing margin in m_2_; third over CuA_1_, CuA_2_ extending from mediocubital fork into cua_1,_ anal cell, reaching wing margin; 2nd radial cell twice length of 1st; length 0.92–1.17 (1.05, n = 8) mm; width 0.32–0.37 (0.35, n = 8) mm; costal ratio 0.77–0.81 (0.78, n = 8). Halter pale, distal portion of knob darker. Legs (Figure 7c) brown, hind leg darker, fore-, mid-, hind femur darker subapically, hind tibia darker subbasally; hind tibial comb with 6–7 spines. Foretarsomere 1 with one basal, two apical spines; midtarsomere 1 without ventral spines; apical spines of tarsomeres 2–4 of fore-, mid-, hind legs: 2-3-3(4), 2-2-3, 1-1-2; foretarsal ratio 2.28–2.45 (2.39, n = 8), midtarsal ratio 2.38–2.59 (2.51, n = 8), hind tarsal ratio 2.07–2.27 (2.14, n = 8); claws 0.38–0.47 (0.42, n = 8) length of their respective tarsomere 5. **Abdomen**. Brown, yellowish brown dorsally. Terminalia (Figure 9g): tergite 9 with quadrate apex, apicolateral process short; sternite 9 slightly concave anteriorly, posterior margin with moderately convex median lobe bearing 2–3 long setae. Gonocoxite brown, moderately stout, 1.93–2.12 (2.02, n = 8) times longer than basal width; gonostylus brown, nearly straight, tip flattened, 0.73–0.80 (0.76, n = 8) length of gonocoxite. Parameres (Figure 9i) 1.00–1.06 (1.02, n = 8) length of aedeagus, fused on basal portion for 0.20–0.26 (0.22, n = 6) of total length; each with basal arm trilobed, knob slender; stem sinuous, slightly divergent, tapering distally; distal portion bifid, slender, inner projection with tip bent ventrally to sharp point (Figure 9i). Aedeagus (Figure 9h) rectangular, with pair of submedian, longitudinal sclerotized elliptical areas (Figure 9h), basal arch somewhat U-shaped, heavily sclerotized, extending to 0.14–0.21 (0.17, n = 7) of total length; distal portion with deep mesal notch and two sclerotized serrate processes.

**Female**. Similar to male with usual sexual differences; antenna brown; basal portion of flagellomeres 2–8 slightly pale; antennal ratio 1.03–1.09 (1.06, n = 3); palpal ratio 1.71–2.00 (1.79, n = 3) (Figure 7e); mandible with 10 teeth. Wing as in Figure 7d; length 1.12–1.27 (1.22, n = 3) mm; width 0.45–0.50 (0.49, n = 3) mm; costal ratio 0.80–0.84 (0.82, n = 3). Foretarsomere 1 with one basal, one apical spine; midtarsomere 1 without ventral spines; apical spines of tarsomeres 2–4 of fore-, mid legs: 3-3-4, 2-2-4; foretarsal ratio 2.60–2.80 (2.62, n = 3); midtarsal ratio 2.71–2.76 (2.73, n = 3); hind tarsal ratio 2.31–2.54 (2.44, n = 3); fore-, mid legs claws 0.68–0.78 (0.72, n = 3) length of their respective tarsomeres 5; hind leg claw 1.18–1.43 (1.30, n = 3) as long as tarsomere 5 (Figure 7g). Two equal-sized spermathecae (Figure 7f) measuring 55–60 (58, n = 3) by 48–53 (n = 2) µm. Third rudimentary spermatheca measuring 9.2 µm (not visible in Figure 7f).

**Specimens examined.** Holotype male, on microscope slide, labeled “Holotype *Downeshelea bifida* Santarém, Borkent and Felippe-Bauer”, “Colombia, Valle Rio Raposo, February 1965, light trap, VH Lee col.” (USNM); allotype female, on microscope slide, labeled “Allotype *Downeshelea bifida* Santarém, Borkent and Felippe-Bauer”, same data as holotype except “28 July 1964” (USNM). Paratypes labeled as follows: 1 male, on microscope slide, same data as holotype (USNM); 2 females, on microscope slide, same data as allotype (CCER; USNM); 1 male, on microscope slide, same data except March 1964” (CCER); 1 male, on microscope slide, same data except October 1964” (USNM); 3 males, on microscope slide, same data except August 1964” (CCER; USNM); 1 male, on microscope slide, “Costa Rica, Alajuela, San Carlos, Boca Tapada, Laguna Lagarto Lodge, 50–100 m, 23 July–17 April 2004, malaise, B Hernández col.”(MNCR).

**Distribution and bionomics.** This species is known from Costa Rica (Alajuela) and Colombia (Figure 10). It has been found in coastal areas, occurring in Colombia at sea level and in Costa Rica from 50 to 100 m above sea level.

**Etymology.** This species name refers to the bifid distal portion of paramere of the male (Latin—bifida = bifid).

**Taxonomic discussion.** This species has a male with a bifid distal portion of each paramere that most closely resembles *D. divergentis* and *D. castroi* and *D. quechua*. It can be distinguished from these species by the paramere stem divergent from near its base (parallel in *D. castroi*; divergent in distal ½ in *D. divergentis* and *D. quechua*), the rectangular aspect of the aedeagus (Y-shaped in *D. castroi*, subtriangular in *D. divergentis*, subrectangular in *D. quechua*), by the distal portion of the paramere with its inner projection bent ventrally to a sharp point (inner projection gently tapering, not bent in *D. castroi* and *D. divergentis,* inner projection bent as a short claw in *D. quechua*). Also, it can be distinguished from *D. castroi* by the absence of a grayish spot in r_3_ and m_1_.

#### 3.4.7. *Downeshelea capra* sp. nov.

**Figure 8:**
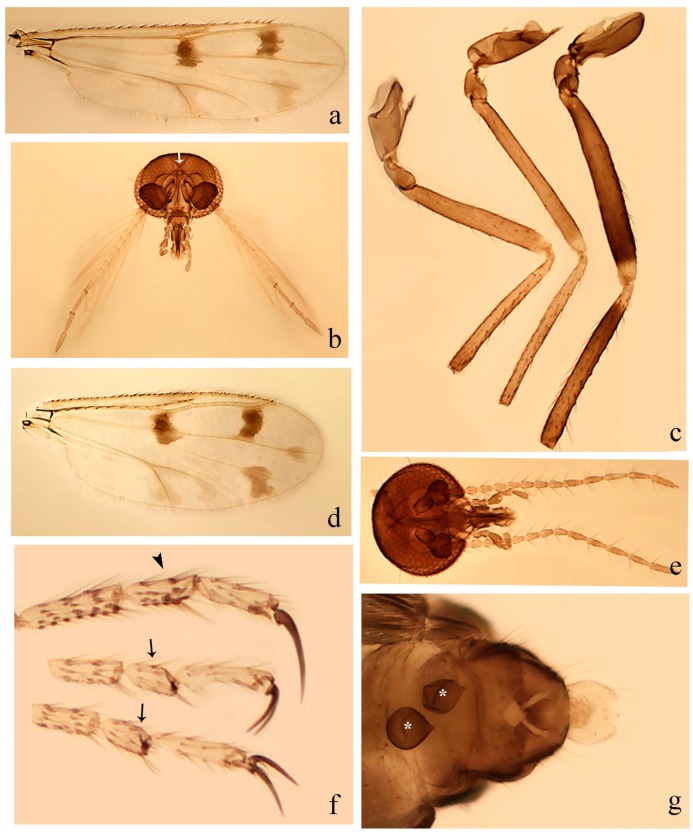
*Downeshelea capra* sp. nov., male: (**a**) wing; (**b**) head, anterior view; arrow: eyes separation; (**c**) fore-, mid-, hind legs (left to right), lateral view. Female: (**d**) wing; (**e**) head, anterior view; (**f**) fore-, mid-, hind legs claws (bottom to top), lateral view; arrow: fore-, midtarsomere 4; arrowhead: hind tarsomere 4; (**g**) apex of abdomen, ventral view; asterisks: spermathecae.

**Figure 9:**
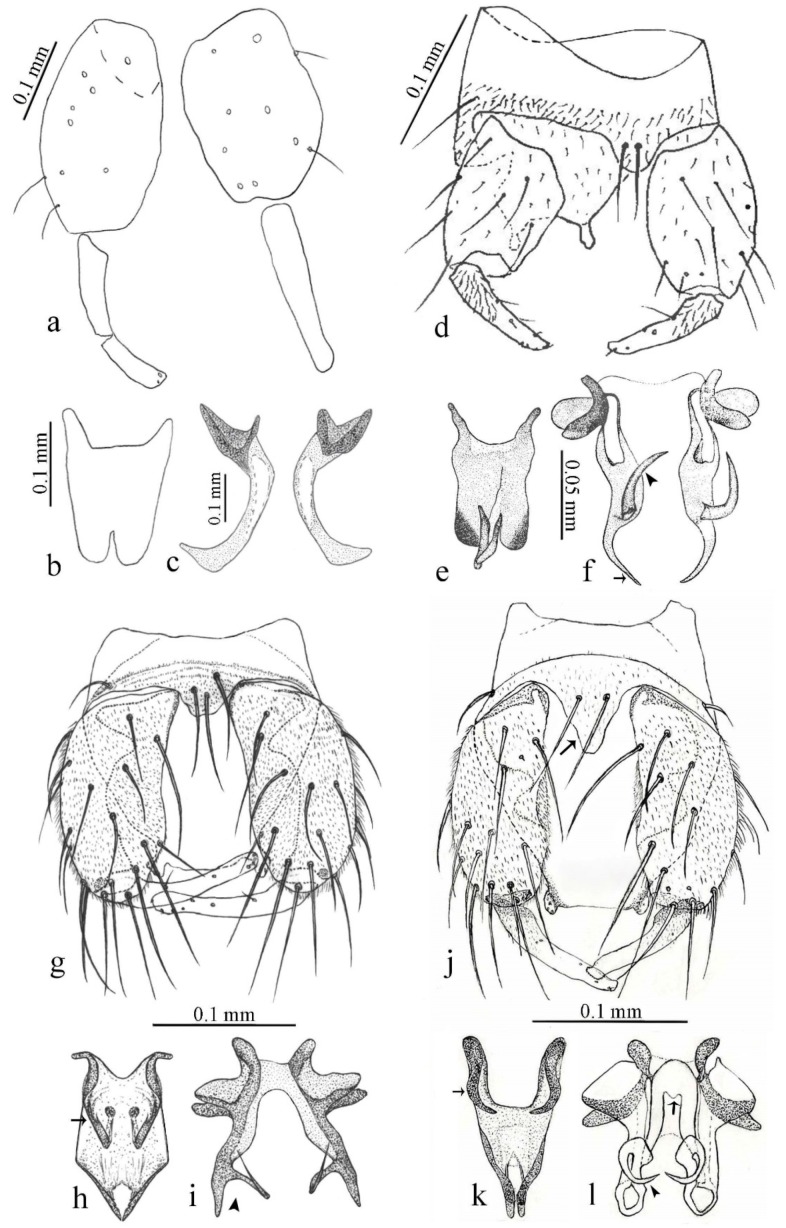
*Downeshelea balboa*, male: (**a**) terminalia, ventral view; (**b**) aedeagus, ventral view; (**c**) parameres, ventral view. *Downeshelea bicornis*, male: (**d**) terminalia, ventral view; (**e**) aedeagus, ventral view; (**f**) parameres, ventral view; arrowhead: median process; arrow: distal portion. *Downeshelea bifida* sp. nov., male: (**g**) terminalia, ventral view; (**h**) aedeagus, ventral view; arrow: sclerotized area; (**i**) parameres, ventral view; arrowhead: distal portion. *Downeshelea capra* sp. nov., male: (**j**) terminalia, ventral view; arrow: sternite 9 convex median lobe; (**k**) aedeagus, ventral view; arrow: sclerotized projection; (**l**) parameres, ventral view; arrowhead: distal portion.

**Figure 10:**
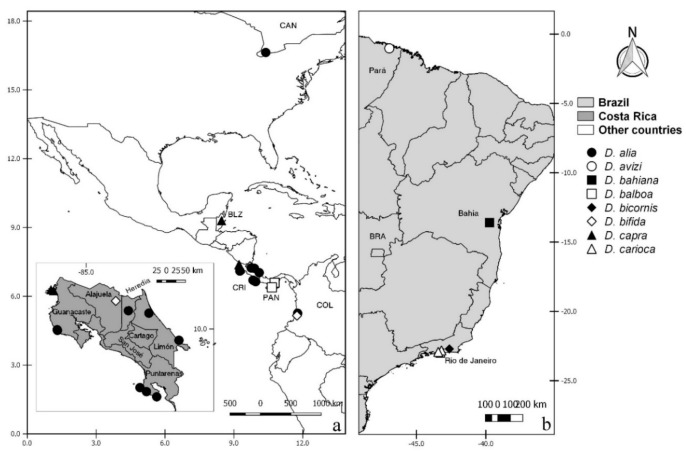
Geographic distribution of *Downeshelea alia* sp. nov. (**a**), *D. avizi* sp. nov. (**b**), *D. bahiana* sp. nov. (**b**), *D. balboa* (**a**), *D. bicornis* (**b**), *D. bifida* sp. nov. (**a**), *D. capra* sp. nov. (**a**), and *D. carioca* (**b**).

Figure 8a–g, Figure 9j–l, and Figure 10; Table 1.

**Diagnosis**. Male: only species of *Downeshelea* in the Americas with the following combination of character states: r_3_ without apical grayish spot (Figure 8a); legs brown, hind femur darker subapically, hind tibia darker subbasally (Figure 8c); distal portion of paramere bent in two directions, the posterior one internally directed, forming horn-like process (Figure 9l); aedeagus basal arm with sinuous sclerotized projection, directed posteriorly (Figure 9k). Female: only species of *Downeshelea* in the Americas with the following combination of character states: wing (Figure 8d) and legs as described for male; hind leg claw 1.07–1.23 as long as tarsomere 5 (Figure 8f); slightly unequal spermathecae (Figure 8g).

**Description. Male**. **Head** (Figure 8b). Eyes separate dorsomedially by width of one ommatidium (Figure 8b). Antenna pale, except basal portion of flagellomere 1, distal portion of flagellomere 10, flagellomeres 11–13 brown; antennal ratio 0.91–1.03 (0.98, n = 15). Palpus with segment 3 slightly swollen on midportion with broad, deep sensory pit; palpal ratio 2.00–2.60 (2.25, n = 15). **Thorax**. Scutum brown, without definite pattern in slide mounted specimens; pleura pale brown. Wing (Figure 8a) with two grayish areas: first sigmoid-shaped, extending from m_1_ to wing margin in m_2_; second over CuA_1_, CuA_2_ extending from mediocubital fork into cua_1,_ anal cell, reaching wing margin; faint grayish area near apex of M_1_ in some specimens; 2nd radial cell 2.3 length of 1st; length 0.91–1.15 (1.01, n = 15) mm; width 0.32–0.37 (0.34, n = 15) mm; costal ratio 0.73–0.77 (0.75, n = 15). Halter pale, distal portion of knob darker. Legs (Figure 8c) brown, hind leg darker, hind femur slightly darker subapically, hind tibia slightly darker subbasally; hind tibial comb with six spines. Foretarsomere 1 with one basal, one apical spine; midtarsomere 1 without ventral spines, one specimen with three basal spines; apical spines of tarsomeres 2–4 of fore-, mid-, hind legs: 2-2-4, 2-2-4, 1-1-2; foretarsal ratio 2.31–2.47 (2.41, n = 14), midtarsal ratio 2.32–2.67 (2.46, n = 15), hind tarsal ratio 2.03–2.31 (2.15, n = 15); claws 0.36–0.46 (0.40, n = 15) length of their respective tarsomere 5.

**Abdomen**. Brown. Terminalia (Figure 9j): tergite 9 with quadrate apex, apicolateral process short; sternite 9 slightly concave anteriorly, posterior margin with prominent convex median lobe bearing 2–3 long setae (Figure 9j). Gonocoxite brown, moderately stout, 2.19–2.46 (2.27, n = 15) times longer than basal width; gonostylus brown, nearly straight, tip truncated, 0.61–0.69 (0.64, n = 15) length of gonocoxite. Parameres (Figure 9l) 0.93–1.05 (0.98, n = 14) length of aedeagus, fused on basal portion for 0.21–0.28 (0.24, n = 14) of total length, each with basal arm trilobed, knob flattened, short posteromedian projection on the medially fused portion (Figure 9l); stem broad basally, tapering apically; distal portion bent in two directions, the posterior one internally directed, forming horn-like process (Figure 9l), 0.42–0.49 (0.46, n = 14) of total length. Aedeagus (Figure 9k) subtriangular, heavily sclerotized laterally, basal arch U-shaped, extending to 0.33–0.43 (0.39, n = 15) of total length, basal arm with sinuous sclerotized projection, directed posteriorly (Figure 9k); distal portion with deep mesal notch and two sclerotized rounded tip processes.

**Female**. Similar to male with usual sexual differences; antenna brown; basal portion of flagellomeres 2–8 slightly pale; antennal ratio 1.00–1.06 (1.03, n = 5); palpal ratio 2.00–2.40 (2.12, n = 8) (Figure 8e); mandible with 8–10 teeth. Wing (Figure 8d) similar to male, except by grayish area over M_1_; length 0.92–0.97 (0.95, n = 8) mm; width 0.37–0.42 (0.40, n = 8) mm; costal ratio 0.79–0.84 (0.82, n = 8). Foretarsomere 1 with one basal, one apical spine; midtarsomere 1 without ventral spines, some specimens with three basal spines; apical spines of tarsomeres 2–4 of fore-, mid legs: 2-2-4, 2-2-3(4); foretarsal ratio 2.42–2.61 (2.53, n = 8), midtarsal ratio 2.33–2.63 (2.57, n = 8), hind tarsal ratio 2.22–2.36 (2.30, n = 8); fore-, mid legs claws 0.56–0.73 (0.66, n = 8) length of their respective tarsomeres 5; hind leg claw 1.07–1.23 (1.16, n = 8) as long as tarsomere 5 (Figure 8f). Two slightly unequal spermathecae (Figure 8g), measuring 55–67 (59, n = 8) by 50 (n = 3) µm and 50–57 (52, n = 8) by 45–47 (46, n = 2) µm. Third rudimentary spermatheca measuring 9 µm (not visible in Figure 8g).

**Specimens examined.** Holotype male, on microscope slide, labeled “Holotype *Downeshelea capra* Santarém, Borkent and Felippe-Bauer”, “Costa Rica, Guanacaste, La Cruz, Parque Nacional Santa Rosa, Sector Murciélago, 5.5 km ENE. del Cerro Guachipelín”, 40 m, 29–27 July 1996, malaise, M. Araya col.” (MNCR); allotype female, on microscope slide, labeled “Allotype *Downeshelea capra* Santarém, Borkent and Felippe-Bauer”, same data as holotype (MNCR). Paratypes labeled as follows: 4 males, 4 females, on microscope slide, same data as holotype (1 male, 1 female CCER; 3 males, 3 females MNCR); 6 males, 2 females, on microscope slide, same data except “27 July–27 August 1996” (1 male, 1 female CCER; 5 males, 1 female MNCR); 2 males, 1 female, on microscope slide, same data except “3 km NNO. del Cerro Guachipelín, 7 February 1996” (MNCR); 1 male, on microscope slide, labeled “*Downeshelea stonei*” (Wirth), “Belize, Hattieville, 8 July 1986, light trap, W.L. Haase col”. (USNM); 1 male adult, on microscope slide, same data except “Mile 15 Western highway, 9 July 1968” (CCER).

**Distribution and bionomics.** This species is known from Belize and Costa Rica (Guanacaste) (Figure 10). It has been found in coastal and forested areas, occurring from 5 m (Belize) to 40 m (Costa Rica) above sea level.

**Etymology.** This species name refers to the similarity of the distal portion of the paramere to the horn of a goat (*Capra*).

**Taxonomic discussion.***Downeshelea capra* most closely resembles *D. stonei* and *D. alia* by the wing pattern of spots and the distal portion of the paramere bent in two directions. Further comments are in the taxonomic discussion of *D. alia* sp. nov.

#### 3.4.8. *Downeshelea carioca* (Tavares and Silva-Pereira)

**Figure 11:**
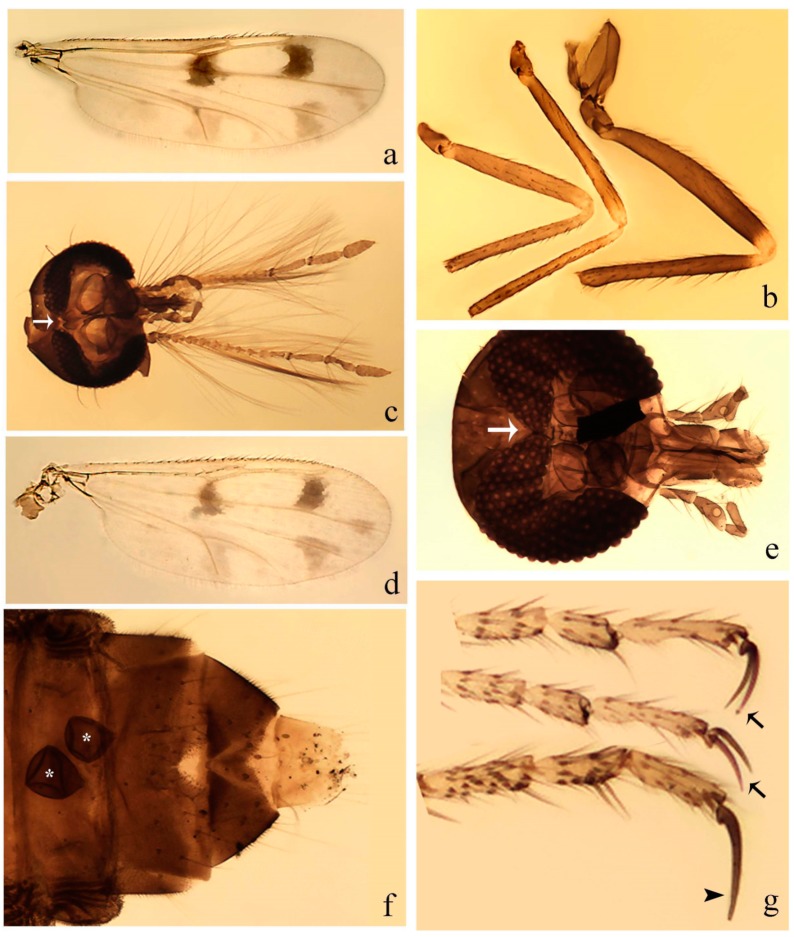
*Downeshelea carioca*, male: (**a**) wing; (**b**) fore-, mid-, hind legs (left to right), lateral view; (**c**) head, anterior view; arrow: eyes separation. Female: (**d**) wing; (**e**) head, anterior view; arrow: eyes separation; (**f**) apex of abdomen, ventral view; asterisks: spermathecae; (**g**) arrow: fore-, mid legs claws; arrowhead hind legs claw (top to bottom), lateral view.

Figure 10, Figure 11a–g, and Figure 14a–c; Table 1.

*Monohelea carioca* Tavares and Silva-Pereira, 1978 [35]: 157 (female, male, Brazil).

*Downeshelea carioca*: Wirth and Grogan, 1988 [7]: 51 (combination); Borkent and Wirth, 1997 [24]: 97 (in World catalog); Borkent and Spinelli, 2000 [25]: 47 (in catalog south of the USA; distribution); Borkent and Spinelli, 2007 [26]: 80 (in Neotropical catalog; distribution); Borkent, 2016 [22]: 124 (in World catalog); Santarém and Felippe-Bauer, 2019 [2]: 16 (Brazilian distribution).

**Diagnosis**. Male: only species of *Downeshelea* in the Americas with the following combination of character states: r_3_ without apical grayish spots (Figure 11a); legs brown, hind femur slightly darker subapically (Figure 11b); tergite 9 with very long apicolateral process (Figure 14a); parameres separated with median lateral process (Figure 14c); aedeagus triangular, basal arch broad (Figure 14b). Female: only species of *Downeshelea* in the Americas with the following combination of character states: wing (Figure 11d) and legs as described for male; hind leg claw 1.17–1.25 as long as tarsomere 5 (Figure 11g); subequal spermathecae (Figure 11f).

**Redescription. Male. Head** (Figure 11c). Eyes separated dorsomedially by 1.5× width of an ommatidium (Figure 11c). Antenna pale, except basal portion of flagellomere 1, distal portion of flagellomere 10, flagellomeres 11–13 brown; antennal ratio 0.93–0.99 (0.96, n = 5). Palpus with segment 3 slightly swollen on midportion with broad, deep sensory pit; palpal ratio 2.00–2.33 (2.19, n = 5). **Thorax**. Scutum brown, darker laterally. Wing (Figure 11a) with three grayish areas: first near apex of M_1_, extending to wing margin in m_1_; second sigmoid-shaped, extending from m_1_ to wing margin in m_2;_ third over CuA1, CuA2 extending from mediocubital fork into cua_1_ and anal cell, reaching wing margin; 2nd radial cell twice length of 1st; length 1.02–1.37 (1.18, n = 6) mm; width 0.35–0.42 (0.39, n = 6) mm; costal ratio 0.69–0.73 (0.71, n = 6). Halter pale brown, distal portion of knob darker. Legs (Figure 11b) brown, hind leg darker, hind femur slightly darker subapically; hind tibial comb with 6 spines. Foretarsomere 1 with one basal, one apical spine; midtarsomere 1 with 3–5 ventral spines; apical spines of tarsomeres 2–4 of fore-, mid-, hind legs: 2-2-4, 2-2-4, 1-1-2; foretarsal ratio 2.15–2.29 (2.20, n = 6), midtarsal ratio 2.35–2.46 (2.41, n = 6), hind tarsal ratio 2.31–2.43 (2.39, n = 6); claws 0.40–0.47 (0.44, n = 6) length of their respective tarsomere 5. **Abdomen**. Brown. Terminalia (Figure 14a): tergite 9 with quadrate apex, apicolateral process very long, slender (Figure 14a); sternite 9 concave anteriorly, posterior margin with short, broad, convex median lobe bearing 3–6 long setae. Gonocoxite brown, moderately stout, 2.15–2.31 (2.25, n = 6) times longer than basal width; gonostylus brown, nearly straight, broad basally, 0.58–0.63 (0.60, n = 6) length of gonocoxite. Parameres (Figure 14c) 1.13–1.39 (1.30, n = 6) length of aedeagus, separated, each with basal arm trilobed, knob slender; stem sinuous, flattened apically; lateral sclerotized, slender, median process, directed posteromesally (Figure 14c). Aedeagus (Figure 14b) triangular, sclerotized, basal arch somewhat U-shaped, broad, extending to 0.35–0.40 (0.38, n = 6) of total length; distal portion with deep mesal notch and two heavily sclerotized pointed processes, with a membrane triangular processes in some specimens.

**Female**. Similar to male with usual sexual differences; antenna not visible in slide mounted specimens; palpal ratio 2.50–2.60 (2.55, n = 3) (Figure 11e); mandible with 9–11 teeth. Wing (Figure 11d) similar to male, except grayish area in M_1_ not extending to wing margin in m_1_; length 0.95–1.25 (1.11, n = 3) mm; width 0.37–0.47 (0.43, n = 3) mm; costal ratio 0.79–0.82 (0.80, n = 3). Foretarsomere 1 with one basal, one apical spine; midtarsomere 1 with 3–5 ventral spines; apical spines of tarsomeres 2–4 of fore-, mid legs: 2-2-3, 2-2-4; foretarsal ratio 2.35–2.36 (2.35, n = 3), midtarsal ratio 2.42–248 (2.45, n = 3), hind tarsal ratio 2.28–2.50 (2.31, n = 3); fore-, mid legs claws 0.56–0.61 (0.59, n = 3) length of their respective tarsomeres 5 (Figure 11g); hind leg claw 1.17–1.25 (1.21, n = 2) as long as tarsomere 5 (Figure 11g). Two subequal spermathecae (Figure 11f), measuring 50–60 (57, n = 3) by 45–52 (49, n = 3) µm and 50–57 (53, n = 3) by 45–50 (47, n = 2) µm. Third rudimentary spermatheca measuring 6.2 µm (not visible in Figure 11f).

**Specimens examined.** 1 male, on microscope slide, labeled “Holotype *Monohelea carioca* Tavares and Silva Pereira”, “Brazil, Rio de Janeiro, Tijuca, Gávea Pequena, February 1977, Tavares and Paiva cols.” (MNRJ); 3 males, 1 female, on microscope slide, labeled “Paratype *Monohelea carioca* Tavares and Silva Pereira”, same data as holotype (CCER); 2 females, on microscope slide, “Rio de Janeiro, Santa Cruz, December 1976, FEEMA cols.” (CCER); 2 males, on microscope slide, “Rio de Janeiro, Jacarepaguá, Pau da Fome, December 1975, Tavares col.” (CCER).

**Distribution and bionomics.** This species is known only from Brazil (Rio de Janeiro) in forested areas occurring from 20 to 300 m above sea level (Figure 10).

**Taxonomic discussion.** The male of *D. carioca* is distinctive within the genus, with male genitalia which has parameres separated, with sinuous stem flattened apically. Its coloration resembles those in *multilineata* species group, but *D. carioca* has a triangular aedeagus and paramere with a median process as in *D. fluminensis*. It can be separated from this last species by the number of ventral spines on midtarsomere 1 (3–5 in *D. carioca*; 1–2 in *D. fluminensis*), the median process of the paramere not beak-shaped and the absence of median horn-like process on the aedeagus.

#### 3.4.9. *Downeshelea castroi* (Tavares and Silva-Pereira)

**Figure 12:**
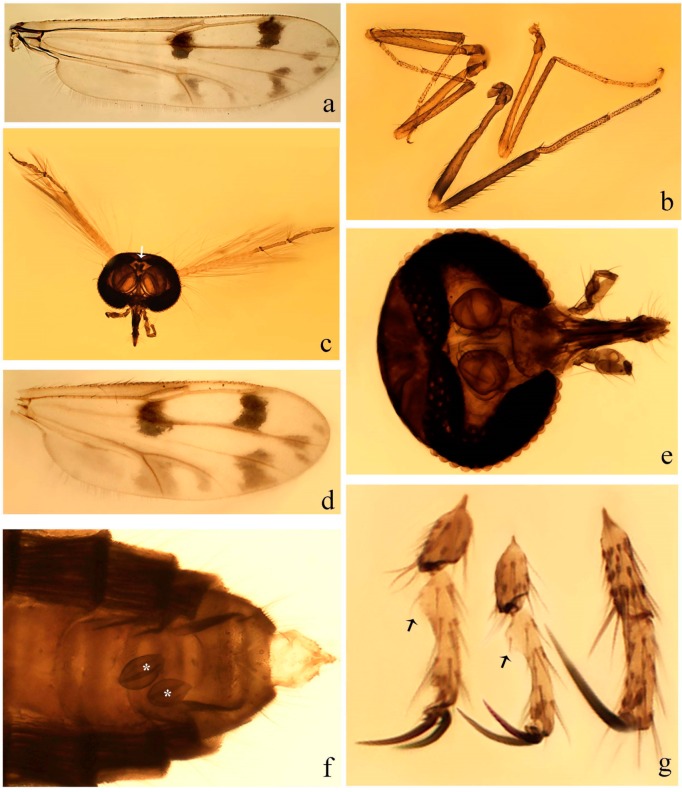
*Downeshelea castroi*, male: (**a**) wing; (**b**) fore-, mid-, hind legs (top to bottom), lateral view; (**c**) head, anterior view; arrow: eyes separation. Female: (**d**) wing; (**e**) head, anterior view; (**f**) apex of abdomen, ventral view; asterisks: spermathecae; (**g**) fore-, mid-, hind legs claws (left to right), lateral view; arrow: fore-, midtarsomere 5 swollen basoventrally.

Figure 12a–g, Figure 14d–f, and Figure 21; Table 1.

*Monohelea castroi* Tavares and Silva-Pereira, 1978 [35]: 159 (female, male, Brazil).

*Downeshelea castroi:* Wirth and Grogan, 1988 [7]: 51 (combination); Borkent and Wirth, 1997 [24]: 97 (in World catalog); Borkent and Spinelli, 2000 [25]: 47 (in catalog south of the USA; distribution); Borkent and Spinelli, 2007 [26]: 80 (in Neotropical catalog; distribution); Borkent, 2016 [22]: 124 (in World catalog); Santarém and Felippe-Bauer, 2019 [2]: 16 (Brazilian distribution).

**Diagnosis**. Male: only species of *Downeshelea* in the Americas with the following combination of character states: r_3_ with apical grayish spot (Figure 12a); legs brown, fore-, midtibia darker apically, hind femur darker subapically (Figure 12b); gonostylus short, broad (Figure 14d); paramere stem parallel (Figure 14f) with subbasal lateral lobe (Figure 14f); with two divergent sharp points distally, the inner one longer (Figure 14f); aedeagus Y-shaped (Figure 14e). Female: only species of *Downeshelea* in the Americas with the following combination of character states: wing (Figure 12d) and legs as described for male; hind leg claw 1.31 as long as tarsomere 5 (Figure 12g); unequal spermathecae (Figure 12f).

**Redescription**. **Male**. Head (Figure 12c). Eyes separated dorsomedially by 2.5× width of an ommatidium (Figure 12c). Antenna pale, except basal portion of flagellomere 1, distal portion of flagellomere 10, flagellomeres 11–13 brown; antennal ratio 1.01. Palpus with segment 3 slightly swollen on midportion with broad, deep sensory; palpal ratio 2.70. **Thorax.** Scutum brown, without definite pattern in slide mounted specimens. Wing (Figure 12a) with five grayish areas: two, small, round, in apical portion of cells r_3_, m_1,_ reaching wing margin; one near apex of M_1_, not reaching wing margin; one sigmoid-shaped, extending from m_1_ to wing margin in m_2;_ one over CuA, CuA_1,_ CuA_2_ extending into cua_1_ and anal cell, reaching wing margin; 2nd radial cell twice length of 1st; length 1.47 mm; width 0.45 mm; costal ratio 0.76. Halter brown. Legs (Figure 12b) brown, hind leg darker; fore-, midtibia slightly darker apically; hind femur darker subapically; hind tibial comb with seven spines. Foretarsomere 1 with one basal, one apical spine; midtarsomere 1 with four ventral spines; apical spines of tarsomeres 2–4 of fore-, mid-, hind legs: 2-2-4, 2-2-4, 1-1-2; foretarsal ratio 2.23, midtarsal ratio 2.25, hind tarsal ratio 2.28; claws 0.44 length of their respective tarsomere 5. **Abdomen.** Brown. Terminalia (Figure 14d): tergite 9 with quadrate apex, apicolateral process very long, slender; sternite 9 concave anteriorly, posterior margin with prominent convex median lobe bearing five long setae. Gonocoxite brown, moderately stout, 2.17 times longer than basal width; gonostylus brown, straight, broad, truncated tip, 0.55 length of gonocoxite. Parameres (Figure 14f) 1.11 length of aedeagus, fused on basal portion for 0.15 of total length, each with basal arm trilobed, knob bulbous; stem straight, parallel with subbasal lateral lobe (Figure 14f); with two divergent sharp points distally, the inner one longer (Figure 14f). Aedeagus (Figure 14e) triangular, Y-shaped, sclerotized, basal arch U-shaped, broad, extending to 0.45 of total length; distal portion with deep mesal notch and two sclerotized pointed serrate processes.

**Female**. Similar to male with usual sexual differences; antenna not visible in slide mounted specimen; palpus damaged (Figure 12e); mandible with 9 teeth. Wing as in Figure 12d, length 1.32 mm; width 0.50 mm; costal ratio 0.77. Foretarsomere 1 with one basal, one apical spine; midtarsomere 1 with eight ventral spines; apical spines of tarsomeres 2–4 of fore-, mid-legs: 2-2-4, 2-2-4. Foretarsal ratio 2.21, midtarsal ratio 2.40, hind tarsal ratio 2.59; fore-, mid leg claws 0.65 length of their respective tarsomeres 5; hind leg claw 1.31 as long as tarsomere 5 (Figure 12g). Two unequal spermathecae (Figure 12f), measuring 75 µm and 65 µm, width not measurable in slide mounted specimen. Third rudimentary spermatheca measuring 10 µm (not visible in Figure 12f).

**Specimens examined.** 1 male, on microscope slide, labeled “Holotype *Monohelea castroi* Tavares and Silva Pereira”, “Brazil, Rio de Janeiro, Tijuca, Gávea Pequena, February 1977, Tavares and Paiva cols.” (MNRJ); 1 female, on microscope slide, labeled “Paratype *Monohelea castroi* Tavares and Silva Pereira”, same data as holotype (CCER).

**Distribution and bionomics.** This species is known only from Brazil (Rio de Janeiro) in a forested area at 300 m above sea level (Figure 21).

**Taxonomic discussion.** Males of *D. castroi* most closely resembles those of *D. bifida*, *D. divergentis* and *D. quechua* in having distal portion of paramere bifid. It can be readily distinguished by the presence of a grayish spot in r_3_ and m_1_ (absent in the three aforementioned species). Other characters to distinguish them are in the taxonomic discussion of *D. bifida* and in the key.

#### 3.4.10. *Downeshelea cebacoi* (Lane and Wirth)

**Figure 13:**
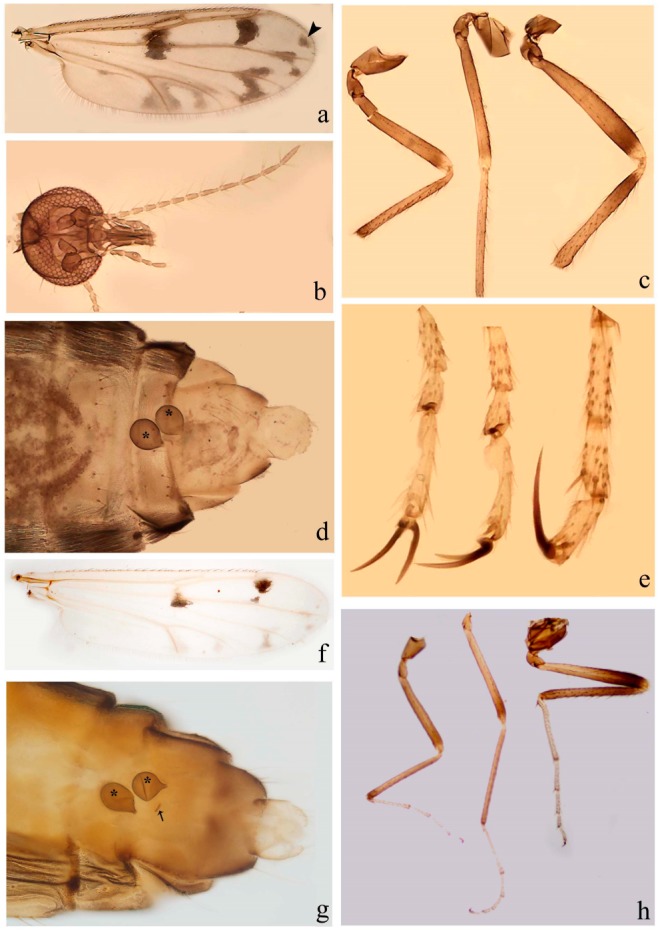
*Downeshelea cebacoi*, female: (**a**) wing; arrowhead: r_3_ apical grayish spot; (**b**) head, anterior view; (**c**) fore-, mid-, hind legs (left to right), lateral view; (**d**) apex of abdomen, ventral view; asterisks: spermathecae; (**e**) fore-, mid-, hind legs claws (left to right), lateral view. *Downeshelea charrua*, male: (**f**) wing; (**h**) fore-, mid-, hind legs (left to right), lateral view. Female: (**g**) apex of abdomen, ventral view; asterisks: spermathecae; arrow: 3rd rudimentary spermatheca.

**Figure 14:**
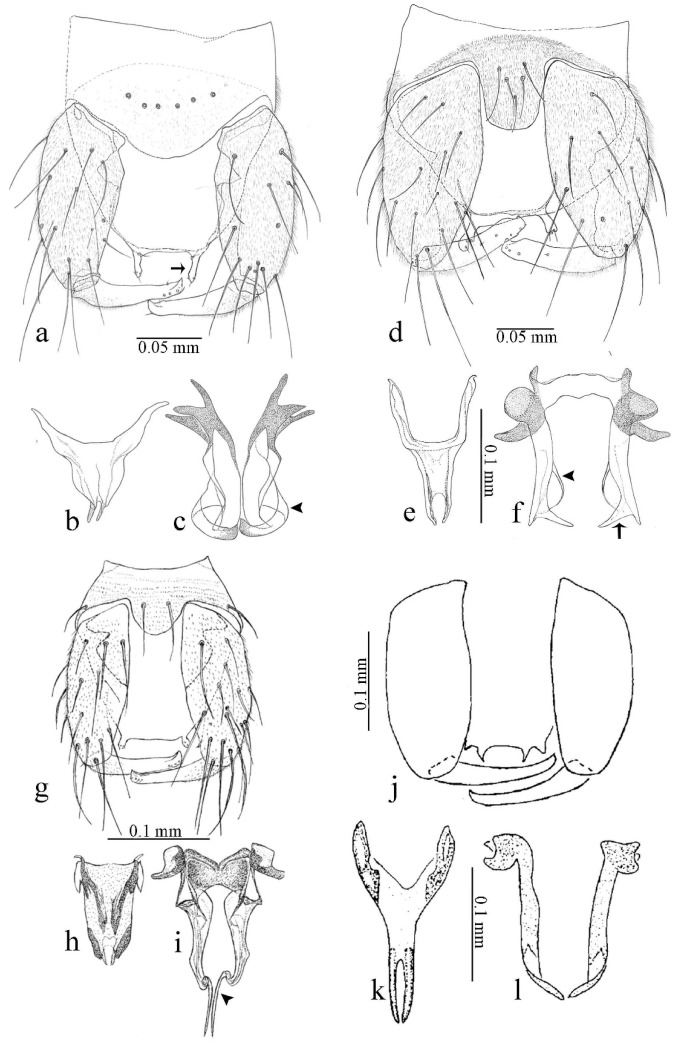
*Downeshelea carioca*, male: (**a**) terminalia, ventral view; arrow: tergite 9 apicolateral process; (**b**) aedeagus, ventral view; (**c**) parameres, ventral view; arrowhead: median process. *Downeshelea castroi*, male: (**d**) terminalia, ventral view; (**e**) aedeagus, ventral view; (**f**) parameres, ventral view; arrowhead: lateral lobe; arrow: inner distal point. *Downeshelea cebacoi*, male: (**g**) terminalia, ventral view; (**h**) aedeagus, ventral view; (**i**) parameres, ventral view; arrowhead: distal portion. *Downeshelea charrua*, male: (**j**) terminalia, ventral view; (**k**) aedeagus, ventral view; (**l**) parameres, ventral view.

Figure 13a–e, Figure 14g–I, and Figure 21; Table 1.

*Monohelea cebacoi* Lane and Wirth, 1964 [6]: 218 (male, female, Panama); Wirth, 1974 [3]: 40 (in catalog south of the USA; distribution).

*Downeshelea cebacoi:* Wirth and Grogan, 1988 [7]: 51 (combination); Borkent and Wirth, 1997 [24]: 97 (in World catalog); Borkent and Spinelli, 2000 [25]: 47 (in catalog south of the USA; distribution); Borkent and Spinelli, 2007 [26]: 80 (in Neotropical catalog; distribution); Felippe-Bauer et al., 2011 [20]: 25 (redescription; male; Costa Rica record); Borkent, 2016 [22]: 124 (in World catalog).

**Diagnosis.** Male: only species of *Downeshelea* in the Americas with the following combination of character states: r_3_ with apical grayish spot; legs pale brown, hind femur darker subapically, hind tibia darker subbasally (Figure 13c). Male paramere stem sinuous (Figure 14i); distal portion slender, long, directed posteriorly (Figure 14i); aedeagus subrectangular, basal arch very shallow (Figure 14h). Female: only species of *Downeshelea* in the Americas with the following combination of character states: wing (Figure 13a) and legs as described for male; hind leg claw 1.32 as long as tarsomere 5 (Figure 13e); equal-sized spermathecae (Figure 13d).

**Male.** Detailed redescription is found in Felippe-Bauer et al. [20].

**Redescription. Female.** Similar to male described by Felippe-Bauer et al. [28] with usual sexual differences; antenna brown, basal portion of flagellomeres 2–8 paler, antennal ratio 1.05–1.09 (1.07, n = 2); palpal ratio 2.17 (n = 2) (Figure 13b); mandible with 12 teeth. Wing as in Figure 13a; length 1.30–1.35 (1.32, n = 2) mm; width 0.52 (n = 2) mm; costal ratio 0.81–0.83 (0.82, n = 2). Foretarsomere 1 with one basal, two apical spines; midtarsomere 1 with 5–6 ventral spines; apical spines of tarsomeres 2–4 of fore-, mid-legs: 2-2-4, 2-2-4. Foretarsal ratio 2.48–2.57 (2.52, n = 2), midtarsal ratio 2.78–2.91 (2.85, n = 2), hind tarsal ratio 2.61–2.77 (2.69, n = 2); fore-, mid legs claws 0.67–0.74 (0.69, n = 2) length of their respective tarsomeres 5; hind leg claw 1.32 (n = 2) as long as tarsomere 5 (Figure 13e). Two equal-sized spermathecae (Figure 13d), measuring 58–63 (60, n = 2) µm by 45–48 (46, n = 2) µm. Third rudimentary spermatheca not visible.

**Specimens examined.** 1 male, in Canada Balsam on four celluloid strips on pin, labeled “Paratype *Monohelea cebacoi* Lane and Wirth”, “Panama, Bocas del Toro, Almirante, September 1952, F.S. Blanton col. (FSP); 2 males, 1 female, on microscope slide, labeled “Costa Rica, Limón, Est. Biol. Hitoy Cerere, Send. Toma de Agua, 100 m, 17 April–08 May 1999, Malaise Trap, F. Umana col (1 male, 1 female MNCR; 1 male CNCI); 1 female on microscope slide, same data except “14–16 April 1999, G. Chaverri, E Rojas and B. Hernández cols. (MNCR); 1 male, on microscope slide, same data except “Valle del Silencio, Sendero Espavel, 220 m, 17 February–17 March 2000” (CCER); 1 male, on microscope slide, labeled “Puntarenas, Buenos Aires, Estación Altamira, Sendero Los Gigantes, 1450 m, 3–22 February 2000, Amarilla, D. Rubi col. (MNCR); 1 male, on microscope slide, labeled “Golfito, P.N. Corcovado, Estación Los Patos, Send. a Sirena, 70 m, 25 December 2000–13 February 2001, Malaise Trap, J. Azofeifa col. (MNCR); 1 male, on microscope slide, labeled “Cartago, P.N. Barbilla, Send Principal a Rio Barbilla, 400–500 m, 19 August 2001, Red de Golpe, E. Rojas and F. Umana cols. (MNCR); 3 females, on microscope slide, same data except “2 km S. Rio Barbilla, Camin. a Moravia, 800 m, 27 October 2000, malaise, E. Rojas col. (MNCR).

**Distribution.** This species is known from coastal and forested humid areas in Costa Rica (Puntarenas, Cartago and Limón) and Panama. It occurs from 15 m in Panamá to 1450 m above sea level in Costa Rica (Figure 21).

**Remarks.** The male holotype of *D. cebacoi* is a pinned specimen deposited in the USNM (#66437). It was studied by Felippe-Bauer et al. [20] when the authors redescribed the male of this species and described *D. lanei* Felippe-Bauer and Borkent, based on a male paratype of *Monohelea cebacoi* Lane and Wirth from Pará and other male and female specimens from the same state of Brazil.

#### 3.4.11. *Downeshelea charrua* Felippe-Bauer and Spinelli

Figure 13f–h, Figure 14j–l, and Figure 21; Table 1.

*Downeshelea charrua* Felippe-Bauer and Spinelli, 1994 [17]: 161 (male, female; Uruguay); Borkent and Wirth, 1997 [24]: 97 (in World catalog); Borkent and Spinelli, 2000 [25]: 47 (in catalog south of the USA; distribution); Borkent and Spinelli, 2007 [26]: 80 (in Neotropical catalog; distribution); Borkent, 2016 [22]: 124 (in World catalog).

**Diagnosis.** Male: only species of *Downeshelea* in the Americas with the following combination of character states: r_3_ with apical grayish spot (Figure 13f); legs pale brown, mid-, hind femur darker subapically, hind tibia darker subbasally (Figure 13h), hind tibial comb with 8 spines; male parameres separated, straight, mesally curved apically (Figure 14l); aedeagus Y-shaped, distal portion with blunt apex (Figure 14k). Female: only species of *Downeshelea* in the Americas with the following combination of character states: wing and legs as described for male; hind leg claw 1.40 as long as tarsomere 5; unequal spermathecae (Figure 13g); 3rd rudimentary spermatheca long (Figure 13g).

**Specimens examined.** 1 male, on microscope slide, labeled “Holotype *Downeshelea charrua* Felippe-Bauer and Spinelli”, “Uruguay, Salto, El Espinillar, 24 April 1985, G. Spinelli” (MLP); 1 female, on microscope slide, labeled “Allotype *Downeshelea charrua* Felippe-Bauer and Spinelli” same data (MLP).

**Distribution.** This species is known only from Uruguay at 50 m above sea level (Figure 21).

#### 3.4.12. *Downeshelea chiapasi* (Lane and Wirth)

**Figure 15:**
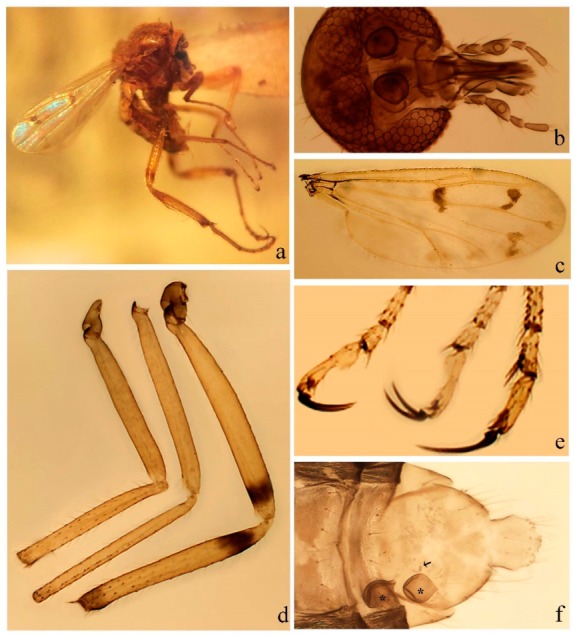
*Downeshelea chiapasi*, male: (**a**) paratype pinned specimen. Female: (**b**) head, anterior view; (**c**) wing; (**d**) fore-, mid-, hind legs (left to right), lateral view; (**e**) fore-, mid-, hind legs claws (left to right), lateral view; (**f**) apex of abdomen, ventral view; asterisks: spermathecae; arrow: 3rd rudimentary spermatheca.

Figure 15a–f, Figure 19a–c, and Figure 21; Table 1.

*Monohelea chiapasi* Lane and Wirth, 1964 [6]: 219 (male, female, Nicaragua); Wirth, 1974 [3]: 41 (in catalog south of the USA; distribution).

*Downeshelea chiapasi*: Wirth and Grogan, 1988 [7]: 51 (combination); Borkent and Wirth, 1997 [24]: 97 (in World catalog); Borkent and Spinelli, 2000 [25]: 47 (in catalog south of the USA; distribution); Borkent and Spinelli, 2007 [26]: 80 (in Neotropical catalog; distribution); Borkent, 2016 [22]: 124 (in World catalog).

**Diagnosis**. Male: only species of *Downeshelea* in the Americas with the following combination of character states: r_3_ without apical grayish spot; legs yellowish brown, hind femur with subapical, hind tibia with subbasal, apical dark band (Figure 15a); gonostylus yellowish, distal ½ darker; parameres (Figure 19c) separated, stem with subapical processes anteriorly directed with dorsal membrane expansion (Figure 19c); aedeagus rectangular (Figure 19b). Female: only species of *Downeshelea* in the Americas with the following combination of character states: wing (Figure 15c) and legs (Figure 15d) as described for male; hind leg claw 1.35–1.46 as long as tarsomere 5 (Figure 15e); equal-sized spermathecae (Figure 15f).

**Redescription. Male** (Figure 15a)**. Thorax**. Yellowish brown; scutum with inconspicuous brown spots. Wing (as female in Figure 15c) with four grayish areas: first, small, near apex of M_1_ (absent in some specimens); second L-shaped, extending from m_1_ to wing margin in m_2_; third, small, in cua_1_ near CuA_1_, not reaching wing margin; fourth in distal ½ of CuA_2_ extending into cua_1,_ anal cell, not reaching wing margin. Halter knob darkened. Legs (as female in Figure 15d) yellowish brown, hind femur with subapical, hind tibia with subbasal, apical dark band. **Abdomen**. Yellowish with ventrolateral brown marks on segments 1–7. Terminalia (Figure 19a): tergite 9 with quadrate apex, apicolateral process short; sternite 9 slightly concave anteriorly, posterior margin with moderately convex median lobe bearing 3–4 long setae. Gonocoxite brown, moderately stout, 2.00 (n = 3) times longer than basal width; gonostylus yellowish, distal ½ darker, nearly straight, flattened tip, 0.64–0.70 (0.67, n = 3) length of gonocoxite. Parameres (Figure 19c) 1.15–1.29 (1.23, n = 3) length of aedeagus, separated, each with basal arm trilobed, knob bulbous; stem nearly straight, slightly expanded apically with subapical, slender, sclerotized processes bent, directed anteriorly with dorsal membrane expansion (Figure 19c), 0.45–0.54 (0.50, n = 3) of total length. Aedeagus (Figure 19b) rectangular, basal arch somewhat U-shaped, sclerotized, extending to 0.29–0.31 (0.30, n = 2) of total length; distal portion with deep mesal notch and two rounded pointed processes.

**Female.** Similar to male with usual sexual differences; antenna not visible in slide mounted specimens; palpus with segment 3 slightly swollen on midportion with broad, deep sensory pit; palpal ratio 2.10–2.50 (2.30, n = 2) (Figure 15b); mandible with 10 teeth. Wing as in Figure 15c; length 1.26–1.45 (1.35, n = 3) mm; width 0.60–0.65 (0.62, n = 2) mm; costal ratio 0.85–0.86 (0.86, n = 2). Foretarsomere 1 with one basal, one apical spine; midtarsomere 1 without ventral spines; apical spines of tarsomeres 2–4 of fore-, mid legs: 2-2-4, 2-2-4; foretarsal ratio 2.29–2.44 (2.37, n = 2), midtarsal ratio 2.43–2.73 (2.58, n = 2), hind tarsal ratio 2.22–2.76 (2.49, n = 2); fore-, mid legs claws 0.78–0.83 (0.81, n = 2) length of their respective tarsomeres 5; hind leg claw 1.35–1.46 (1.41, n = 2) as long as tarsomere 5 (Figure 15e). Two equal-sized spermathecae (Figure 15f), measuring 65–67 (66, n = 2) by 45–55 (50, n = 2) µm. Third rudimentary spermatheca measuring 7.5 µm (Figure 15f).

**Specimens examined.** 1 male, pinned, with genitalia in drop of Canada Balsam on celluloid strip on pin, labeled “Paratype, *Monohelea chiapasi* Lane and Wirth, drawn”, “Panama, Mojinga Swamp, Ft Sherman, Canal Zone, 17 February 1952, light trap, FS Blanton col.” (FSP); 1 female, pinned, labeled “Paratype, *Monohelea chiapasi* Lane and Wirth”, same data except “November 1951” (FSP); 1 female, pinned, labeled “Paratype, *Monohelea chiapasi* Lane and Wirth”, same data except “Mindi Dairy, 22 August 1952” (FSP); 1 male, pinned, with genitalia in drop of Canada Balsam on celluloid strip on pin, labeled “Paratype, *Monohelea chiapasi* Lane and Wirth”, same data except “Juan Diaz, 24 April 1952” (FSP); 1 male, pinned, with genitalia in drop of Canada Balsam on celluloid strip on pin, labeled “Paratype, *Monohelea chiapasi* Lane and Wirth”, “NICARAGUA, Villa Somoza (actually Villa Sandino), July 1953, P Galindo col.” (FSP); 1 female, on microscope slide, labeled “Costa Rica, Puntarenas, Osa, Ciudad Porto Cortês, Finca A y A. Patos, 100–200 m, 14–19 August 2005, malaise, Moraga col.” (CCER); 1 female, on microscope slide, labeled “Puntarenas, Garabito, PN Carara, Sector Languna Meandrica, Sitio Quebrada Mona, 100 m, May–July 1990, manual, R. Zuñiga col.” (MNCR) (NEW RECORD).

**Distribution and bionomics.** This species is known from Nicaragua, Costa Rica (Puntarenas), and Panama (Figure 21). It has been found in coastal and humid forested areas, occurring at sea level in Panama, from 100 to 200 m in Costa Rica and in Nicaragua at 300 m above sea level.

**Taxonomic discussion.** We have studied five paratypes from Nicaragua and Panama indicated by Lane and Wirth [6] as deposited in FSP. Some data on the specimen labels did not match that cited in the original description and which is corrected here. The male holotype and female allotype from Nicaragua are pinned specimens (#66348 USNM) and no further observations were made of them. One male paratype from Colombia (IV.1963) was studied and is here described as the new species *D. spatha*. Other specimens from Colombia, labeled by Wirth as *D. chiapasi*, are also misidentified and belong to the new species *D. gladius* and *D. spatha*. As such, *D. chiapasi* is no longer recorded from Colombia. *Downeshelea chiapasi* is easily distinguished from other similar species by the strong dark bands on the hind leg (weak in *D. gladius*) and the absence of basal dark band on the hind femur (present in *D. spatha*). *Downeshelea chiapasi* also has wing and legs pattern similar *to D. avizi* and *D. colombiae*. Characters for distinguishing these five related species are in the discussion section of *D. avizi.*

#### 3.4.13. *Downeshelea chirusi* (Lane and Wirth)

**Figure 16:**
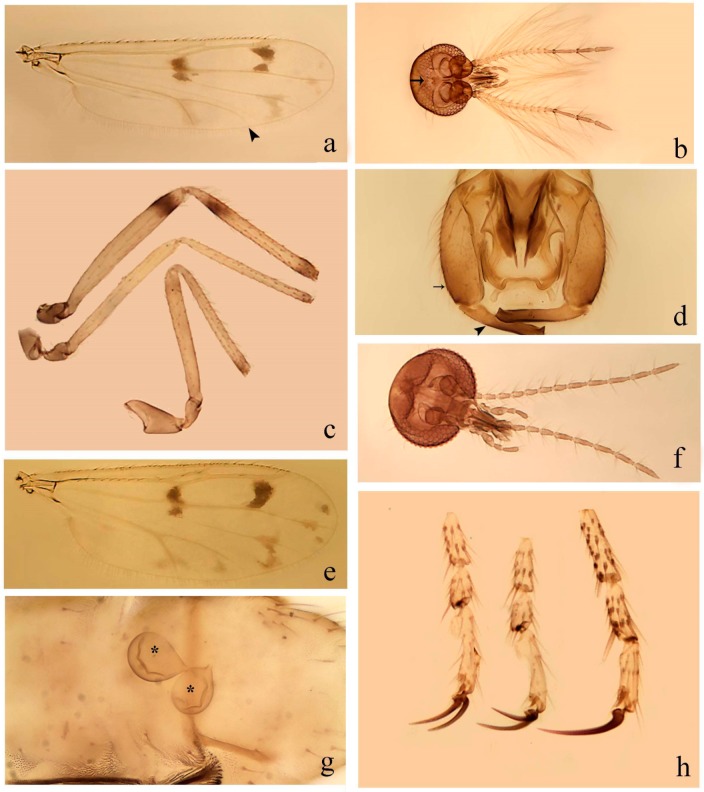
*Downeshelea chirusi*, male: (**a**) wing, arrowhead: CuA_1_; (**b**) head, anterior view; arrow: eyes separation; (**c**) fore-, mid-, hind legs (bottom to top), lateral view; (**d**) terminalia, ventral view; arrow: gonocoxite; arrowhead: gonostylus. Female: (**e**) wing; (**f**) head, anterior view; (**g**) apex of abdomen, ventral view; asterisks: spermathecae; (**h**) fore-, mid-, hind legs claws (left to right), lateral view.

Figure 16a–h, Figure 19d,e, and Figure 21 Table 1.

*Monohelea chirusi* Lane and Wirth, 1964 [6]: 218 (male, female; Panama specimens (in part), Nicaragua); Wirth, 1974 [3]: 40 (in catalog south of the USA; distribution).

*Downeshelea chirusi*: Wirth and Grogan, 1988 [7]: 51 (combination). Borkent and Wirth, 1997 [24]: 97 (in World catalog); Borkent and Spinelli, 2000 [25]: 47 (in catalog south of the USA; distribution); Borkent and Spinelli, 2007 [26]: 80 (in Neotropical catalog; distribution); Borkent, 2016 [22]: 124 (in World catalog).

**Diagnosis**. Male: only species of *Downeshelea* in the Americas with the following combination of character states: r_3_ with apical grayish spot (Figure 16a); legs yellowish brown, hind femur with subapical, hind tibia with subbasal, apical dark band (Figure 16c); gonocoxite (Figure 16d) and gonostylus (Figure 16d) yellowish brown at basal ½, distal ½ dark brown; paramere stem tapering posteriorly, expanded in median inner portion (Figure 19e); distal portion slender curved mesad, bent, directed mesally (Figure 19e). Female: only species of *Downeshelea* in the Americas with the following combination of character states: wing (Figure 16e) and legs as described for male; hind leg claw 1.06–1.30 as long as tarsomere 5 (Figure 16h); slightly unequal pale spermathecae (Figure 16g).

**Redescription. Male**. **Head** (Figure 16b). Eyes separated dorsomedially by 2× width of an ommatidium (Figure 16b). Antenna pale, except base of flagellomere 1, distal portion of flagellomere 10 and flagellomeres 11–13 brown; antennal ratio 1.00–1.11 (1.02, n = 16). Palpus with segment 3 slightly swollen on midportion with broad, deep sensory pit; palpal ratio 2.00–2.40 (2.26, n = 17). **Thorax**. Scutum yellowish, with brown dots over the whole disc; pleura yellowish. Wing (Figure 16a) with six grayish areas: three, small, round, in apical portion of cells r_3_, m_1,_ on vein M_1_, arranged in a triangle, not reaching wing margin; one sigmoid-shaped extending from m_1_ to wing margin in m_2_; one round, small, in cua_1_ near CuA_1_ not reaching wing margin (as female in Figure 16e), absent in some specimens; one over CuA_2_ extending into cua_1_, anal cell, reaching wing margin; 2nd radial cell twice length of 1st; length 1.00–1.27 (1.13, n = 17) mm, width 0.35–0.42 (0.38, n = 17) mm; costal ratio 0.73–0.80 (0.78, n = 17). Halter pale, distal portion of knob darker. Legs (Figure 16c) yellowish brown, hind femur with subapical, hind tibia with subbasal, apical dark band; hind tibial comb with 6–7 spines. Foretarsomere 1 with one basal, one apical spine; midtarsomere 1 with 3–4 ventral spines; apical spines of tarsomeres 2–4 of fore-, mid-, hind legs: 2-2-2, 2-2-2, 1-1-2, foretarsal ratio 2.25–2.53 (2.40, n = 17), midtarsal ratio 2.20–2.76 (2.50, n = 17), hind tarsal ratio 2.10–2.45 (2.27, n = 17); claws 0.35–0.50 (0.42, n = 17) length of their respective tarsomere 5. **Abdomen**. Yellowish. Terminalia (Figure 16d and Figure 19d): tergite 9 with quadrate apex, apicolateral process long, slender; sternite 9 straight anteriorly, posterior margin with large convex median lobe bearing two long setae. Gonocoxite yellowish brown, darkening apically (Figure 16d), moderately stout, 2.27–2.64 (2.46, n = 16) times longer than basal width; gonostylus light brown at basal ½, distal ½ dark brown (Figure 16d), straight with a distinct pointed tip, 0.58–0.70 (0.65, n = 16) length of gonocoxite. Parameres (Figure 19e) 0.96–1.18 (1.06, n = 17) length of aedeagus, fused on basal portion for 0.09–0.10 (0.09, n = 6) of total length, each with trilobed basal arm; knob bulbous; stem sinuous, tapering posteriorly, expanded in median inner portion; distal portion (Figure 19e) short, slender, curved mesad, bent directed mesally, tapering to pointed tip, 0.27–0.41 (0.33, n = 17) of total length. Aedeagus (Figure 19d) rectangular, sclerotized, with pair of admedian, heavily sclerotized processes extending from basal arch to distal portion (Figure 19d), basal arch U-shaped, extending to 0.39–0.53 (0.48, n = 15) of total length; distal portion with deep mesal notch and two slender, heavily sclerotized, pointed slightly serrate processes.

**Female**. Similar to male with usual sexual differences; antenna brown, basal portion of flagellomeres pale; antennal ratio 1.00–1.11 (1.06, n = 15); palpal ratio 2.00–2.40 (2.26, n = 17) (Figure 16f); mandible with 11 teeth. Wing as in Figure 16e; length 1.12–1.47 (1.26, n = 17) mm; width 0.45–0.62 (0.51, n = 17) mm; costal ratio 0.80–0.85 (0.82, n = 17). Foretarsomere 1 with one basal (two in some specimens), two apical spines; midtarsomere 1 with 3–4 ventral spines; apical spines of tarsomeres 2–4 of fore-, mid legs: 3-3-2 (2-2-2 in some specimens); 2-2-2; foretarsal ratio 2.35–2.64 (2.48, n = 17), midtarsal ratio 2.50–2.86 (2.66, n = 17), hind tarsal ratio 2.37–2.59 (2.51, n = 17); fore-, mid legs claws 0.67–0.80 (0.72, n = 17) length of their respective tarsomeres 5; hind leg claw 1.06–1.30 (1.22, n = 16) as long as tarsomere 5 (Figure 16h). Two slightly unequal pale spermathecae (Figure 16g), measuring 48–70 (57, n = 17) by 38–50 (46, n = 14) µm and 40–73 (51, n = 17) by 35–45 (40, n = 12) µm. Third rudimentary spermatheca measuring 8.7 µm (not visible in Figure 16g).

**Specimens examined.** Male, pinned, with genitalia in drop of Canada Balsam on celluloid strip on pin, labeled “Holotype *Monohelea chirusi* Lane and Wirth”, “Panama, Coclé Prov., Puerto Obaldia, 11 November 1952, light trap, F.S. Blanton col.(USNM)”;1 female, pinned, labeled “Paratype *Monohelea chirusi* Lane and Wirth”, same data as holotype (FSP); 1 male, pinned, with genitalia in drop of Canada Balsam on celluloid strip on pin, labeled “Paratype *Monohelea chirusi* Lane and Wirth”, “Panama, Bocas del Toro, Almirante, 28 October 1952, light trap, F.S. Blanton col.”(FSP); 1 female, on microscope slide, labeled “Costa Rica, Alajuela, Upala, Parque Nacional Guanacaste, Sector San Ramon, 4.75 km SW dos rios de Upala, 860 m, Malaise, 17 May–17 June 1996, D. Briceno col.” (MNCR) (NEW RECORD); 1 male, on microscope slide, labeled “Alajuela, San Carlos, Pital, Boca tapada, Bosque ancianos, 50–100 m, 23 July–17 April 2004, Malaise, B. Hernández col.” (MNCR); 4 males, 5 females same data except “Finca de Sergio Murillo, 21 July 2004, light trap” (1 male, 1 female CCER; 1 males, 2 females MNCR; 2 males, 2 females CNCI); 1 female, on microscope slide, labeled “Cartago, Parque Nacional Barbilla, Sendero principal a Rio Barbilla, 500 m, 12 June–11 July 2002, Malaise, E. Rojas col.” (MNCR); 1 female, on microscope slide, labeled “Heredia, Refugio Vida Silvestre Corredor Fronterizo Costa Rica Nicarágua, Lagunas a la par de rio San Juan, 20–50 m, 16 April 2004, light trap, B. Hernández col.” (MNCR); 3 females same data except “Malaise” (1 CCER; 2 MNCR); 2 males same data except “23 July 2004” (MNCR); 1 male, 1 female, on microscope slide, labeled “Limón, Pococi, Parque Nacional Braulio Carrillo, Estación Quebrada González, 400–500 m, 4 July 2002, Malaise, P. Hanson and C. Godoy cols.” (MNCR); 1 female, on microscope slide, labeled “Limón, Parque Nacional Tortuguero, Estación Agua fria, sendero real, 20–50 m, 16 August 2004, red noyes, M. Moraga col.” (CNCI); 1 male same data except “14–21 August 2004, Malaise, Porras, Gamboa, Briceno, Moraga and Cardenas cols.” (MNCR); 1 male, 2 females, on microscope slide, labeled “Limón, Estación Biológica Hitoy Cerere, Sendero Toma de Agua, 100 m, 17 April–8 May 1999, Malaise, F. Umana col.” (1 female CCER, 1 male, 1 female MNCR); 3 females same data except “560 m, 12 July 1998, E. Rojas col.” (MNCR); 1 male, 1 female, on microscope slide, labeled “Puntarenas, A.C.O. Golfito, Reserva Florestal Golfo Dulce, Estación Agujas, 250–350 m, 4–22 May 1999, red de golpe, J. Azofeifa col.” (CNCI); 1 male same data except “375 m, 16 April–16 May 1999, Malaise” (MNCR); 4 females, on microscope slide, labeled “Puntarenas, Golfito, Parque Nacional Corcovado, Estación Agujas, La Bonanza, 495 m, 15 May–15 June 1999, Malaise, J. Azofeifa col.” (MNCR); 6 females same data except “17 April–16 May 1999” (MNCR); 1 female same data except “Charcos, 600 m” (MNCR); 1 male same data except “Estacion Naranjales, 24 April 2004, light trap, Porras, Gamboa, Briceno and Moraga cols.” (MNCR); 1 male, 2 females same data except “Camino a Torres, 400–500 m, 23 April 2004” (MNCR); 1 female, on microscope slide, labeled “Puntarenas, Garabito, Parque Nacional Carara, Sector Laguna Meandríca, Sítio Quebrada Mona, 100 m, May–June 1990, manual, R. Zuniga col.” (MNCR); 1 male, on microscope slide, labeled “San José, Area de Conservación la Amistad Pacífico, Pérez Zeledón, San Pedro, Estación Santa Elena, Las Nubes, 1210 m, 16–31 March 1996, manual red libre, E. Alfaro and M. Segura cols.” (MNCR); 1 male adult, on microscope slide, labeled “San José, Parque Nacional Braulio Carrillo, Sítio La Montura, 1100 m, 10–13 June 2007, Malaise, A. Garcia, M. Moraga and M. Zumbado cols.”(CCER); 4 males, 1 female labeled “Mexico, Veracruz, Cuitlahuac, 10 August 1964, light trap, PJ Spangler col.” (NEW RECORD) (1 male FSP; 1 male CCER; 2 males, 1 female USNM).

**Distribution and bionomics.** This species is known from Mexico (Veracruz), Nicaragua (?), Costa Rica (Alajuela, Heredia, Puntarenas, San José, Cartago and Limón) and Panamá in coastal and humid forested areas (Figure 21). It has been found at sea level in Panama, from 20 to 1210 m in Costa Rica and from 300 to 400 m above sea level in Mexico and Nicaragua.

**Taxonomic discussion.** The analysis of the type series deposited in the FSP and USNM collections showed the presence of pale and dark morphotypes. The holotype of *D. chirusi* (#66439 USNM) is represented by the pale form and the allotype (USNM) by the dark one that we describe below as *D. pulla* sp. nov. Both species are also found in male and female paratypes from Panama. Those from Nicaragua (1 male, 1 female) were not located in any of the studied collections but have been described as yellowish, suggesting they may indeed be *D. chirusi*. We present further comments in the taxonomic discussion of *D. pulla* sp. nov. below.

#### 3.4.14. *Downeshelea colombiae* (Lane and Wirth)

**Figure 17:**
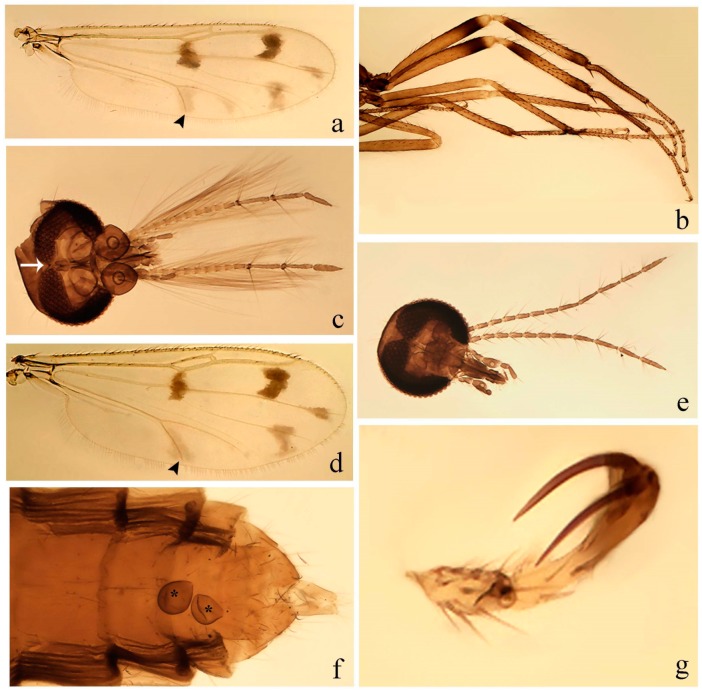
*Downeshelea colombiae*, male: (**a**) wing, arrowhead: CuA_2_; (**b**) fore-, mid-, hind legs (bottom to top), lateral view; (**c**) head, anterior view; arrow: eyes separation. Female: (**d**) wing, arrowhead: CuA_2_; (**e**) head, anterior view; (**f**) apex of abdomen; asterisks: spermathecae; (**g**) fore leg claws, ventral view.

Figure 17a–g, Figure 19f,g, and Figure 21; Table 1.

*Monohelea colombiae* Lane and Wirth, 1964 [6]: 220 (male; Colombia); Wirth, 1974 [3]: 40 (in catalog south of the USA; distribution).

*Downeshelea colombiae:* Wirth and Grogan, 1988 [7]: 51 (combination); Borkent and Wirth, 1997 [24]: 98 (in World catalog); Borkent and Spinelli, 2000 [25]: 47 (in catalog south of the USA; distribution); Borkent and Spinelli, 2007 [26]: 80 (in Neotropical catalog; distribution); Borkent, 2016 [22]: 124 (in World catalog).

**Diagnosis**. Male: only species of *Downeshelea* in the Americas with the following combination of character states: r_3_ without apical grayish spot (Figure 17a); legs yellowish brown, fore-, midtibia darker apically, hind femur with subapical, hind tibia with subbasal, apical dark band (Figure 17b); parameres broadly fused on basal portion (Figure 19g); subapical process long, deeply curved (Figure 19g); aedeagus subrectangular (Figure 19f). Female: only species of *Downeshelea* in the Americas with the following combination of character states: wing (Figure 17d) and legs as described for male; slightly unequal spermathecae (Figure 17f).

**Redescription. Male**. **Head** (Figure 17c). Eyes narrowly contiguous in lower portion (Figure 17c). Antenna pale, except basal portion of flagellomere 1, distal portion of flagellomere 10, flagellomeres 11–13 brown; antennal ratio 0.99–1.05 (1.02, n = 9). Palpus with segment 3 slightly swollen on midportion with broad, deep sensory pit; palpal ratio 2.14–2.60 (2.30, n = 8). **Thorax**. Scutum yellowish brown, without definite pattern in slide mounted specimens; pleura pale brown. Wing (Figure 17a) with three grayish areas: first near apex of M_1_ not abutting wing margin; second extending from m_1_ to wing margin in m_2_; third over CuA_1_, CuA_2_ extending into cua_1_, anal cell, reaching wing margin (Figure 19a,d); 2nd radial cell twice length of 1st; length 1.07–1.25 (1.17, n = 9) mm; width 0.35–0.42 (0.38, n = 9) mm; costal ratio 0.76–0.80 (0.77, n = 9). Halter pale brown, distal portion of knob darker. Legs (Figure 17b) yellowish brown, fore-, midtibia slightly darker apically, hind femur with subapical, hind tibia with subbasal, apical dark band; hind tibial comb with 6–7 spines. Foretarsomere 1 with one basal, one apical spine; midtarsomere 1 with 0–2 ventral spines; apical spines of tarsomeres 2–4 of fore-, mid-, hind legs: 2-2-4, 2-2-4, 1-1-2; foretarsal ratio 2.30–2.52 (2.39, n = 8), midtarsal ratio 2.40–2.68 (2.55 n = 9), hind tarsal ratio 2.21–2.35 (2.26, n = 9); claws 0.40–0.50 (0.45, n = 9) length of their respective tarsomere 5. **Abdomen**. Yellowish, with ventrolateral brown marks on segments 1–7. Terminalia brown (Figure 19f): tergite 9 with quadrate apex, apicolateral process long; sternite 9 concave anteriorly, posterior margin with prominent convex median lobe bearing two long setae. Gonocoxite brown, moderately stout, 2.03–2.33 (2.21, n = 9) times longer than basal width; gonostylus brown, curved, 0.67–0.75 (0.71, n = 9) length of gonocoxite. Parameres (Figure 19g) 0.91–0.98 (0.95, n = 9) length of aedeagus, fused on basal portion for 0.40–0.44 (0.43, n = 9) of total length, each with basal arm trilobed, knob bulbous; stem straight, expanded distally; with subapical, slender process abruptly bent, deeply curved, directed anteromesally, overlapped in some specimens (Figure 19g), 0.67–0.75 (0.71, n = 9) of total length. Aedeagus (Figure 19f) subrectangular, sclerotized laterally, basal arch V-shaped, stout, heavily sclerotized, extending to 0.38–0.45 (0.42, n = 9) of total length; distal portion with deep mesal notch and two slightly serrate pointed processes.

**Female**. Similar to male with usual sexual differences; antenna pale brown; basal portion of flagellomeres slightly pale; antennal ratio 1.05–1.09 (1.07, n = 2); palpal ratio 2.14–2.29 (2.21, n = 2) (Figure 17e); mandible with 9–10 teeth. Wing as in Figure 17d; length 1.32–1.40 (1.36, n = 2) mm; width 0.52–0.57 (0.55, n = 2) mm; costal ratio 0.83–0.84 (0.83, n = 2). Foretarsomere 1 with one basal, one apical spine; midtarsomere 1 with 1–2 ventral spines; apical spines of tarsomeres 2–4 of fore-, mid legs: 2-2-4, 2-2-4; foretarsal ratio 2.27–2.50 (2.39, n = 2), midtarsal ratio 2.68–2.70 (2.69, n = 2), hind tarsal ratio 2.45–2.63 (2.54, n = 2); fore-, mid legs claws 0.73–0.80 (0.76, n = 2) length of their respective tarsomeres 5 (Figure 17g); hind leg claw not measurable in slide mounted specimens. Two slightly unequal spermathecae (Figure 17f), measuring 55–70 (62, n = 2) by 45–57 (51, n = 2) µm and 52–62 (57, n = 2) by 50 (n = 2) µm. Third rudimentary spermatheca measuring 7.5 µm (not visible in Figure 17f).

**Specimens examined.** 1 male, on microscope slide, labeled “Colombia, Valle Rio Raposo, 1 July 1964, light trap, V.H. Lee col.” (USNM); 2 males, on microscope slide, same data except “28 July 1964”; 3 males, 1 female, on microscope slide, same data except August 1964” (1 male, 1 female CCER; 2 males USNM); 6 males, 1 female, on microscope slide, same data except “V.1965” (1 male, 1 female); “June 1965” (1 male), “July 1965” (1 male), August 1965” (3 males) (USNM).

**Distribution and bionomics.** This species is known only from Colombia (Figure 21). It has been found in mangrove areas.

**Taxonomic discussion.***Downeshelea colombiae* is similar to *D. avizi*, *D. chiapasi*, *D. gladius* and *D. spatha* in having a pattern of grayish spots on the wing and legs with dark bands. Characters for distinguishing these species are in the discussion section of *D. avizi*. We could not study the holotype of *D. colombiae* (#67563 USNM), which is a slide mounted male specimen. However, we have studied here some male and female specimens from the type locality, designated as *D. colombiae* by Wirth, that match its description. Based on this material, we redescribe the male and describe the females for the first time.

#### 3.4.15. *Downeshelea curta* sp. nov.

**Figure 18:**
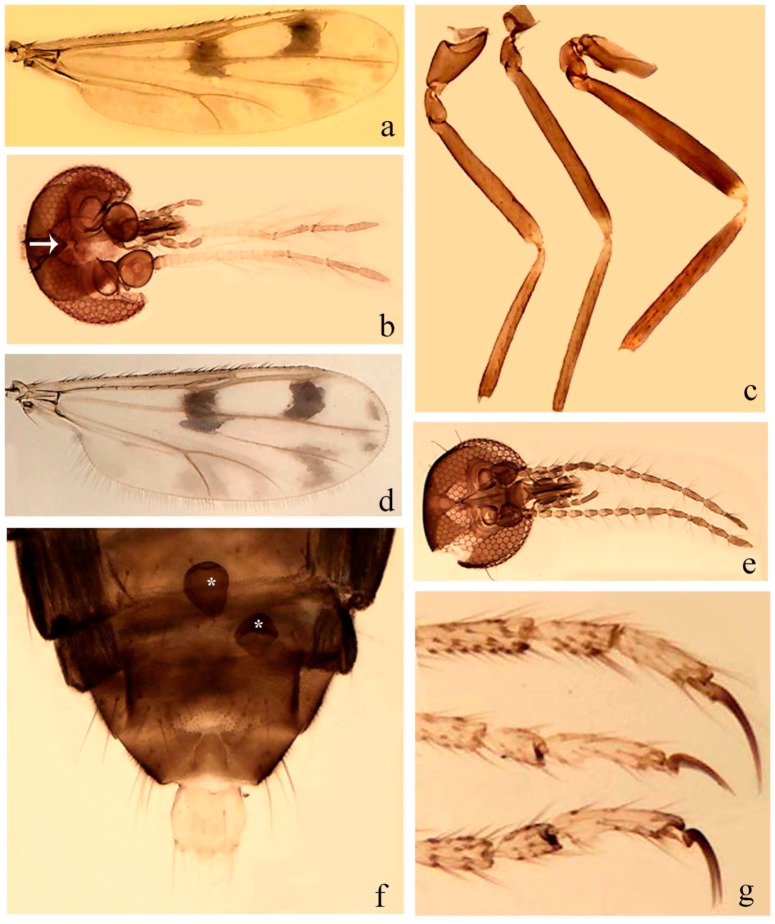
*Downeshelea curta* sp. nov., male: (**a**) wing; (**b**) head, anterior view; arrow: eyes separation; (**c**) fore-, mid-, hind legs (left to right), lateral view. Female: (**d**) wing; (**e**) head, anterior view; (**f**) apex of abdomen; asterisks: spermathecae; (**g**) fore-, mid-, hind legs claws (bottom to top), lateral view.

**Figure 19:**
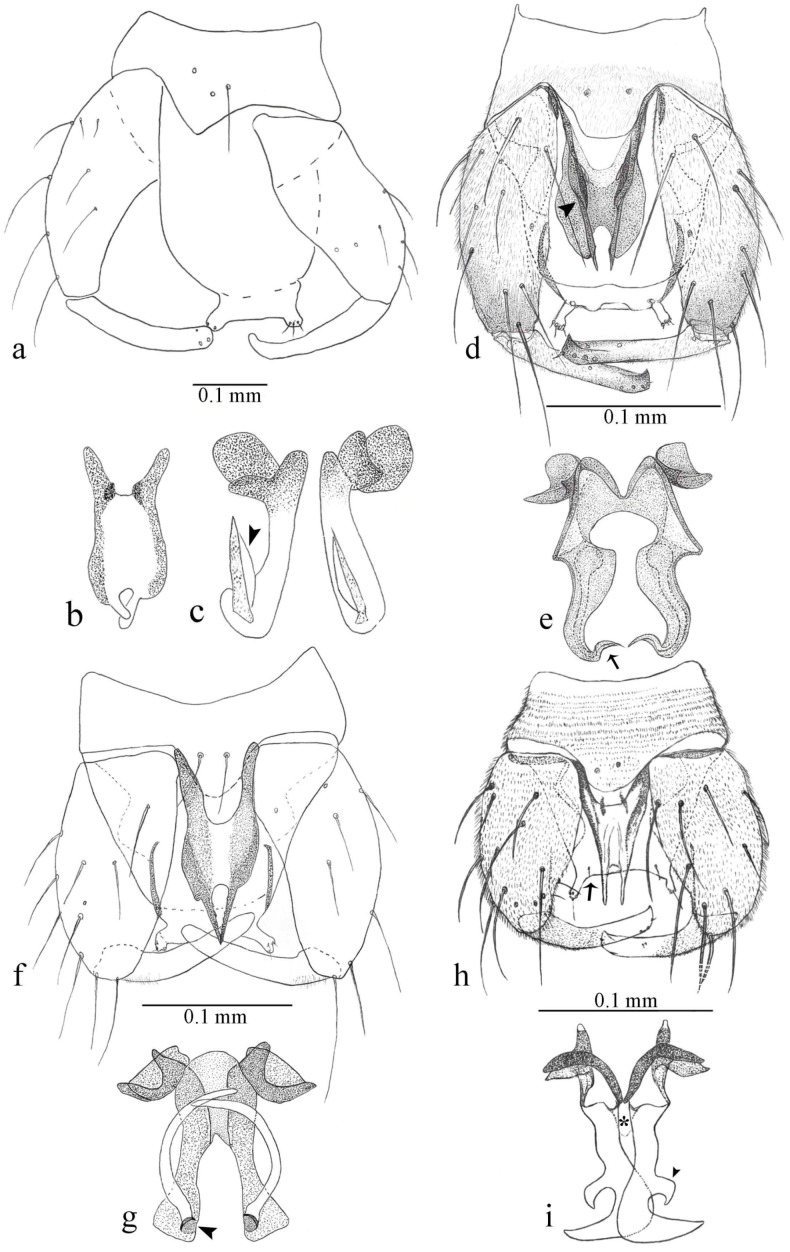
*Downeshelea chiapasi*, male: (**a**) terminalia, ventral view; (**b**) aedeagus, ventral view; (**c**) parameres, ventral view; arrowhead: membrane expansion. *Downeshelea chirusi*, male: (**d**) terminalia with aedeagus, ventral view; arrowhead: sclerotized process; (**e**) parameres, ventral view; arrow: distal portion. *Downeshelea colombiae*, male: (**f**) terminalia with aedeagus, ventral view; (**g**) parameres, ventral view; arrowhead: subapical process. *Downeshelea curta* sp. nov., male: (**h**) terminalia with aedeagus, ventral view; arrow: tergite 9; (**i**) parameres, ventral view; asterisk: anterior membrane; arrowhead: median process.

Figure 18a–g; Figure 19h,i, and Figure 21; Table 1.

**Diagnosis**. Male: only species of *Downeshelea* in the Americas with the following combination of character states: r_3_ with apical grayish spot (Figure 18a); legs brown (Figure 18c); tergite 9 short (Figure 19h); parameres fused on basal portion by pointed membrane (Figure 19i), stem with hook-like median process (Figure 19i) and apex curved externally (Figure 19i). Female: only species of *Downeshelea* in the Americas with the following combination of character states: wing (Figure 18d) and legs as described for male; hind leg claw 1.14–1.31 as long as tarsomere 5 (Figure 18g); unequal spermathecae (Figure 18f).

**Description. Male**. **Head** (Figure 18b). Eyes separated dorsomedially by 2× width of an ommatidium (Figure 18b). Antenna pale, except basal portion of flagellomere 1, distal portion of flagellomere 10, flagellomeres 11–13 brown; antennal ratio 0.93–1.02 (0.97, n = 4). Palpus with segment 3 swollen with broad, deep sensory pit; palpal ratio 2.00–2.50 (2.31, n = 4). **Thorax**. Scutum brown, without definite pattern in slide mounted specimens; pleura pale brown. Wing (Figure 18a) with five grayish areas: three in apical portion of r_3_, m_1,_ M_1,_ not reaching wing margin, spots in m_1,_ M_1_ contiguous in some specimens; one sigmoid-shaped extending from m_1_ to wing margin in m_2_; one over CuA_1,_ CuA_2_ extending into cua_1,_ anal cell, reaching wing margin; 2nd radial cell twice length of 1st; length 0.85–0.90 (0.88, n = 4) mm; width 0.30 (n = 4) mm; costal ratio 0.74–0.75 (0.74, n = 4). Halter dark brown. Legs (Figure 18c) brown, hind leg darker; hind tibial comb with six spines. Foretarsomere 1 with one basal, one apical spine; midtarsomere 1 with 1–2 ventral spines, apical spines of tarsomeres 2–4 of fore-, mid-, hind legs: 2-2-4(3), 2-2-4, 1-1-2; foretarsal ratio 2.28–2.47 (2.41, n = 4), midtarsal ratio 2.20–2.40 (2.31, n = 4), hind tarsal ratio 2.04–2.20 (2.11, n = 4); claws 0.38–0.46 (0.41, n = 4) length of their respective tarsomere 5. **Abdomen**. Brown. Terminalia (Figure 19h): tergite 9 short, not reaching the apex of gonocoxite (Figure 19h), with quadrate apex, apicolateral process long, slender; sternite 9 slightly concave anteriorly, posterior margin with moderately convex median lobe bearing two long setae. Gonocoxite brown, stout, 1.75–1.96 (1.87, n = 4) times longer than basal width; gonostylus brown, slightly curved in half length, 0.65–0.71 (0.69, n = 3) length of gonocoxite. Parameres (Figure 19i) 1.22–1.39 (1.29, n = 3) length of aedeagus, fused on basal portion by pointed membrane (Figure 19i) for 0.16 (n = 3) of total length, each with basal arm trilobed, knob slender; stem moderately sinuous, with hook-like median process (Figure 19i), broad tapering distally to pointed tip externally directed. Aedeagus (Figure 19h) triangular, sclerotized laterally, with two stripped sclerotized processes on basal midportion, basal arch U-shaped, sclerotized, extending to 0.22–0.28 (0.25, n = 3) of total length; distal portion with deep mesal notch and two sclerotized pointed processes.

**Female**. Similar to male with usual sexual differences; antenna brown; basal portion of flagellomeres slightly pale; antennal ratio 0.97–1.08 (1.01, n = 4); palpal ratio 1.83–2.20 (2.00, n = 4) (Figure 18e); mandible with 7–8 teeth. Wing as in Figure 18d; length 0.95–1.15 (1.04, n = 4) mm; width 0.40–0.47 (0.42, n = 4) mm; costal ratio 0.78–0.82 (0.80, n = 4). Foretarsomere 1 with one basal, one apical spine; midtarsomere 1 with 2–5 ventral spines; apical spines of tarsomeres 2–4 of fore-, mid legs: 2-2-4, 2-2-4; foretarsal ratio 2.37–2.87 (2.57, n = 4), midtarsal ratio 2.50–2.75 (2.60, n = 4), hind tarsal ratio 2.40–2.52 (2.46, n = 4); fore-, mid legs claws 0.56–0.75 (0.66, n = 4) length of their respective tarsomeres 5; hind leg claw 1.14–1.31 (1.23, n = 4) as long as tarsomere 5 (Figure 18g). Two unequal spermathecae (Figure 18f), measuring 45–52 (49, n = 4) by 37–45 (41, n = 4) µm and 37–40 (39, n = 4) by 40 (n = 1) µm. Third rudimentary spermatheca measuring 8 µm (not visible in Figure 18f).

**Specimens examined.** Holotype male, on microscope slide, labeled “Holotype *Downeshelea curta* Santarém, Borkent and Felippe-Bauer”, “Costa Rica, Puntarenas, Parque Nacional Corcovado, Sector La Leona, Cerro Puma, 100–300 m, 16 September–6 October 2003, malaise, K. Caballlero col”. (MNCR); allotype female, on microscope slide, labeled “Allotype *Downeshelea curta* Santarém, Borkent and Felippe-Bauer”, same data as holotype except “600 m S. de Cerro Rincón, 745 m, 23 April–24 June 2002, J. Azofeifa Zuniga col.” (MNCR). Paratypes labeled as follows: 1 female, on microscope slide, same data as allotype (CCER); 1 male, on microscope slide, same data as holotype except “19 June–8 July 2003, M. Moraga, A. Azofeifa and K. Caballero cols.” (MNCR); 1 male, on microscope slide, “Puntarenas, Soa Sierpe Centro Juvenil Tropical, Aguabuena, 5 km W. Rincón, 80 m, 10 August 2001, ABC/CO2/light trap, G. Chaverri col.” (CCER); 1 male, on microscope slide, “Puntarenas, 2 km NE Tarcoles, 26 July 1993, Borkent col.” (MNCR); 2 females on microscope slide, “Alajuela, San Carlos, Reserva Florestal Arenal, Sendero Pilón, 600 m, 1–18 May 1999, malaise, G. Carballo col.” (MNCR).

**Distribution and bionomics.** This species is known from forested areas in Costa Rica (Alajuela and Puntarenas), occurring from 80 to 745 m above the sea (Figure 21).

**Etymology.** This species name refers to its short ninth tergite. (Latin—curta = short).

**Taxonomic discussion.** This species closely resembles *D. jurgeni* in having a wing pattern with grayish spots and a dark brown body. It can be distinguished by its smaller wing length of 0.85–0.90 mm (1.10–1.22 mm in *D. jurgeni*). Female specimens can be also distinguished by the mandible with 7–8 teeth (11 teeth in *D. jurgeni*) and unequal sized spermathecae (equal-sized in *D. jurgeni*). The male genitalia of both species can be easily distinguished by the form of the median process of the paramere (long hook-like process in *D. curta*; short, pointed process in *D. jurgeni*), the broad distal portion of paramere (slender in *D. jurgeni*) and the gonostylus slightly curved at midlength (straight in *D. jurgeni*).

#### 3.4.16. *Downeshelea deanei* Felippe-Bauer and Quintelas

**Figure 20:**
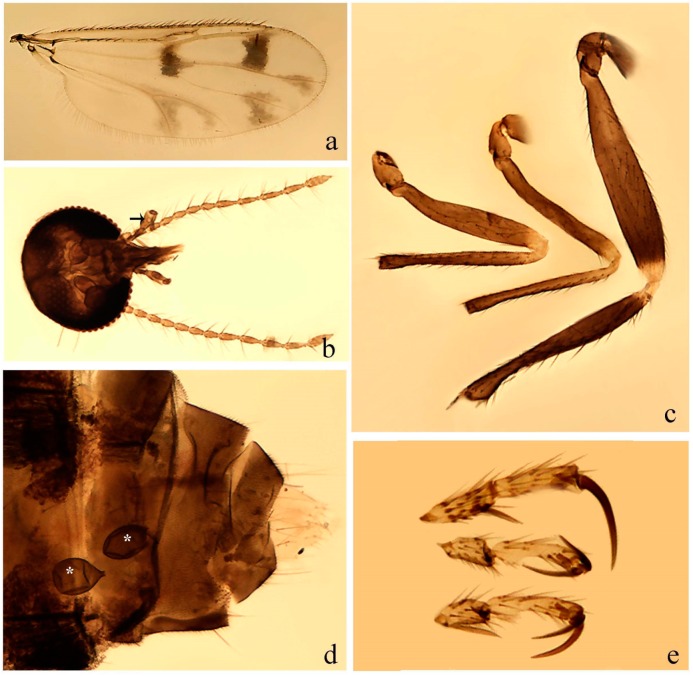
*Downeshelea deanei*, female: (**a**) wing; (**b**) head, anterior view; arrow: palpal pit; (**c**) fore-, mid, hind legs (left to right), lateral view; (**d**) apex of abdomen, ventral view; asterisks: spermathecae; (**e**) fore-, mid-, hind legs claws (bottom to top), lateral view.

**Figure 21:**
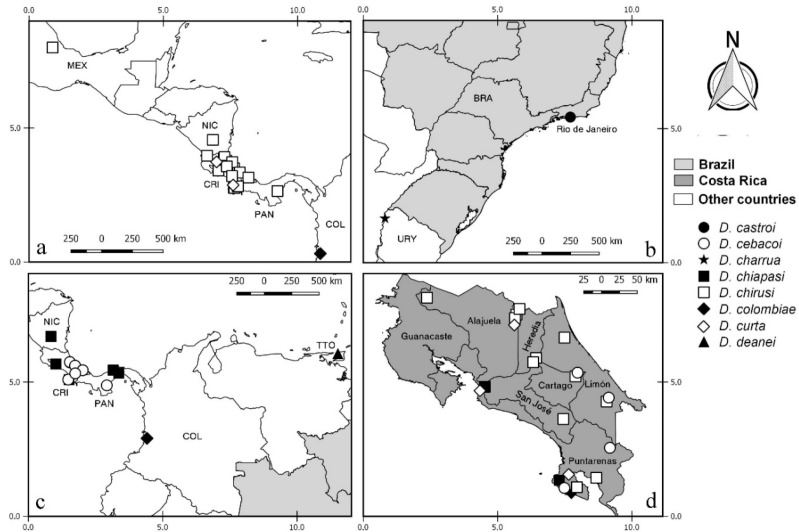
Geographic distribution of *Downeshelea castroi* (**b**), *D. cebacoi* (**c**,**d**), *D. charrua* (**b**), *D. chiapasi* (**c**,**d**), *D. chirusi* (**a**,**d**), *D. colombiae* (**a**,**c**), *D. curta* sp. nov. (**a**,**d**) and *D. deanei* (**c**).

Figure 20a–e, Figure 21, and Figure 24a–c; Table 1.

*Monohelea guianae* Lane and Wirth (not Wirth 1953, misidentification), 1964 [6]: 224 (male, female; Trinidad record).

*Downeshelea deanei:* Felippe-Bauer et al., 1995 [18]: 395 (male, Trinidad); Borkent and Wirth, 1997 [24]: 98 (in World catalog); Borkent and Spinelli, 2000 [25]: 47 (in catalog south of the USA; distribution); Borkent and Spinelli, 2007 [26]: 80 (in Neotropical catalog; distribution); Borkent, 2016 [22]: 124 (in World catalog).

**Diagnosis.** Male: only species of *Downeshelea* in the Americas with the following combination of character states: r_3_ without apical grayish spot; legs brown; paramere swollen on midportion with long horn-like process (Figure 24c), the right longer than the left one; distal portion sharp, curved, internally directed (Figure 24c); aedeagus rectangular, distal portion with two long, sharp membranous lobes (Figure 24b). Female: only species of *Downeshelea* in the Americas with the following combination of character states: wing (Figure 20a) and legs (Figure 20c) as described for male; hind leg claw 1.11–1.25 as long as tarsomere 5 (Figure 20e); equal-sized spermathecae (Figure 20d).

**Description. Female**. Similar to male described by Felippe-Bauer et al. [18]. Antenna brown; basal portion of flagellomeres 2–8 slightly pale; antennal ratio 1.00–1.06 (1.02, n = 3); palpal ratio 1.80–2.40 (2.19, n = 4) (Figure 20b); mandible with 9–10 teeth. Wing as in Figure 20a; length 1.07–1.15 (1.12, n = 3) mm; width 0.45–0.48 (0.47, n = 3) mm; costal ratio 0.78–0.79 (0.79, n = 3). Legs (Figure 20c) brown. Foretarsomere 1 with one basal, one apical spine; midtarsomere 1 with 3–7 ventral spines; apical spines of tarsomeres 2–4 of fore-, mid legs: 2-2-3, 2-2-3; foretarsal ratio 2.26–2.55 (2.37, n = 4), midtarsal ratio 2.39–2.65 (2.51, n = 4), hind tarsal ratio 2.33–2.47 (2.40, n = 4); fore-, mid- leg claw 0.65–0.72 (0.69, n = 4) length of their respective tarsomeres 5; hind leg claw 1.11–1.25 (1.15, n = 4) as long as tarsomere 5 (Figure 20e). Two equal-sized spermathecae (Figure 20d), measuring 50–57 (54, n = 3) by 37–42 (41, n = 3) µm. Third rudimentary spermatheca measuring 12.5 µm (not visible in Figure 20d).

**Specimens examined.** 1 male, Canada Balsam on four celluloid strips on pin, labeled “Holotype *Downeshelea deanei* Felippe-Bauer and Quintelas”, “*Monohelea guianae* Wirth”, TRINIDAD, Port of Spain, June 1953, U.S. Army 25 Med. Det., light trap” (FSP); 2 females labeled “*Downeshelea guianae* (Wirth)”, “BWI Tembladora USNS, 14 February 1958, light trap, T.H.G. Aitken col.” (USNM); 1 female labeled “*Downeshelea guianae* (Wirth)”, same data except “Bush Area, Nariva Swamp, 1–13 October 1959” (USNM); 1 female labeled “*Downeshelea guianae* (Wirth)”, same data except “Esperanza Estate, Vega de Oropouche, 24 March 1960” (CCER).

**Distribution.** This species is known only from Trinidad (Figure 21), occurring in coastal and humid forested areas from sea level to 60 m above sea level.

**Taxonomic discussion.** This species was described by Felippe-Bauer et al. [18] based on a previous misidentification of a male specimen of *D. guianae* (Wirth) [6]. In that paper, the authors described a single male specimen from Trinidad and did not consider the description of the female by Lane and Wirth [6], because they did not have in hand the female specimens to confirm the identification. Herein, we describe the female specimens from Trinidad (Port of Spain, Nariva Swamp and Vega de Oropouche) as *D. deanei*, previously misidentified as *D. guianae* by Lane and Wirth [6]. The female from *D. deanei* can be easily distinguished from *D. guianae* by the presence of ventral spines on midtarsomere 1 (absent in *D. guianae*).

#### 3.4.17. *Downeshelea divergentis* sp. nov.

**Figure 22:**
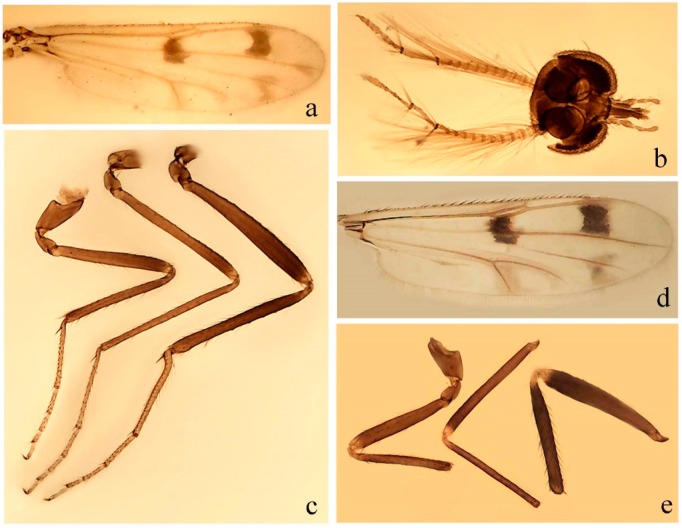
*Downeshelea divergentis* sp. nov., male: (**a**) wing; (**b**) head, anterior view; (**c**) fore-, mid-, hind legs (left to right), lateral view. *Downeshelea fluminensis*, male: (**d**) wing; (**e**) fore-, mid, hind legs (left to right), lateral view.

Figure 22a–c, Figure 24d,e, and Figure 29; Table 1.

**Diagnosis**. Male: only species of *Downeshelea* in the Americas with the following combination of character states: r_3_ without apical grayish spot (Figure 22a); legs brown, all femora slightly darker subapically (Figure 22c); paramere stem divergent in distal half (Figure 24e); distal portion bifid, inner projection slender, with tip bent to sharp point (Figure 24e), outer projection ending in membranous lobe (Figure 24e); aedeagus subtriangular (Figure 24d), basal arch covered by ventral membrane forming two rounded lobes (Figure 24d). Female unknown.

**Description. Male**. **Head** (Figure 22b). Eyes separated dorsomedially by 2.5× width of an ommatidium (not visible in Figure 22b). Antenna brown; antennal ratio 1.14. Palpus with segment 3 slightly swollen on midportion with broad, deep sensory pit; palpal ratio 2.40. **Thorax**. Scutum brown, postscutellum, pleura pale brown. Wing (Figure 22a) with three grayish area: first near apex of M_1_, not reaching wing margin; second, sigmoid-shaped, extending from m_1_ to wing margin in m_2_; third over CuA_1_, CuA_2_ extending into cua_1,_ anal cell, reaching wing margin; 2nd radial cell 2.6 length of 1st; length 1.15 mm; width 0.40 mm; costal ratio 0.76. Halter brown. Legs (Figure 22c) brown, hind leg darker, all femora slightly darker subapically; hind tibial comb with seven spines. Foretarsomere 1 with one basal, one apical spine; midtarsomere 1 without ventral spines; apical spines of tarsomeres 2–4 of fore-, mid-, hind legs: 2-2-2, 2-2-2, 1-1-1; foretarsal ratio 2.68, midtarsal ratio 2.65, hind tarsal ratio 2.23; claws 0.42 length of their respective tarsomere 5. **Abdomen**. Brown. Terminalia (Figure 24d): tergite 9 nearly triangular, abruptly tapering distally, with quadrate apex, apicolateral process long, broad; sternite 9 straight anteriorly, posterior margin with prominent median lobe bearing two long setae. Gonocoxite brown, slender, 2.74 times longer than basal width; gonostylus brown, nearly straight, 0.71 length of gonocoxite. Parameres (Figure 24e) 1.13 length of aedeagus, fused on basal portion for 0.16 of total length, each with basal arm trilobed, knob slender; stem straight in basal ½, distal ½ sinuous, divergent; distal portion bifid, outer projection ending in membranous lobe (Figure 24e); inner one slender with tip bent ventrally to sharp point (Figure 24e). Aedeagus (Figure 24d) subtriangular, basal arch U-shaped, sclerotized, covered by ventral membrane forming two rounded lobes (Figure 24d), extending to 0.35 of total length; distal portion with deep mesal notch and two strong sclerotized processes.

**Female**. Unknown.

**Specimens examined.** Holotype male, on microscope slide, labeled “Holotype *Downeshelea divergentis* Santarém, Borkent and Felippe-Bauer”, “Brazil, Amazonas, Itacoatiara, Costa do Siripá, beira do rio Amazonas, 30 November 1997, CDC 10 m, Alencar, Veras cols.” (INPA).

**Distribution and bionomics.** This species is known only from the Brazilian state of Amazonas (Figure 29). It has been found in river environments up to 20 m above sea level.

**Etymology.** This species name refers to the divergent aspect of distal half of its paramere. (Latin–divergentis = divergent).

**Taxonomic discussion.** The divergent paramere with bifid distal portion is similar to that of males of *D. bifida* and *D. quechua*. Characters for distinguishing these three species are in the discussion section of *D. bifida*.

#### 3.4.18. *Downeshelea fluminensis* Felippe-Bauer and Quintelas

Figure 22d,e, Figure 24f–h, and Figure 29; Table 1.

*Downeshelea fluminensis* Felippe-Bauer and Quintelas, 1993 [16]: 33 (male, Brazil); Borkent and Wirth, 1997 [24]: 98 (in World catalog); Borkent and Spinelli, 2000 [25]: 47 (in catalog south of the USA; distribution); Borkent and Spinelli, 2007 [26]: 80 (in Neotropical catalog; distribution); Huerta et. al., 2012 [21]: 65 (Mexico record); Borkent, 2016 [22]: 124 (in World catalog); Santarém and Felippe-Bauer, 2019 [2]: 16 (Brazilian distribution).

**Diagnosis.** Male: only species of *Downeshelea* in the Americas with the following combination of character states: r_3_ without apical grayish spot (Figure 22d); legs brown (Figure 22e); 3rd palpal segment elongate; parameres separated (Figure 24h), stem with well-developed beak-shaped process on midportion (Figure 24h); aedeagus triangular, with two basal sclerotized anteriorly-directed horn-like process (Figure 24g), distal portion with two short lobes, each with ventral sclerotized process and dorsal membrane expansion (Figure 24g). Female unknown.

**Specimens examined.** 1 male, on microscope slide, labeled “Holotype *Downeshelea fluminensis* Felippe-Bauer and Quintelas”, “Brazil, Rio de Janeiro, Arraial do Cabo, Figueira, Rua São Januário (−22.942578 S, −42.179067 W), 29 March 1989, FEEMA col.” (CCER); 1 male, on microscope slide, labeled “Paratype *Downeshelea fluminensis* Felippe-Bauer and Quintelas”, same data except ” Itaboraí, Centro, Rua Dr. Mesquita (−22.744228 S, −42.859739 W), 22 May 1989” (CCER); 1 male, same data except “Casimiro de Abreu, Centro, Rua Padre Anchieta (−22.4795 S, −42.198831), 10 April 1989” (CCER); 1 male, same data except “São Pedro da Aldeia, Estação, Rua Coronel F. Pinheiro, 43 (−22.839199 S, −42.102798 W,), 27 March 1989”(CCER); 1 male, on microscope slide, “Espírito Santo, Pancas, São Bento, Monumento Natural dos Pontões Capixabas, casa, 08.II.2011, CDC light trap, I.S. Pinto col.” (CCER) (NEW RECORD); 1 male, pinned with genitalia in drop of Canada Balsam, labeled “*Downeshelea multilineata*”, “Mato Grosso, Salobra, 18 January 1955, Travassos Junior, Barros and Albuquerque col.”, (actually Mato Grosso do Sul) (FSP) (NEW RECORD); 1 male, on microscope slide, labeled “*Downeshelea balboa* (Lane and Wirth)”, “Belize, Hattieville. 8 July 1968, light trap, W. Haase col.”(USNM) (NEW RECORD); 1 male, on microscope slide, labeled “*Downeshelea balboa* (Lane and Wirth)”, “EL SALVADOR, San Vicente, Santo Domingo, September 1966, F.S. Blanton col.” (USNM); 1 male same data except: “9 July 1966, J.F. Matto col.”(USNM) (NEW RECORD); 1 male, on microscope slide, labeled “*Downeshelea balboa* (Lane and Wirth)”, “Honduras, Santa Rosa de Copán, 26 June 1966, J.F. Matto col.” (USNM); 1 male same data except October 1966, F.S. Blanton col. “ (USNM) (NEW RECORD).

**Distribution.** This species is known from Mexico (Oaxaca, Veracruz), Belize, El Salvador, Honduras and Brazil (Mato Grosso do Sul, Espírito Santo, Rio de Janeiro) (Figure 29). It has been found in humid areas occurring from 10 m in Brazil (Rio de Janeiro) to 1100 m in Mexico (Oaxaca).

**Remarks**. Santarém et al. [10] studied material previously identified as *D. multilineata* deposited in FSP and concluded that the Brazilian specimen from Mato Grosso was misidentified and is actually a specimen of *D. fluminensis*. The specimens from Belize, El Salvador and Honduras were originally labeled by Wirth as *D. balboa*. Characters for distinguishing both species and other related species are in the discussion section of *D. balboa*.

#### 3.4.19. *Downeshelea fuscipennis* (Lane and Wirth)

**Figure 23:**
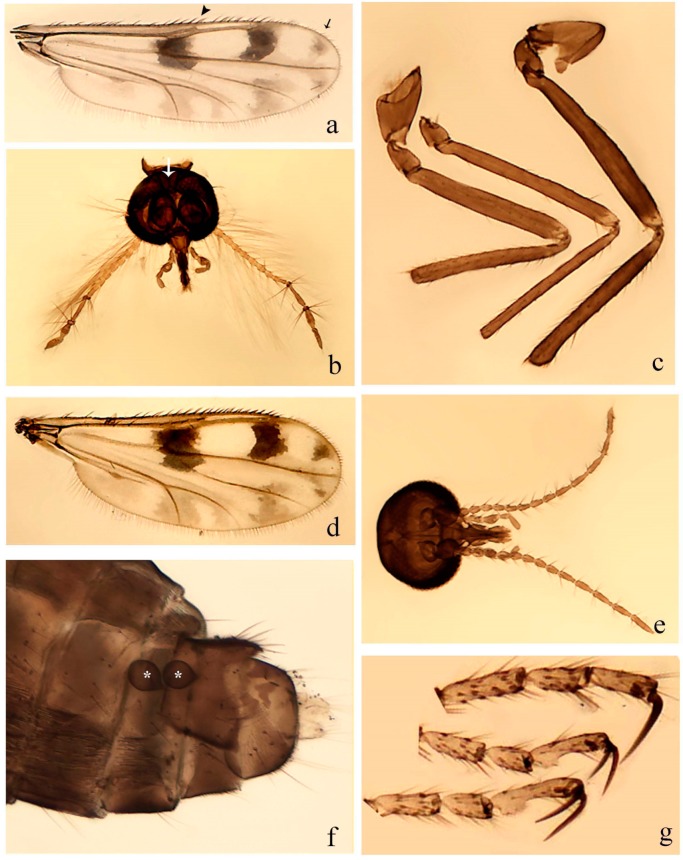
*Downeshelea fuscipennis*, male: (**a**) wing; arrow: macrotrichia; arrowhead: bristles on costa; (**b**) head, anterior view; arrow: eyes separation; (**c**) fore-, mid-, hind legs (left to right), lateral view. Female: (**d**) wing; (**e**) head, anterior view; (**f**) apex of abdomen; asterisks: spermathecae; (**g**) fore-, mid-, hind legs claws (bottom to top), lateral view.

**Figure 24:**
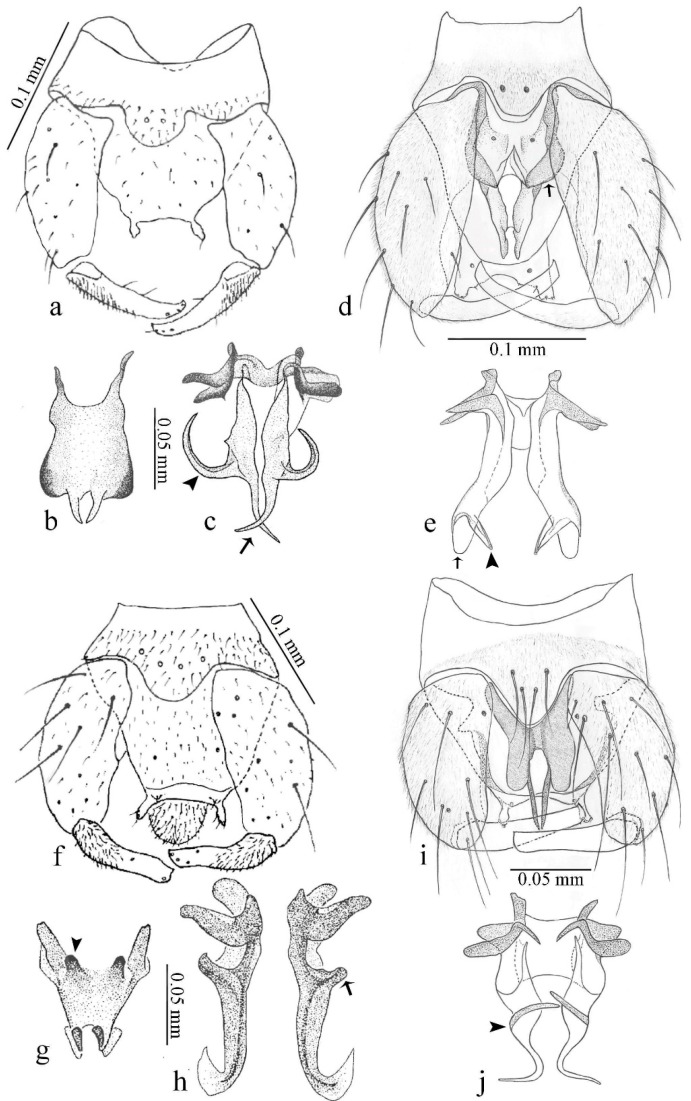
*Downeshelea deanei*, male: (**a**) terminalia, ventral view; (**b**) aedeagus, ventral view; (**c**) parameres, ventral view; arrowhead: median process; arrow: distal portion. *Downeshelea divergentis* sp. nov., male: (**d**) terminalia with aedeagus, ventral view; arrow: basal arch ventral membrane; (**e**) parameres, ventral view; arrowhead: distal portion inner projection; arrow: distal portion outer projection. *Downeshelea fluminensis*, male: (**f**) terminalia, ventral view; (**g**) aedeagus, ventral view; arrowhead: horn-like process; (**h**) parameres, ventral view; arrow: median process. *Downeshelea fuscipennis*, male: (**i**) terminalia with aedeagus, ventral view; (**j**) parameres, ventral view; arrowhead: median process.

Figure 23a–g, Figure 24i,j, and Figure 29; Table 1.

*Monohelea fuscipennis* Lane and Wirth, 1964 [6]: 221 (female; Colombia); Wirth, 1974 [3]: 41 (in catalog south of the USA; distribution).

*Downeshelea fuscipennis*: Wirth and Grogan, 1988 [7]: 51 (combination); Borkent and Wirth, 1997 [24]: 98 (in World catalog); Borkent and Spinelli, 2000 [25]: 47 (in catalog south of the USA; distribution); Borkent and Spinelli, 2007 [26]: 80 (in Neotropical catalog; distribution); Borkent, 2016 [22]: 124 (in World catalog).

**Diagnosis**. Male: only species of *Downeshelea* in the Americas with the following combination of character states: wing with extensive dark markings, r_3_ with apical grayish spot (Figure 23a); legs brown (Figure 23c); paramere with median horn-like process (Figure 24j); distal portion tapering to pointed tip, externally directed (Figure 24j); aedeagus rectangular, basal arch deep (Figure 24i). Female: only species of *Downeshelea* in the Americas with the following combination of character states: wing (Figure 23d) and legs as described for male; hind leg claw 1.00–1.27 as long as tarsomere 5 (Figure 23g); subequal spermathecae (Figure 23f).

**Description. Male**. **Head** (Figure 23b). Eyes contiguous in lower portion (Figure 23b). Antenna brown, except distal portion of flagellomere 10, flagellomeres 11–13 dark brown; antennal ratio 0.94–1.04 (0.98, n = 3). Palpus with segment 3 short, swollen with broad, deep sensory pit; palpal ratio 2.00 (n = 3). **Thorax**. Scutum dark brown, without definite pattern in slide mounted specimens; pleura dark brown. Wing (Figure 23a) with six extensive grayish areas: one over M_1_, not reaching wing margin, extending from distal dark spot to other two grayish areas in apical portion of cells r_3_, m_1_ reaching wing margin; one nearly quadrate, extending from distal ½ of m_1_ to wing margin in m_2_; one over CuA, CuA_1,_ CuA_2_ extending into cua_1,_ anal cell, broadly abutting wing margin in CuA_2_; one, faint, in middle of anal cell extending from CuA, not reaching wing margin; 2nd radial cell twice length of 1st; length 0.92–1.00 (0.97, n = 3) mm; width 0.32–0.35 (0.33, n = 3) mm; costal ratio 0.73–0.75 (0.74, n = 3). Halter dark brown. Legs (Figure 23c) brown, hind leg darker; hind tibial comb with six spines. Foretarsomere 1 with one basal, one apical spine; midtarsomere 1 with two ventral spines; apical spines of tarsomeres 2–4 of fore-, mid-, hind legs: 2-2-4, 2-2-4, 1-1-2; foretarsal ratio 2.44–2.71 (2.58, n = 2); midtarsal ratio 2.38–2.50 (2.45, n = 3), hind tarsal ratio 2.17–2.42 (2.27, n = 3); claws 0.33–0.42 (0.39, n = 3) length of their respective tarsomere 5. **Abdomen**. Dark brown. Terminalia (Figure 24i): tergite 9 with slightly concave apex, apicolateral process long, slender; sternite 9 slightly concave anteriorly, posterior margin with prominent convex median lobe bearing 4–5 long setae. Gonocoxite dark brown, stout, 1.88–2.04 (1.96, n = 3) times longer than basal width; gonostylus dark brown, short, straight, 0.72–0.77 (0.74, n = 3) length of gonocoxite. Parameres (Figure 24j) 1.04–1.26 (1.17, n = 3) length of aedeagus, fused on basal portion for 0.23–0.28 (0.25, n = 3) of total length, each with basal arm trilobed, knob bulbous; stem sinuous, expanded basally, tapering distally; midportion with curved, slender horn-like process anteromesally directed (Figure 24i); distal portion greatly curved, externally directed. Aedeagus (Figure 24i) rectangular, sclerotized; basal arch deep, U-shaped, extending to 0.44–0.52 (0.49, n = 3) of total length, basal arms heavily sclerotized; distal portion with deep mesal notch and two sclerotized pointed processes.

**Redescription. Female**. Similar to male with usual sexual differences; antenna brown; antennal ratio 0.95–1.10 (1.01, n = 10); palpal ratio 1.83–2.20 (1.98, n = 11); mandible with 9–10 teeth (Figure 23e). Wing as in Figure 23d; length 0.85–1.05 (0.94, n = 11) mm; width 0.37–0.42 (0.40, n = 11) mm; costal ratio 0.75–0.79 (0.77, n = 11). Foretarsomere 1 with one basal, two apical spines; midtarsomere 1 with 1–3 ventral spines; apical spines of tarsomeres 2–4 of fore-, mid legs: 2-2-3, 2-2-3; foretarsal ratio 2.19–2.39 (2.31, n = 11), midtarsal ratio 2.47–2.80 (2.57, n = 11), hind tarsal ratio 2.50–2.64 (2.56, n = 11); fore-, mid legs claws 0.63–0.77 (0.70, n = 11) length of their respective tarsomeres 5; hind leg claw 1.00–1.27 (1.14, n = 11) as long as tarsomere 5 (Figure 23g). Two subequal spermathecae (Figure 23f), measuring 42–57 (51, n = 11) by 37–45 (40, n = 10) µm and 37–52 (43, n = 11) by 35–40 (37, n = 10) µm. Third rudimentary spermatheca measuring 6 µm (not visible in Figure 23f).

**Specimens examined.** 2 males, 8 females, on microscope slide, labeled “Brazil, Acre, Parque Nacional da Serra do Divisor, Morro queimado, 10–11 November 1996, malaise, EF Morato col.” (1 male, 1 female CCER; 1 male, 7 females INPA) (NEW RECORD); 1 female on microscope slide labeled “Amazonas, Manaus, km50 BR174, 11.5 m, 22 July 1979, CDC light trap, J. Arion col.” (CCER) (NEW RECORD); 1 female on microscope labeled “Colombia, Rio Raposo, 3–4 June 1964, light trap, V.H. Lee col.” (USNM); 1 male same data except “28 July 1964” (USNM); 3 females on microscope slide, “Antioquia Dept. near Rio Anori Tropic rain forest, September 1970, black light trap, D.G. Young and V.H. Lee col.” (USNM).

**Distribution and bionomics.** This species is known from Colombia and Brazil (Acre and Amazonas) (Figure 29). It has been found in mangrove and humid forested areas occurring from sea level in Colombia to 300 m above sea level in Brazil.

**Taxonomic discussion.** This species was described from Colombia based on only one female specimen (holotype #67564 USNM). In addition, other male and female specimens from Colombia were labeled by Wirth as *D. fuscipennis*. Considering that these specimens match the original description, we describe herein the first male of this species and redescribe the female based on these and other specimens from Brazil. The male genitalia of *Downeshelea fuscipennis* most closely resembles that of *D. rodriguezi*, but can be distinguished by the paramere with slender median horn-like process (broad horn-like process in *D. rodriguezi*), by the distal portion greatly bent externally (bent externally to a short, sharp point in *D. rodriguezi*). These two species also have body coloration similar to that of *D. kuna* and *D. wirthiana*, but these two species do not have a paramere with a median process. Also, the distal portion of paramere in *D. kuna* is a short, slender, spiral, anteriorly directed and in *D. wirthiana* it is very long, slender with flattened point, and directed posteriorly.

#### 3.4.20. *Downeshelea gladius* sp. nov.

**Figure 25:**
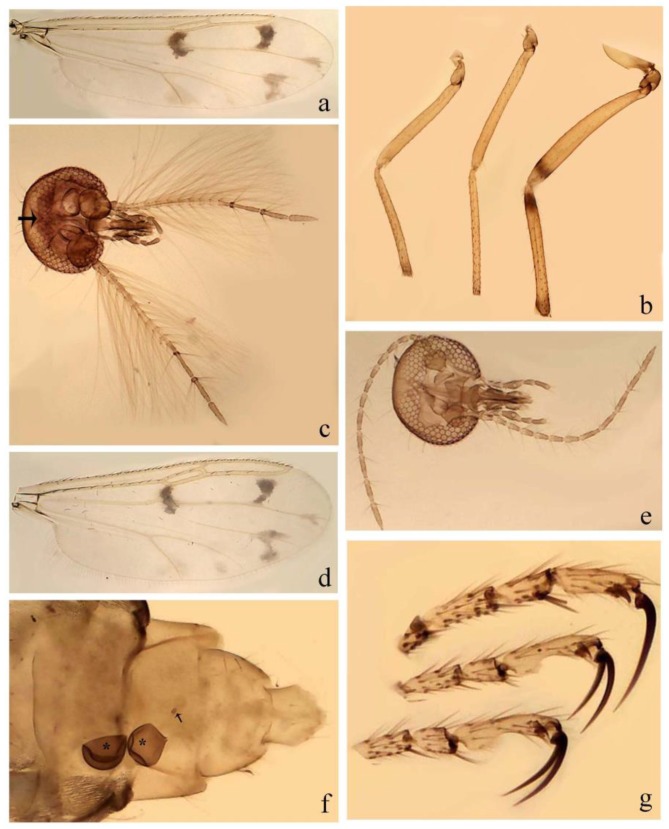
*Downeshelea gladius* sp. nov., male: (**a**) wing; (**b**) fore-, mid-, hind legs (left to right), lateral view; (**c**) head, anterior view; arrow: eyes separation. Female: (**d**) wing; (**e**) head, anterior view; (**f**) apex of abdomen; asterisks: spermathecae; arrow: 3rd rudimentary spermatheca (**g**) fore-, mid-, hind legs claws (bottom to top), lateral view.

Figure 25a–g, Figure 28a,b, and Figure 29; Table 1.

**Diagnosis**. Male: only species of *Downeshelea* in the Americas with the following combination of character states: r_3_ without apical grayish spot (Figure 25a); legs yellowish brown, narrow dark bands in hind femur subapically, hind tibia subbasally, apically (Figure 25b); gonostylus yellowish, distal ½ brown (Figure 28a); distal portion of paramere broad, sinuous, tapering to pointed tip, directed anteriorly (Figure 28b); aedeagus subrectangular (Figure 28a). Female: only species of *Downeshelea* in the Americas with the following combination of character states: wing (Figure 25d) and legs as described for male; hind leg claw 1.29–1.45 as long as tarsomere 5 (Figure 25g); unequal spermathecae (Figure 25f).

**Description. Male**. **Head** (Figure 25c). Eyes contiguous in lower portion (Figure 25c). Antenna pale, except basal portion of flagellomere 1, distal portion of flagellomere 10, flagellomeres 11–13 brown; antennal ratio 0.98–1.03 (1.01, n = 5). Palpus with segment 3 swollen on midportion with broad, deep sensory pit; palpal ratio 2.00–2.50 (2.19, n = 5). **Thorax**. Scutum yellowish brown, without definite pattern in slide mounted specimens; pleura pale brown. Wing (Figure 25a) with four grayish area: first near apex of M_1_, not reaching wing margin; second extending from m_1_ to wing margin in m_2_; third over distal ½ of CuA_2_ slightly extending into cua_1,_ anal cell, reaching wing margin; fourth, small, in cua_1_ near CuA_1_, 2nd radial cell 2.2 length of 1st; length 1.22–1.45 (1.29, n = 5) mm; width 0.35–0.45 (0.40, n = 5) mm; costal ratio 0.77–0.78 (0.78, n = 5). Halter pale, knob brown. Legs (Figure 25b) yellowish brown, narrow dark bands in hind femur subapically, hind tibia subbasally, apically (tibia subbasal dark band absent or very faint in Colombian specimens); hind tibial comb with seven spines. Foretarsomere 1 with one basal, two apical spines; midtarsomere 1 without ventral spines; apical spines of tarsomeres 2–4 of fore-, mid-, hind legs: 2-2-4, 2-2-4, 1-1-2; foretarsal ratio 2.30–2.50 (2.41, n = 5), midtarsal ratio 2.26–2.58 (2.44, n = 5), hind tarsal ratio 2.14–2.38 (2.31, n = 5); claws 0.39–0.47 (0.42, n = 5) length of their respective tarsomere 5. **Abdomen**. Yellowish brown with ventrolateral brown marks on segments 1–7. Terminalia (Figure 28a): tergite 9 with quadrate apex, apicolateral process short; sternite 9 straight anteriorly, posterior margin with prominent convex median lobe bearing 3–4 long setae. Gonocoxite yellowish, moderately stout, 2.32–2.57 (2.41, n = 4) times longer than basal width; gonostylus yellowish, distal ½ brown (Figure 28a), slightly curved on midportion, blunt tip, 0.54–0.60 (0.57, n = 5) length of gonocoxite. Parameres (Figure 28b) 1.04–1.18 (1.09, n = 5) length of aedeagus, fused on basal portion by 0.17–0.27 (0.21, n = 5), each with basal arm trilobed, knob bulbous, anteromedial fusion rounded basally; stem broad basally, tapering distally; distal portion (Figure 28b) abruptly bent, sinuous, broad, tapering to pointed tip, directed anteriorly, 0.54–0.69 (0.62, n = 4) of total length. Aedeagus (Figure 28a) subrectangular, heavily sclerotized, basal arch U-shaped, extending to 0.29–0.38 (0.32, n = 4) of total length; distal portion with deep mesal notch and two sclerotized serrate pointed processes.

**Female**. Similar to male with usual sexual differences; antenna brown, basal portion of flagellomeres paler, except flagellomere 1; antennal ratio 0.96–1.04 (0.99, n = 3); palpal ratio 2.29–2.80 (2.42, n = 4) (Figure 25e); mandible with 9 teeth. Wing as in Figure 25d; length 1.20–1.47 (1.37, n = 4) mm; width 0.47–0.50 (0.49, n = 4) mm; costal ratio 0.85–0.88 (0.87, n = 4). Foretarsomere 1 with one basal, one apical spine; midtarsomere 1 without ventral spines; apical spines of tarsomeres 2–4 of fore-, mid legs: 2-2-3(4), 2-2-3; foretarsal ratio 2.43–2.65 (2.57, n = 4), midtarsal ratio 2.63–2.88 (2.75, n = 4), hind tarsal ratio 2.48–2.64 (2.56, n = 4); fore-, mid legs claws 0.74–0.85 (0.79, n = 4) length of their respective tarsomeres 5; hind leg claw 1.29–1.45 (1.34, n = 4) as long as tarsomere 5 (Figure 25g). Two unequal spermathecae (Figure 25f), measuring 42–57 (51, n = 4) by 37–45 (40, n = 3) µm and 37–52 (43, n = 4) by 35–40 (37, n = 3) µm. Third rudimentary spermatheca measuring 8.7 µm (Figure 25f).

**Specimens examined.** Holotype male, on microscope slide, labeled “Holotype *Downeshelea gladius* Santarém, Borkent and Felippe-Bauer”, “Costa Rica, Alajuela, Upala, PN Guanacaste, Sector San Ramon, 4.75 km SW dos rios de Upala, 860 m, 17 August–17 April 1996, malaise, Quesada col.” (MNCR). Allotype female, on microscope slide, labeled “Allotype *Downeshelea gladius* Santarém, Borkent and Felippe-Bauer”, “Costa Rica, Limón, Pococi, PN Braulio Carrillo, Est. Quebrada Conzáles, 400–500 m, 15 October 2005, malaise, P Hanson, C Godoy cols.” (MNCR). Paratypes labeled as follows: 1 female, on microscope slide, same data as allotype except “24 April 2002” (MNCR); 1 male, on microscope slide, same data except “4 July 2002” (CCER); 1 male, on microscope slide, “Alajuela, San Carlos, Pital, Boca Tapada, Finca de Sergio Murillo 50–100 m, 21 July 2004, light trap, B Hernández col.” (MNCR); 1 female, on microscope slide, “Cartago, PN Barbilla, Send Principal a Rio Barbilla, 500 m, 13 May–12 June 2002, red de golpe, E Rojas col.” (CCER); 1 female, on microscope slide, “Limón, Cahuita, Reserva Chimuri, Sendero alrededor de las Cabinas, 15 m, 8–9 June 2000, malaise, Spinelli, Grogan, Borkent, Picado cols.” (MNCR); 1 male, on microscope slide, “Puntarenas, Golfito, PN Corcovado, Estación Los Patos, 160 m, 9 September–9 October 2001, K Caballero col.” (MNCR); 1 male, on microscope slide, “Colombia, Valle Rio Raposo, June 1965, light trap, VH Lee col.” (CCER); 1 male, 2 females, on microscope slide, same data except August 1965” (1 male USNM), March 18964” (1 female CCER)”, “15 April 1964” (1 female USNM).

**Distribution and bionomics.** This species is known from Costa Rica (Alajuela, Puntarenas, Cartago and Limón) and Colombia (Figure 29). It has been found in forested and coastal areas occurring at sea level in Colombia and in Costa Rica from 15 m to 860 m above sea level.

**Etymology.** This species name refers to the similarity of its distal portion of the paramere to a sword that was an ancient Roman weapon (Latin—gladius = sword).

**Taxonomic discussion.***Downeshelea gladius* most closely resembles *D. spatha* in the yellowish pattern of the legs with dark bands, the wing pattern of grayish spots, male terminalia and distal portion of paramere similar to a sword. It can be distinguished by the pale base of the hind femur (brown *in D. spatha*), the gonostylus yellowish with distal ½ brown (entirely brown in *D. spatha*), and parameres anteromedial fusion rounded basally and distal portion more sinuous and slender (anteromedial fusion and distal portion nearly straight in *D. spatha*). Characters for distinguishing both species from other species with similar wing and dark bands on the hind legs pattern are in the discussion section of *D. avizi.*

#### 3.4.21. *Downeshelea grogani* Huerta, Felippe-Bauer, and Spinelli

**Figure 26:**
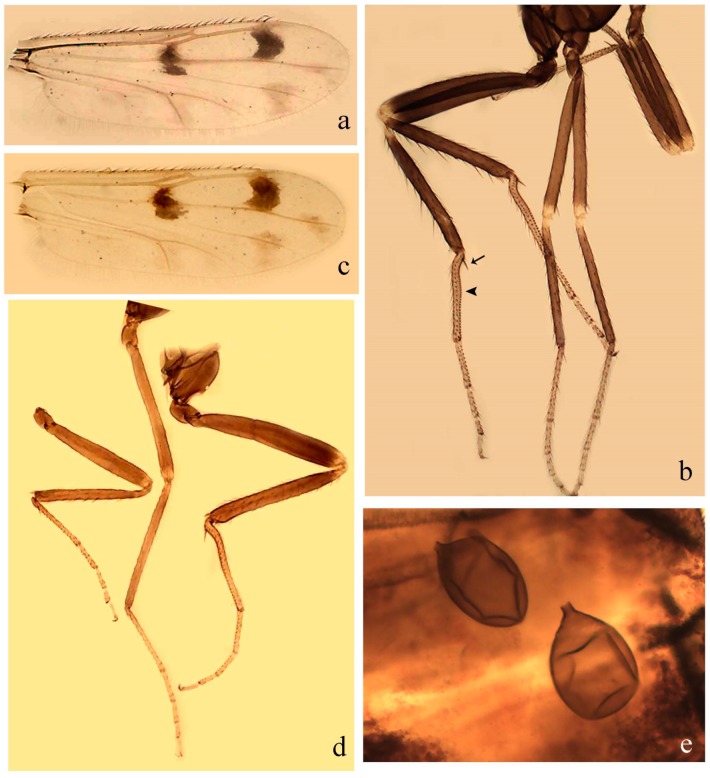
*Downeshelea grogani*, male: (**a**) wing; (**b**) fore-, mid-, hind legs (right to left), lateral view; arrow: hind tarsomere 1 basal spine; arrowhead: ventral palisade setae. *Downeshelea guianae*, male: (**c**) wing; (**d**) fore-, mid-, hind legs (left to right), lateral view. Female: (**e**) spermathecae, ventral view.

Figure 26a,b, Figure 28c–e, and Figure 29; Table 1.

*Downeshelea grogani* Huerta et al., 2012 [21]: 64 (male, Mexico); Borkent, 2016 [22]: 124 (in World catalog).

**Diagnosis.** Male: only species of *Downeshelea* in the Americas with the following combination of character states: r_3_ without apical grayish spot (Figure 26a); legs brown (Figure 26b). Male paramere stem (Figure 28e) curved laterally, gradually tapering distally to pointed, posterolaterally directed apex; aedeagus (Figure 28d) triangular, midportion with two basal ventrolateral horn-like, strongly sclerotized processes (Figure 28d); distal portion with two ventral, sclerotized process, dorsal expansion membranous (Figure 28d). Female unknown.

**Specimens examined.** 2 males, on microscope slide, labeled “Paratype *Downeshelea grogani* Huerta, Felippe-Bauer and Spinelli”, “Mexico, Veracruz, Fortin, Fortin de las Flores, June 1964, F.S. Blanton col.” (CCER; USNM); 1 male, on microscope slide, labeled “Paratype *Downeshelea grogani* Huerta, Felippe-Bauer and Spinelli”, “Quintana Roo, Puerto de Morelos, June 1961” (USNM); 4 males, on microscope slide, labeled “Paratype *Downeshelea grogani* Huerta, Felippe-Bauer and Spinelli”, “Belize, Toledo, Punta Gorda, 1.5 min W [1.5′ W], 31 July 1968, W.L. Haase, black light” (2 MLP; 1 CCER; 1 USNM); 1 male, on microscope slide, labeled “Paratype *Downeshelea grogani* Huerta, Felippe-Bauer and Spinelli”, “Colombia, Rio Raposo, 1 July 1964, light trap, V.H. Lee col.” (USNM).

**Distribution.** This species is known from Mexico (Veracruz, Quintana Roo), Belize and Colombia (Figure 29). It has been found in dry scrub forested areas and in coastal rainforest, occurring at sea level in Colombia and Belize and from 5 to 1010 m above sea level in Mexico.

#### 3.4.22. *Downeshelea guianae* (Wirth)

Figure 26c–e, Figure 28f–h, and Figure 29; Table 1.

*Monohelea multilineata*: Macfie, 1940 [29]: 187, not Lutz (misidentification; male, British Guiana).

*Monohelea guianae* Wirth, 1953 [4]: 150 (male, British Guiana); Lane and Wirth, 1964 [6]: 224 (misidentification; female, Trinidad); Wirth, 1974 [3]: 41 (in catalog south of the USA; distribution).

*Downeshelea guianae*: Wirth and Grogan, 1988 [7]: 51 (combination); Felippe-Bauer et al., 1995 [18]: 397 (redescription; male, female, Guiana and Brazil—Pará); Borkent and Wirth, 1997 [24]: 98 (in World catalog); Borkent and Spinelli, 2000 [25]: 47 (in catalog south of the USA; distribution); Borkent and Spinelli, 2007 [26]: 80 (in Neotropical catalog; distribution); Borkent, 2016 [22]: 124 (in World catalog); Santarém and Felippe Bauer, 2019 [2]: 16 (Brazilian distribution).

**Diagnosis.** Male: only species of *Downeshelea* in the Americas with the following combination of character states: r_3_ without apical grayish spot (Figure 26c); legs brown (Figure 26d); distal portion of paramere (Figure 28h) foot-shaped with externally directed lobe; aedeagus (Figure 28g) subrectangular, distal portion with two sclerotized pointed process, bearing two ventral membranous expansion (Figure 28g). Female: only species of *Downeshelea* in the Americas with the following combination of character states: wing and legs as described for male; midtarsomere 1 without ventral spines; hind leg claw 1.13–1.33 as long as tarsomere 5; equal-sized spermathecae (Figure 26e).

**Specimens examined.** 1 female, on microscope slide, labeled “TRINIDAD, BWI Tembladora USNS, 31 January 1958, light trap, T.H.G. Aitken col.” (USNM); 1 male, on microscope slide, same data except “grassy stream margin, 24 April 1963, R.N. Williams col.” (USNM) (NEW RECORD); 1 male, on microscope slide, labeled “Brazil, Pará, Belém, APEG forest, June 1970, light trap, T.H.G. Aitken col.” (USNM); 1 male, 1 female, on microscope slide, labeled “Pará, São Félix do Xingu, Rio Fresco, J. Grazia col.” (CCER); 2 males, on microscope slide, labeled “Pará, Tracuateua, Vila de Santa Maria, 27–28 February 2007, CDC light trap, Gorayeb and Guimarães col.” (MPEG); 1 male, on microscope slide, labeled “Pará, Viseu, Vila Curupaiti, 20–21 June 2007, CDC light trap, Trindade and Guimarães col.” (MPEG); 1 male, 1 female, on microscope slide, labeled “Pará, Sta. Barbara do Pará, Fazenda Morelândia, Rio Baiacú, 21–22 April 2008, CDC light trap, Trindade and Guimarães col.” (MPEG).

**Distribution.** This species is known from Trinidad, Guyana and Brazil (Pará) (Figure 29) in coastal and humid forested areas. It has been found at sea level in Trinidad, at 120 m in Guyana and from 5 to 300 m above sea level in Brazil.

**Remarks.** The Trinidad specimens previously identified as *D. guianae* by Lane and Wirth [6] were described by Felippe-Bauer et al. [18] as *D. deanei*. Herein, we examined other specimens from this country and once again record *D. guianae* as also present in Trinidad.

#### 3.4.23. *Downeshelea jurgeni* sp. nov.

**Figure 27:**
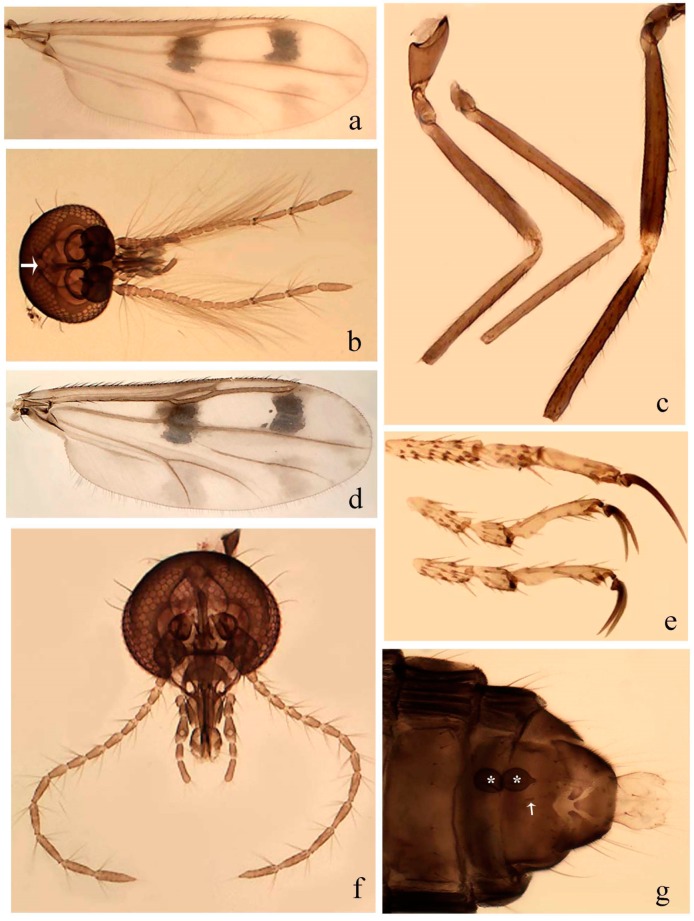
*Downeshelea jurgeni* sp. nov., male: (**a**) wing; (**b**) head, anterior view; arrow: eyes separation; (**c**) fore-, mid-, hind legs (left to right), lateral view. Female: (**d**) wing; (**e**) fore-, mid-, hind legs claws (bottom to top), lateral view; (**f**) head, anterior view; (**g**) apex of abdomen, ventral view; asterisks: spermathecae; arrow: 3rd rudimentary spermatheca.

**Figure 28:**
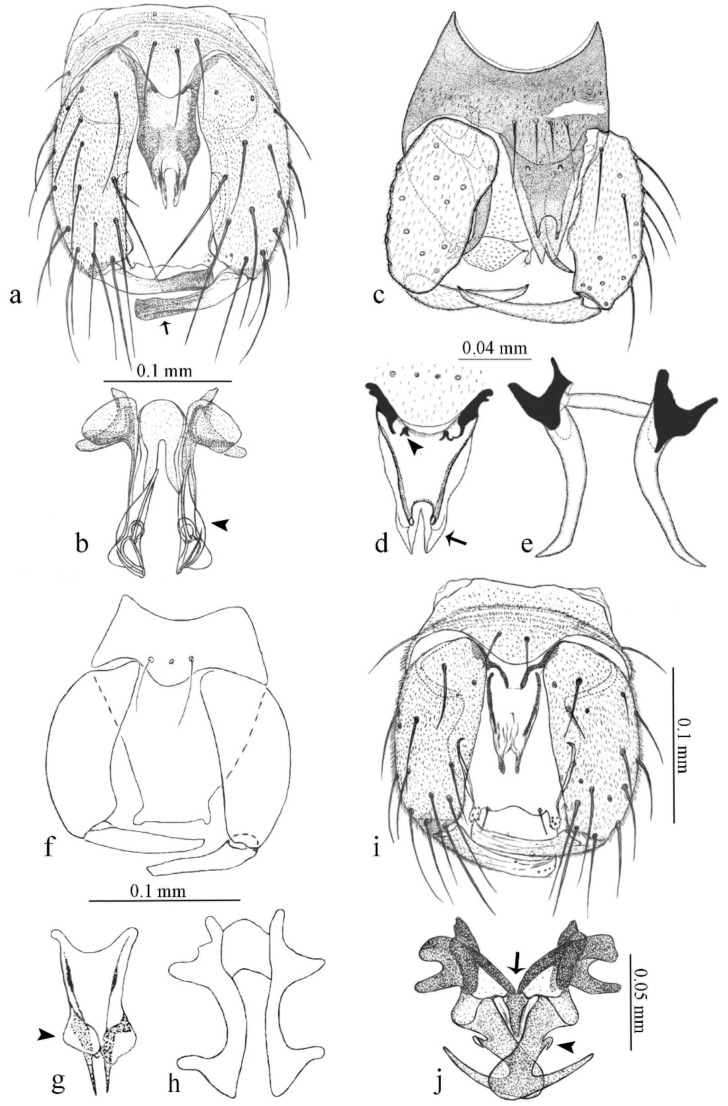
*Downeshelea gladius* sp. nov., male: (**a**) terminalia with aedeagus, ventral view; arrow: gonostylus; (**b**) parameres, ventral view; arrowhead: distal portion. *Downeshelea grogani*, male: (**c**) terminalia, ventral view; (**d**) aedeagus, ventral view; arrowhead: ventrolateral process; arrow: dorsal expansion; (**e**) parameres, ventral view. *Downeshelea guianae*, male: (**f**) terminalia, ventral view; (**g**) aedeagus, ventral view; arrowhead: ventral expansion; (**h**) parameres, ventral view. *Downeshelea jurgeni* sp. nov., male: (**i**) terminalia with aedeagus, ventral view; (**j**) parameres, ventral view; arrow: basal fused portion; arrowhead: median process.

**Figure 29:**
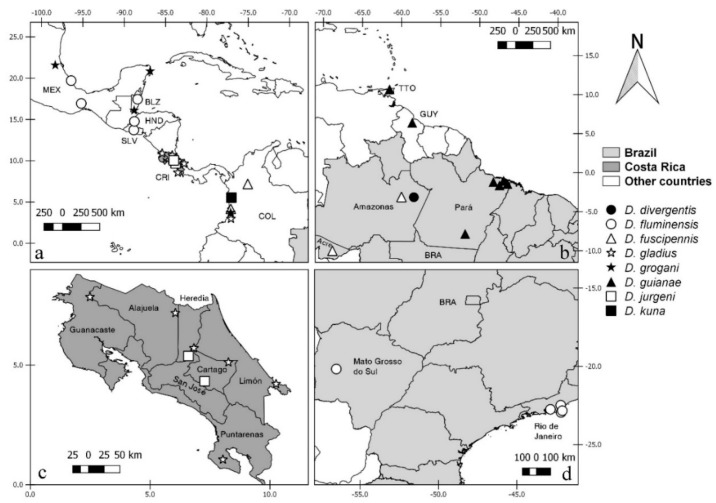
Geographic distribution of *Downeshelea divergentis* sp. nov. (**b**), *D. fluminensis* (**a**,**d**), *D. fuscipennis* (**a**,**b**), *D. gladius* sp. nov. (**a**,**c**), *D. grogani* (**a**), *D. guianae* (**b**), *D. jurgeni* sp. nov. (**a**,**c**) and *D. kuna* sp. nov. (**a**).

Figure 27a–g, Figure 28i,j, and Figure 29; Table 1.

**Diagnosis.** Male: only species of *Downeshelea* in the Americas with the following combination of character states: r_3_ with apical grayish spot (Figure 27a); legs brown (Figure 27c); parameres fused on basal portion by pointed membrane forming V-shaped arch (Figure 28j), stem broad basally with short, pointed median process direct posteriorly (Figure 28j), distal portion slender, abruptly bent laterally (Figure 28j). Female: only species of *Downeshelea* in the Americas with the following combination of character states: wing (Figure 27d) and legs as described for male; hind leg claw 1.07–1.29 as long as tarsomere 5 (Figure 27e); equal-sized spermathecae (Figure 27g).

**Description. Male**. **Head** (Figure 27b). Eyes separate dorsomedially by width of one to two ommatidia (Figure 27b). Antenna brown; antennal ratio 0.93–1.00 (0.97, n = 8). Palpus with segment 3 slightly swollen on midportion with broad, deep sensory pit; palpal ratio 2.00–2.40 (2.11, n = 8). **Thorax**. Scutum dark brown, without definite pattern in slide mounted specimens; pleura dark brown. Wing (Figure 27a) with five grayish areas: three in apical portion of r_3_, m_1,_ M_1_ reaching wing margin, spots in m_1,_ M_1_ contiguous in some specimens; one extending from m_1_ to wing margin in m_2_; one over CuA_1,_ CuA_2_ extending from mediocubital fork into cua_1,_ anal cell, reaching wing margin; 2nd radial cell 2.4 length of 1st; length 1.10–1.22 (1.16, n = 9) mm; width 0.40–0.45 (0.42, n = 9) mm; costal ratio 0.73–0.76 (0.74, n = 9). Halter dark brown. Legs (Figure 27c) brown, hind leg darker, hind femur slightly darker subapically in some specimens; hind tibial comb with six spines. Foretarsomere 1 with one basal, one apical spine; midtarsomere 1 with 1–2 ventral spines; apical spines of tarsomeres 2–4 of fore-, mid-, hind legs: 3-3-4, 2-2-3(4), 1-1-3; foretarsal ratio 2.35–2.43 (2.40, n = 9), midtarsal ratio 2.29–2.50 (2.37, n = 9), hind tarsal ratio 2.23–2.41 (2.29, n = 9); claws 0.36–0.46 (0.42, n = 9) length of their respective tarsomere 5. **Abdomen**. Dark brown. Terminalia (Figure 28i): tergite 9 with somewhat quadrate apex, apicolateral process long, slender; sternite 9 slightly concave anteriorly, posterior margin with short convex median lobe bearing 2–4 long setae. Gonocoxite brown, moderately stout, 1.92–2.17 (2.07, n = 9) times longer than basal width; gonostylus brown, nearly straight, 0.69–0.78 (0.72, n = 9) length of gonocoxite. Parameres (Figure 28j) 1.05–1.35 (1.14, n = 9) length of aedeagus, each with basal arm trilobed, knob slender, fused on basal portion by pointed membrane for 0.14–0.17 (0.16, n = 3) of total length, forming a V-shaped arch (Figure 28j); stem broad basally, moderately sinuous, with short pointed median process posteriorly directed (Figure 28j); distal portion slender, broad, abruptly bent laterally, tapering to pointed tip. Aedeagus (Figure 28i) subtriangular, with sclerotized stripes laterally, basal arch somewhat U-shaped, sclerotized, extending to 0.28–0.33 (0.30, n = 9); distal portion with moderately deep mesal notch and two sclerotized, slightly serrate, pointed processes.

**Female**. Similar to male with usual sexual differences; antenna brown; basal portion of flagellomeres 2–8 slightly pale; antennal ratio 1.01–1.04 (1.03, n = 2); palpal ratio 2.00–2.40 (2.20, n = 3) (Figure 27f); mandible with 11 teeth. Wing as in Figure 27d; length 1.32–1.43 (1.37, n = 3) mm; width 0.52–0.58 (0.56, n = 3) mm; costal ratio 0.76–0.79 (0.77, n = 3). Foretarsomere 1 with one basal, one apical spine; midtarsomere 1 with three ventral spines; apical spines of tarsomeres 2–4 of fore-, mid legs: 2-3-4, 2-2-4(3); foretarsal ratio 2.29–2.45 (2.39, n = 3), midtarsal ratio 2.33–2.70 (2.51, n = 3); hind tarsal ratio 2.43–2.50 (2.48, n = 3); fore-, mid legs claws 0.59–0.70 (0.65, n = 3) length of their respective tarsomeres 5; hind leg claw 1.07–1.29 (1.20, n = 3) as long as tarsomere 5 (Figure 27e). Two equal-sized spermathecae (Figure 27g) measuring 40–53 (45, n = 3) by 35–38 (37, n = 2) µm. Third rudimentary spermatheca measuring 9.2 µm (Figure 27g).

**Specimens examined.** Holotype male, on microscope slide, labeled “Holotype *Downeshelea jurgeni* Santarém, Borkent and Felippe-Bauer”, “Costa Rica, Cartago, Paraíso, P.N. Tapantí, 1600 m (83°46′ W;09°43′21″ N), 14–21 April 2013, malaise trap, Proyecto ZADBI col.” (MNCR). Allotype female, on microscope slide, labeled “Allotype *Downeshelea jurgeni* Santarém, Borkent and Felippe-Bauer”, “Costa Rica, Prov. San José, Moravia, Zurquí de Moravia, Tower path, 1600 m (84°0′57″ W;10°02′58″ N), 26 July–2 August 2013, emergence trap, over leaf litter, 15 m, Proyecto ZADBI col.” (MNCR). Paratypes labeled as follows: 2 males, 1 female, “Cartago, La Represa, Torre del I.C.E. entre Porras y Villegas, 1800 m, April 1997, malaise, R. Delgado col.” (1 male CCER; 1 male, 1 female LACM); 6 males same data as allotype except “10–17 May 2013, flight intercept trap 0 m” (3 MNCR); “3–10 May 2013, malaise” (CNCI); “13–20 April 2013, malaise” (MNCR); “4–11 October 2013, flight intercept trap 0 m” (CCER); 1 female same data as allotype except “1–7 June 2013, malaise” (CCER).

**Distribution and bionomics.** This species is known only from Costa Rica (San José and Cartago) (Figure 29). It has been found in forested areas from 1600 m to 1850 m above sea level.

**Etymology.** This species name refers to the grandchild of Jorge Arturo Lizano, who so generously allowed this species to be collected as part of a detailed inventory of the Diptera on his property in Costa Rica [36,37].

**Taxonomic discussion.***Downeshelea jurgeni* most closely resembles *D. curta* sp. nov. Characters for distinguishing both species are in the discussion section of that species.

#### 3.4.24. *Downeshelea kuna* sp. nov.

**Figure 30:**
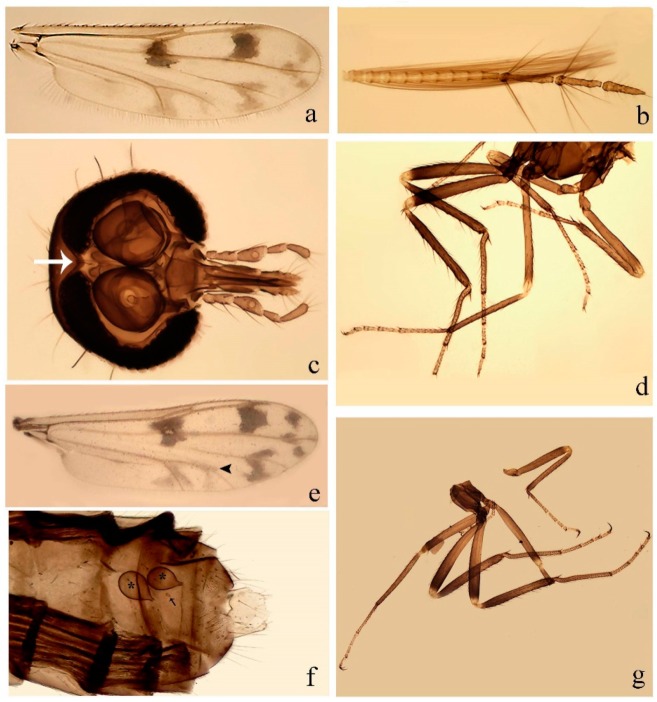
*Downeshelea kuna* sp. nov., male: (**a**) wing; (**b**) antenna; (**c**) head, anterior view; arrow: eyes separation; (**d**) fore-, mid-, hind legs (right to left), lateral view. *Downeshelea lanei*, male: (**e**) wing; arrowhead: CuA_1_. Female: (**f**) apex of abdomen, ventral view; asterisks: spermathecae; arrow: 3rd rudimentary spermatheca; (**g**) fore-, mid-, hind legs (top to bottom), lateral view.

Figure 29; Figure 30a–d, Figure 33a,b; Table 1.

**Diagnosis**. Male: only species of *Downeshelea* in the Americas with the following combination of character states: r_3_ with apical grayish spot (Figure 30a); legs brown (Figure 30d); paramere stem slender, expanded distally (Figure 33b); distal portion curling up mesally, forming a spiral directed anteriorly (Figure 33b); aedeagus triangular (Figure 33a), with sclerotized elliptical anteromesal areas (Figure 33a). Female unknown.

**Description. Male**. **Head** (Figure 30c). Eyes separated dorsomedially by 2× width of an ommatidium (Figure 30c). Antenna (Figure 30b) pale, except basal portion of flagellomere 1, distal portion of flagellomere 10, flagellomeres 11–13 brown; antennal ratio 1.10. Palpus with segment 3 slightly swollen on midportion with broad, deep sensory pit; palpal ratio 2.00. **Thorax**. Scutum brown, without definite pattern in slide mounted specimens; pleura brown. Wing (Figure 30a) with five grayish areas: three in apical portion of r_3_, m_1,_ M_1,_ reaching wing margin in r_3_, m_1_; one sigmoid-shaped extending from m_1_ to wing margin in m_2_; one over CuA_1,_ CuA_2_ extending from mediocubital fork into cua_1,_ anal cell, reaching wing margin in CuA_2_; 2nd radial cell 2.5 length of 1st; length 0.97 mm; width 0.32 mm; costal ratio 0.74. Halter dark brown. Legs (Figure 30d) brown, hind leg darker; hind tibial comb with six spines. Foretarsomere 1 with one basal, one apical spine; midtarsomere 1 without ventral spines; apical spines of tarsomeres 2–4 of fore-, mid-, hind legs: 2-2-2, 2-2-3, 1-1-3; foretarsal ratio 2.41, midtarsal ratio 2.32, hind tarsal ratio 2.04, claws 0.41 length of their respective tarsomere 5. **Abdomen**. Brown. Terminalia (Figure 33a): tergite 9 with quadrate apex, apicolateral process short (Figure 33a); sternite 9 concave anteriorly, posterior margin with large convex median lobe bearing four long setae. Gonocoxite brown, moderately stout, 2.13 times longer than basal width; gonostylus brown, curved, broad basally, 0.63 length of gonocoxite. Parameres (Figure 33b) 1.41 length of aedeagus, fused on basal portion by 0.19 of total length, each with basal arm trilobed, knob flattened; stem nearly straight, slender, expanded distally; distal portion bent, very slender, curling up mesally, forming a spiral, directed anteriorly (Figure 33b), 0.25 of total length. Aedeagus (Figure 33a) triangular, heavily sclerotized, basal arch nearly U-shaped, extending to 0.29 of total length with sclerotized elliptical anteromesal areas (Figure 33a); distal portion with deep mesal notch and two prominent sclerotized serrate processes.

**Female**. Unknown.

**Specimens examined.** Holotype male, on microscope slide, labeled “Holotype *Downeshelea kuna* Santarém, Borkent and Felippe-Bauer”, “Colombia, Dept Choco, RT.25, 12 November 1967” (USNM).

**Distribution and bionomics.** This species is known only from Colombia (Figure 29) from a coastal forest at 50 m above sea level.

**Etymology.** This species name refers to the Kuna, Amerindian people from the Choco Department, Colombia, where it was collected.

**Taxonomic discussion.** The pattern of wing and leg coloration in *D. kuna* is similar to that of *D. fuscipennis*, *D. rodriguezi* sp. nov. and *D. wirthiana* sp. nov. Characters for distinguishing these species are in the discussion section of *D. fuscipennis.*

#### 3.4.25. *Downeshelea lanei* Felippe-Bauer and Borkent

Figure 30e–g and Figure 33c,d; Figure 39; Table 1.

*Monohelea cebacoi* Lane and Wirth, 1964 [6]: 218 (male, Brazil specimens).

*Downeshelea cebacoi*: Wirth and Grogan, 1988 [7]: 51 (combination); Borkent and Wirth, 1997 [24]: 97 (in World catalog; Brazil specimens); Borkent and Spinelli, 2000 [25]: 47 (in catalog south of the USA; distribution, Brazil specimens); Borkent and Spinelli, 2007 [26]: 80 (in Neotropical catalog; distribution, Brazil specimens).

*Downeshelea lanei*: Felippe-Bauer and Borkent, 2011 [20]: 25 (male, female; Brazil—Pará); Borkent, 2016 [22]: 124 (in World catalog); Santarém and Felippe-Bauer, 2019 [2]: 16 (Brazilian distribution).

**Diagnosis.** Male: only species of *Downeshelea* in the Americas with the following combination of character states: r_3_ with apical grayish spot (Figure 30e); legs pale brown, fore-, midfemur slightly darker subapically, hind femur darker on subapical third, hind tibia darker on basal half and apical portion; distal portion of paramere long, sharply directed medially (Figure 33d); aedeagus rectangular (Figure 33c). Female: only species of *Downeshelea* in the Americas with the following combination of character states: wing and legs (Figure 30g) as described for male; hind leg claw 1.10 as long as tarsomere 5; unequal spermathecae (Figure 30f).

**Specimens examined.** 1 male, in drop of Canada Balsam on five celluloid strips on pin, labeled “Holotype *Downeshelea lanei* Felippe-Bauer and Borkent”, “Paratype, *Monohelea cebacoi*, drawn”, “Brazil, Pará, Cachimbo, I. 1956, S.J. Oliveira col.” (FSP); 1 female, on microscope slide, labeled “Allotype *Downeshelea lanei* Felippe-Bauer and Borkent”, “*Downeshelea cebacoi*”, “Brazil, Amazon River, Floresta, Prainha, 16 April 1969, H.A Wright col., light trap” (CCER); 1 male, on microscope slide, labeled “Paratype *Downeshelea lanei* Felippe-Bauer and Borkent”, “Brazil, Pará, Belém, Floresta da Área de Pesquisas Ecológicas do Guamá (APEG Forest), April 1970, THG Aitken col., light trap” (CCER); 1 female, on microscope slide, same data, except “July 1970, sticky trap” (CCER).

**Distribution.** This species is known only from Brazil (Pará) in riparian habitat and humid forested areas from 15 to 500 m above sea level (Figure 39).

#### 3.4.26. *Downeshelea magna* sp. nov.

**Figure 31:**
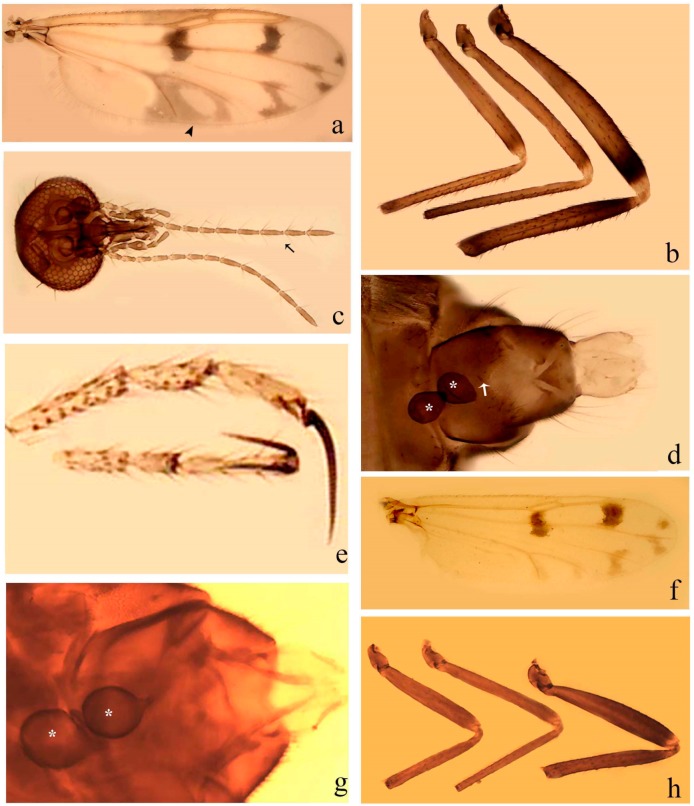
*Downeshelea magna* sp. nov., female: (**a**) wing, arrowhead: cua_1_ margin; (**b**) fore-, mid-, hind legs (left to right), lateral view; (**c**) head, anterior view; arrow: antennal distal segments; (**d**) apex of abdomen, ventral view; asterisks: spermathecae; arrow: 3rd rudimentary spermatheca; (**e**) fore-, hind legs claws (bottom to top), lateral view. *Downeshelea oliveirai*, male: (**f**) wing; (**h**) fore-, mid-, hind legs (left to right), lateral view. Female: (**g**) apex of abdomen, ventral view; asterisks: spermathecae.

Figure 31a–e, Figure 39; Table 1.

**Diagnosis**. Female: only species of *Downeshelea* in the Americas with the following combination of character states: wing length 1.63–2.00 mm; r_3_ with apical grayish spot (Figure 31a), one spot over CuA_1,_ CuA_2_ broadly extending from mediocubital fork into cua_1,_ anal cell (Figure 31a); legs brown, fore-, mid-, hind femur darker subapically; fore-, midtibia slightly darker apically; hind tibia darker subbasally, apically (Figure 31b); apical spines on tarsomeres 2–4 of hind leg: 2-2-3; hind leg claw 1.19–1.43 as long as tarsomere 5 (Figure 31e); subequal spermathecae (Figure 31d). Male unknown.

**Description. Female. Head** (Figure 31c). Antenna brown, basal portion of flagellomeres pale; antennal ratio 0.80–0.96 (0.90, n = 10). Palpus with segment 3 slightly swollen on midportion with broad, deep sensory pit; palpal ratio 1.86–2.33 (2.10, n = 11); mandible with 11–12 teeth. **Thorax**. Scutum brown, pleura pale brown. Wing (Figure 31a) with five grayish areas: three in apical portion of r_3_, m_1,_ reaching wing margin, M_1_ not reaching wing margin; one I-shaped extending from m_1_ to wing margin in m_2_; one over CuA_1,_ CuA_2_ broadly extending from mediocubital fork into cua_1,_ white spot into cua_1_ not extending to wing margin (Figure 31a); 2nd radial cell twice length of 1st; length 1.63–2.00 (1.80, n = 11) mm; width 0.63–0.80 (0.70, n = 11) mm; costal ratio 0.79–0.82 (0.80, n = 11). Halter brown. Legs (Figure 31b) brown, hind leg darker; fore-, mid-, hind femur darker subapically; fore-, midtibia slightly darker apically; hind tibia darker subbasally, apically; hind tibial comb with 7–8 spines. Foretarsomere 1 with one basal, two apical spines; midtarsomere 1 with 5–7 ventral spines; apical spines of tarsomeres 2–4 of fore-, mid-, hind legs: 3-3-4, 3-3-4, 2-2-3; foretarsal ratio 2.27–2.54 (2.36, n = 11); midtarsal ratio 2.38–2.65 (2.50, n = 11); hind tarsal ratio 2.53–2.80 (2.69); fore-, mid legs claws 0.65–0.77 (0.70, n = 11) length of their respective tarsomeres 5; hind leg claw 1.19–1.43 (1.31, n = 11) as long as tarsomere 5 (Figure 31e). **Abdomen**. Two subequal spermathecae (Figure 31d) measuring 55–73 (61, n = 11) by 45–58 (51, n = 7) µm and 53–73 (59, n = 10) by 45–53 (49, n = 7) µm. Third rudimentary spermatheca measuring 9.3 µm (Figure 31d).

**Male**. Unknown.

**Specimens examined**. Holotype female on microscope slide labeled “Holotype *Downeshelea magna* Santarém, Borkent and Felippe-Bauer”, “Costa Rica, Cartago, PN Tapantí, 1600 m, (83°46′ W;09°43′21″ N), “15–21 April 2013”, malaise, Proyecto ZABDI col.” (MNCR). Paratypes as follows: 1 females same data as holotype (MNCR); 9 females same data except: “19–26 May 2013” (1 CCER; 1 MNCR); “26 May–2 June 2013”(MNCR); “30 February–7 April 2013” (MNCR); “30 June–6 July 2013” (MNCR); “11–18 August 2013” (MNCR); “25 August–1 April 2013” (MNCR); “21–29 April 2013 (LACM); 6–13 October 2013” (LACM).

Distribution and bionomics. This species is known from Costa Rica (Cartago) in forested areas, occurring at 1600 m above sea level (Figure 39).

**Etymology**. This species name refers to its large size, a feature that is unique in *Downeshelea.* (Latin—magna = great).

**Taxonomic discussion.***Downeshelea magna* is a large-sized species (wing length 1.63–2.00 mm) compared to all other Neotropical species which the maximum female wing length is 1.57 mm (*D. jurgeni*). Besides that, the distribution of apical spines on tarsomeres 2–4 of the hind leg is 2-2-3, differing from all other known *Downeshelea* female specimens where the hind leg tarsomeres 2–4 have 1-1-2 apical spines. In addition, the white spot in cua_1_ does not extend to the wing margin and the grayish mark extending from m_1_ to m_2_ is very slender. These features are unique within the genus so that we can identify this species based only on the female.

#### 3.4.27. *Downeshelea oliveirai* Felippe-Bauer

Figure 31f–h, Figure 33e,f, and Figure 39; Table 1.

*Downeshelea oliveirai* Felippe-Bauer and Silva, 2008 [19]: 400 (male, female; Brazil – Rondônia and Pará); Borkent, 2016 [22]: 124 (in World catalog); Santarém and Felippe-Bauer, 2019 [2]: 17 (Brazilian distribution).

**Diagnosis.** Male: only species of *Downeshelea* in the Americas with the following combination of character states: r_3_ with apical grayish spot (Figure 31f); legs brown (Figure 31h); paramere stem with clear, bulbous ventral lobe (Figure 33f), with median pointed process (Figure 33f), externally directed; distal portion tapered to simple point (Figure 33f); aedeagus Y-shaped (Figure 33e). Female: only species of *Downeshelea* in the Americas with the following combination of character states: wing and legs as described for male; hind leg claw 1.06–1.18 as long as tarsomere 5; unequal spermathecae (Figure 31g).

**Specimens examined.** 1 male, on microscope slide, labeled “Holotype *Downeshelea oliveirai* Felippe-Bauer”, “Brazil, Rondônia, Rio Pacaás Novos, 08 April 1999, light trap, N. Hamada and U. Barbosa col.” (CCER); 1 female, on microscope slide, labeled “Allotype *Downeshelea oliveirai* Felippe-Bauer”, “Brazil, Pará, Belém, Floresta da Área de Pesquisas Ecológicas do Guamá (APEG Forest), February 1970, light trap, THG Aitken col.” (CCER); 4 males, 1 female, on microscope slide, labeled “Paratype *Downeshelea oliveirai* Felippe-Bauer”, same data as holotype (CCER); 2 females, on microscope slide, labeled “Paratype *Downeshelea oliveirai* Felippe-Bauer”, same data as allotype (CCER); 1 male, on microscope slide, labeled “Paratype *Downeshelea oliveirai* Felippe-Bauer”, same data as allotype except “June 1970” (CCER); 2 females, on microscope slide, labeled “Paratype *Downeshelea oliveirai* Felippe-Bauer”, same data as allotype except “July 1970” (CCER); 6 males, 3 females, on microscope slide, same data as holotype (2 males, 1 female CNCI; 4 males, 2 females CCER); 3 females, on microscope slide, same data as allotype (CCER); 1 female, on microscope slide, same data as allotype except “June 1970”(CCER).

**Distribution.** This species is known only from Brazil (Pará and Rondônia) in humid forested areas, occurring from 15 to 350 m above sea level (Figure 39).

#### 3.4.28. *Downeshelea panamensis* (Lane and Wirth)

**Figure 32:**
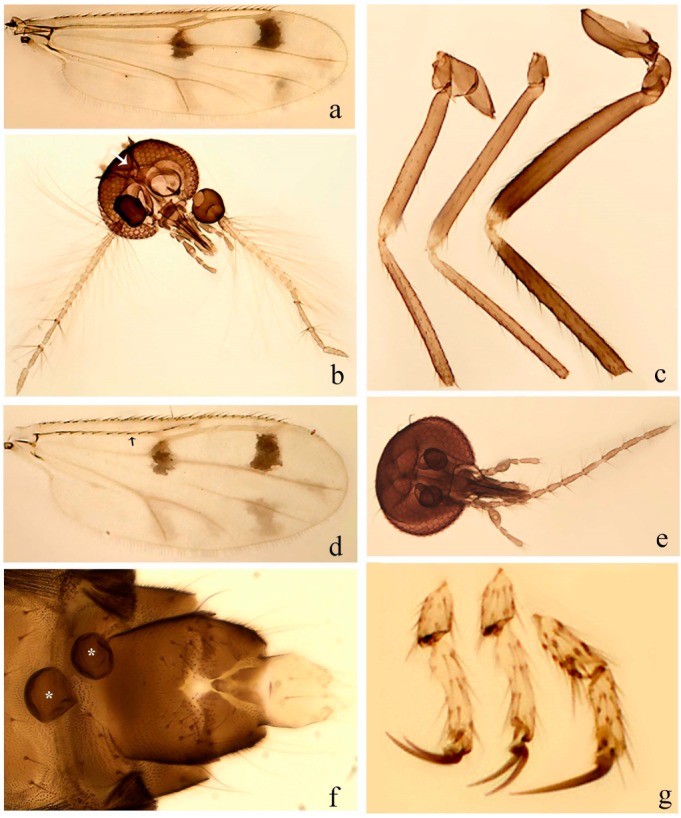
*Downeshelea panamensis*, male: (**a**) wing; (**b**) head, anterior view; arrow: eyes separation; (**c**) fore-, mid-, hind legs (left to right), lateral view. Female: (**d**) wing; arrow: bristles on radius; (**e**) head, anterior view; (**f**) apex of abdomen, ventral view; asterisks: spermathecae; (**g**) fore-, mid-, hind legs claws (left to right), lateral view.

**Figure 33:**
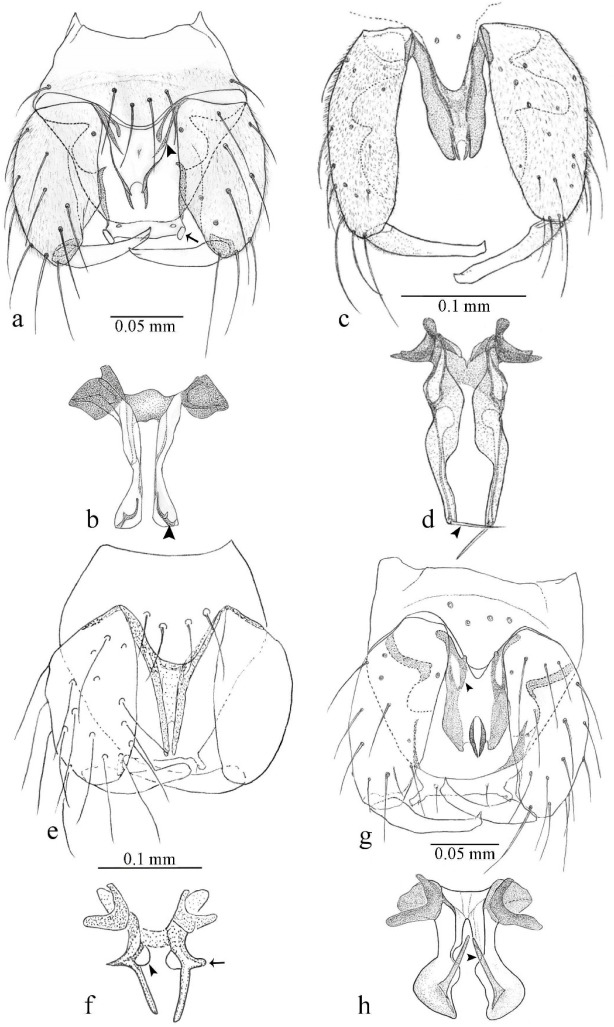
*Downeshelea kuna* sp. nov., male: (**a**) terminalia with aedeagus, ventral view; arrowhead: sclerotized area; (**b**) parameres, ventral view; arrowhead: distal portion. *Downeshelea lanei*, male: (**c**) terminalia with aedeagus, ventral view; (**d**) parameres, ventral view; arrowhead: distal portion. *Downeshelea oliveirai*, male: (**e**) terminalia with aedeagus, ventral view; (**f**) parameres, ventral view; arrowhead: ventral lobe, arrow: median process. *Downeshelea panamensis*, male: (**g**) terminalia with aedeagus, ventral view; arrowhead: sclerotized area; (**h**) parameres, ventral view; arrowhead: subapical process.

Figure 32a–g, Figure 33g,h, and Figure 39; Table 1.

*Monohelea panamensis* Lane and Wirth, 1964 [6]: 221 (male, female; Panama, Mexico, Virgin Islands); Wirth, 1974 [3]: 41 (in catalog south of the USA; distribution).

*Downeshelea panamensis*: Wirth and Grogan, 1988 [7]: 51 (combination); Borkent and Wirth, 1997 [24]: 98 (in World catalog); Borkent and Spinelli, 2000 [25]: 47 (in catalog south of the USA; distribution); Borkent and Spinelli, 2007 [26]: 80 (in Neotropical catalog; distribution); Borkent, 2016 [22]: 124 (in World catalog).

**Diagnosis**. Male: only species of *Downeshelea* in the Americas with the following combination of character states: r_3_ without apical grayish spot (Figure 32a); legs brown, hind femur slightly darker subapically (Figure 32c); paramere nearly straight on basal ½, broadly expanded distally forming a large lobe (Figure 33h); subapical process straight, directed anteriorly (Figure 33h); aedeagus rectangular, with two large elliptical sclerotized anterior areas (Figure 33g). Female: only species of *Downeshelea* in the Americas with the following combination of character states: wing (Figure 32d) and legs as described for male; hind leg claw 1.07–1.25 as long as tarsomere 5 (Figure 32g); slightly unequal spermathecae (Figure 32f).

**Redescription. Male. Head** (Figure 32b). Eyes separated dorsomedially by 2× width of an ommatidium (Figure 32b). Antenna pale, except basal portion of flagellomere 1, distal portion of flagellomere 10, flagellomeres 11–13 brown; antennal ratio 0.90–1.00 (0.97, n = 12). Palpus with segment 3 slightly swollen on midportion with broad, deep sensory pit; palpal ratio 2.40–3.25 (2.60, n = 14). **Thorax**. Scutum brown, without definite pattern in slide mounted specimens; pleura pale brown. Wing (Figure 32a) with three grayish areas: first near apex of M_1_ not abutting wing margin; second sigmoid-shaped extending from m_1_ to wing margin in m_2_; third over CuA_1_, CuA_2_ extending from mediocubital fork into cua_1_, anal cell, reaching wing margin; 2nd radial cell twice length of 1st; length 0.90–1.15 (1.03, n = 14) mm; width 0.32–0.40 (0.36, n = 14) mm; costal ratio 0.72–0.78 (0.75, n = 14). Halter pale brown, distal portion of knob darker. Legs (Figure 32c) brown, hind leg darker, hind femur slightly darker subapically; hind tibial comb with six spines. Foretarsomere 1 with one basal, one apical spine; midtarsomere 1 with 2–3 ventral spines; apical spines of tarsomeres 2–4 of fore-, mid-, hind legs: 2-2-3, 2-2-4, 1-1-3; foretarsal ratio 2.15–2.38 (2.23, n = 13), midtarsal ratio 2.38–2.53 (2.47, n = 14), hind tarsal ratio 2.07–2.26 (2.14, n = 14); claws 0.33–0.46 (0.40, n = 14) length of their respective tarsomere 5. **Abdomen**. Brown. Terminalia (Figure 33g): tergite 9 with quadrate apex, apicolateral process short; sternite 9 concave anteriorly, posterior margin with large convex median lobe bearing 3–4 long setae. Gonocoxite brown, moderately stout, 2.12–2.42 (2.32, n = 14) times longer than basal width; gonostylus brown, nearly straight, 0.60–0.67 (0.63, n = 14) length of gonocoxite. Parameres (Figure 33h) 1.13–1.31 (1.21, n = 14) length of aedeagus, fused on basal portion for 0.15–0.20 (0.18, n = 14) of total length, each with basal arm trilobed, knob bulbous, anteromedian fusion curved basally in some specimens; stem nearly straight on basal ½, broadly expanded distally, forming large lobe, with subapical slender, straight process, directed anteriorly (Figure 33h), 0.28–0.42 (0.36, n = 13) of total length. Aedeagus (Figure 33g) rectangular, heavily sclerotized laterally, basal arch somewhat V-shaped, sclerotized, extending to 0.30–0.41 (0.34, n = 14) of total length, with two large elliptical sclerotized anterior areas (Figure 33g); distal portion with deep mesal notch and two heavily sclerotized pointed processes.

**Female**. Similar to male with usual sexual differences; antenna brown; antennal ratio 1.12–1.19 (1.16, n = 7); palpal ratio 2.17–2.75 (2.37, n = 8) (Figure 32e); mandible with 8–10 teeth. Wing as in Figure 32d; length 0.97–1.17 (1.04, n = 8) mm; width 0.40–0.47 (0.43, n = 8) mm; costal ratio 0.77–0.80 (0.79, n = 8). Foretarsomere 1 with one basal, one apical spine; midtarsomere 1 with 2–5 ventral spines; apical spines of tarsomeres 2–4 of fore-, mid legs: 3-3-3, 2-2-4; foretarsal ratio 2.17–2.49 (2.30, n = 7), midtarsal ratio 2.38–2.73 (2.51, n = 8), hind tarsal ratio 2.19–2.47 (2.33, n = 8); fore-, mid legs claws 0.56–0.68 (0.63, n = 7) length of their respective tarsomeres 5; hind leg claw 1.07–1.25 (1.18, n = 8) as long as tarsomere 5 (Figure 32g). Two slightly unequal spermathecae (Figure 32f), measuring 47–57 (52, n = 7) by 37–50 (45, n = 7) µm and 40–52 (47, n = 7) by 30–47 (40, n = 8) µm. Third rudimentary spermatheca measuring 6.7 µm (not visible in Figure 32f).

**Specimens examined.** 1 female, on microscope slide, labeled “Allotype *Monohelea panamensis* Lane and Wirth”, “Panama, Patino Point Pan Darien Prov, 12 July 1952, light trap, FS Blanton col.” (USNM); 1 male, on microscope slide, labeled “Paratype *Monohelea panamensis* Lane and Wirth”, same data except “13 July 1952” (USNM); 1 male, 2 females, on microscope slide same data except “16 August 1952” (1 male FSP; 2 females USNM); 2 males, 2 females, pinned, labeled “Paratype *Monohelea panamensis* Lane and Wirth”, same data except “17 July 1952”(FSP); “1 April 1952” (FSP); 1 male, on microscope slide, labeled “Paratype *Monohelea panamensis* Lane and Wirth”, “VIRGIN ISLANDS (USA), St. John, Rendezvous Bay, 21 August–2 April 1961, emergence trap, RW Williams col.” (USNM); 1 male, on microscope slide, labeled “Paratype *Monohelea panamensis* Lane and Wirth”, “Mexico, 8 mi. E. Chilpancingo, Guerrero, 17 August 1962, N Marston col.” (USNM); 1 male, on microscope slide, “Colombia, Rio Raposo, VIII.1963, light trap, VH Lee col.” (USNM) (NEW RECORD); 6 males, 8 females, on microscope slide, same data except: “DECEMBER 1963” (1 female CCER, 3 female USNM); “23 March 1964” (1 female USNM); “V.1964 “(1 female USNM); “10 June 1964” (1 male CCER; 4 males USNM) ; “15 June 1964” (1 male, 1 female USNM); March 1965” (1 female USNM); 1 male, on microscope slide, “Costa Rica, Guanacaste, Nadayure, Bejuco, Playa Caletas mangrove, 5 m, 8–12 July 2005, malaise, Gamboa, Gutiérrez, Moraga, Azofeifa and Cárdenas cols.” (MNCR) (NEW RECORD); 1 male, on microscope slide, “Guanacaste, Cuajiniquil, mangrove, 5 m, 13–16 June 2004, instersección trap, Gamboa, Briceno, Moraga and Cárdenas cols.” (MNCR); 1 male, 1 female, on microscope slide, same data except “Playa Ostional, malaise”(MNCR); 1 male, 1 female, on microscope slide, “Guanacaste, Nosara, R. Privada Nosada, Rio Nosara, 5 m, 15 June 2004, red de barrido, Moraga col.” (CCER); 1 male, on microscope slide, same data except “Desembocadura Rio Nosara, 13–17 June 2004, malaise, Gamboa, Briceno, Moraga and Cárdenas cols.” (MNCR); 1 male, on microscope slide, “Limón, Laguna Gandoca, 0–50 m, 17–23 May 2004, malaise, Porras, Gamboa, Briceno, Moraga and Cárdenas cols.” (MNCR); 1 female, on microscope slide, “R.B. Hitoy Cerere, Send. Espavel, 560 m, 21 June–8 July 2003, malaise, Gamboa, Rojas, Arana cols. (MNCR); 1 female, on microscope slide, “Parque Nacional Cahuita, Sector Puerto Vargas, 5 m, 15 October–14 November 2002, malaise, Rojas col. (MNCR); 1 male, on microscope slide, “Puntarenas, 3 km N Caldera, 24 August 1993, Borkent col”. (MNCR); 1 male, on microscope slide, same data except “14 December 1993” (MNCR).

**Distribution and bionomics.** This species is known from Mexico (Guerrero), Virgin Islands (USA), Costa Rica (Guanacaste, Puntarenas and Limón), Panama and Colombia (Figure 38). It occurs in coastal areas, including seaboard and mangrove environments, and forested areas. It has been found at sea level in Colombia and Virgin Islands, up to 50 m in Costa Rica and at 1250 above sea level in Mexico.

**Taxonomic discussion.***Downeshelea panamensis* is similar to species in the *multilineata* group in having a wing pattern of grayish spots and brown legs without bands. It differs from this group, in having paramere with a subapical process similar to those of *D. chiapasi* and *D. colombiae*. *Downeshelea panamensis* differs from these two species by the brownish body coloration and legs without dark bands (yellowish-brown body, legs with dark bands in *D. chiapasi* and *D. colombiae*), the paramere expanded distally forming large lobe and the aedeagus with elliptical sclerotized anterior areas (paramere without distal lobe, aedeagus without elliptical sclerotized areas in *D. chiapasi* and *D. colombiae*). We studied the female allotype and the paratypes specimens from Panama, Mexico, and Virgin Islands deposited in FSP and USNM. The holotype is a pinned specimen from Panama deposited in the USNM (#66440) and no further observations were made of it.

#### 3.4.29. *Downeshelea pulla* sp. nov.

**Figure 34:**
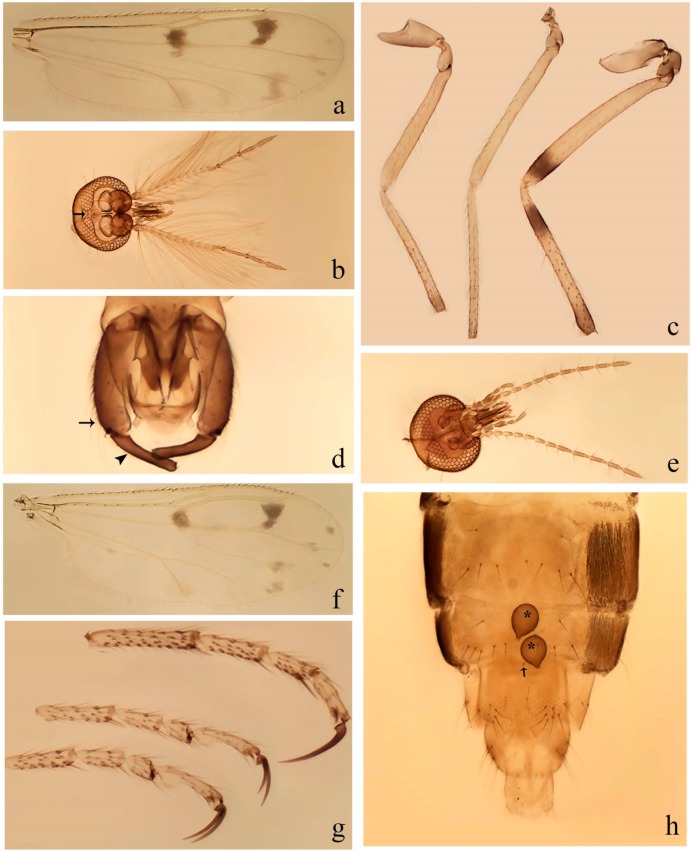
*Downeshelea pulla* sp. nov., male: (**a**) wing; (**b**) head, anterior view; arrow: eyes separation; (**c**) fore-, mid-, hind legs (left to right), lateral view; (**d**) terminalia, ventral view; arrow: gonocoxite; arrowhead: gonostylus. Female: (**e**) head, anterior view; (**f**) wing; (**g**) fore-, mid-, hind legs claws (top to bottom), lateral view; (**h**) apex of abdomen, ventral view; asterisks: spermathecae; arrow; 3rd rudimentary spermatheca.

Figure 34a–h, Figure 37a,b, and Figure 39; Table 1.

*Monohelea chirusi:* Lane and Wirth, 1964 [6]: 218 (male, female; in part, Panama specimens).

**Diagnosis**. Male: only species of *Downeshelea* in the Americas with the following combination of character states: r_3_ with apical grayish spot (Figure 34a); legs yellowish brown, hind femur with subapical, hind tibia with subbasal, apical dark band (Figure 34c); gonostylus dark brown (Figure 34d); paramere stem sinuous (Figure 37b); distal portion short, bent, directed posteromesally (Figure 37b). Female: only species of *Downeshelea* in the Americas with the following combination of character states: wing (Figure 34f) and legs as described for male; hind leg claw 1.33–1.44 as long as tarsomere 5 (Figure 34g); slightly unequal dark brown spermathecae (Figure 34h).

**Description. Male. Head** yellowish (Figure 34b). Eyes separated dorsomedially by 2× width of an ommatidium (Figure 34b). Antenna yellowish, except base of flagellomere 1, distal portion of flagellomere 10, flagellomeres 11–13 brown; antennal ratio 0.93–0.99 (0.97, n = 13). Palpus pale brown with segment 3 slightly swollen on midportion with broad, deep sensory pit; palpal ratio 2.00–2.75 (2.35, n = 13). **Thorax**. Scutum brown without definite pattern in slide mounted specimens; pleura pale brown. Wing (Figure 34a) with six grayish areas: three in apical portion of r_3_, m_1,_ M_1_, arranged in a triangle, not reaching wing margin; one sigmoid-shaped extending from m_1_ to wing margin in m_2_; a small one, faint, in cua_1_ near CuA_1_ not reaching wing margin; one over CuA_2_ extending into cua_1_, anal cell, reaching wing margin; 2nd radial cell twice length of 1st; length 1.02–1.22 (1.14, n = 13) mm; width 0.35–0.40 (0.37, n = 13) mm; costal ratio 0.74–0.80 (0.77, n = 13). Halter pale, distal portion of knob darker. Legs (Figure 34c) yellowish brown, hind femur with subapical, hind tibia with subbasal, apical dark band; hind tibial comb with seven spines. Foretarsomere 1 with one basal, two apical spines; midtarsomere 1 with 2–3 ventral spines; apical spines of tarsomeres 2–4 of fore-, mid-, hind legs: 2-2-2, 2-2-3, 1-1-2; foretarsal ratio 2.14–2.50 (2.30, n = 13), midtarsal ratio 2.40–2.65 (2.52, n = 12), hind tarsal ratio 2.03–2.27 (2.18, n = 13); claws 0.33–0.50 (0.40, n = 13) length of their respective tarsomere 5. **Abdomen**. Yellowish dorsally, brown ventrally. Terminalia brown (Figure 34d and Figure 37a): tergite 9 with quadrate apex, apicolateral process long, slender; sternite 9 straight anteriorly, posterior margin with short convex median lobe bearing 2–3 long setae. Gonocoxite uniformly dark brown (Figure 34d), slender, 2.67–3.15 (2.89, n = 13) times longer than basal width; gonostylus uniformly dark (Figure 34d), straight, 0.53–0.69 (0.60, n = 13) length of gonocoxite. Parameres (Figure 37b) 1.02–1.24 (1.08, n = 12) length of aedeagus, fused on basal portion for 0.12–0.16 (0.14, n = 10) of total length, each with trilobed basal arm, knob slender; stem sinuous, distal third tapering; distal portion (Figure 37b) short, slender, bent, curved mesad, directed posteromesally, only mesally directed in some specimens, tapering to pointed tip, 0.20–0.24 (0.21, n = 12) of total length. Aedeagus (Figure 37a) rectangular, sclerotized, with pair of admedian heavily sclerotized processes (Figure 37a) extending from basal arch to distal portion, basal arch U-shaped, extending to 0.48–0.54 (0.51, n = 13) of total length; distal portion with deep mesal notch and two slender, heavily sclerotized, pointed processes.

**Female**. Similar to male with usual sexual differences; antenna brown, basal portion of flagellomeres pale; antennal ratio 1.05–1.16 (1.09, n = 10); palpal ratio 2.40–2.60 (2.44, n = 9) (Figure 34e); mandible with 10–11 teeth. Wing as in Figure 34f; length 1.15–1.40 (1.28, n = 10) mm; width 0.47–0.55 (0.49, n = 10) mm; costal ratio 0.80–0.85 (0.81, n = 10). Foretarsomere 1 with one basal, two apical spines; midtarsomere 1 with 2–4 ventral spines; apical spines of tarsomeres 2–4 of fore-, mid legs: 2-2-2, 2-2-3; foretarsal ratio 2.32–2.59 (2.46, n = 10), midtarsal ratio 2.52–2.86 (2.66, n = 10), hind tarsal ratio 2.41–2.65 (2.52, n = 10); fore-, mid legs claws 0.70–0.79 (0.74, n = 9) length of their respective tarsomeres 5; hind leg claw 1.33–1.44 (1.36, n = 9) as long as tarsomere 5 (Figure 34g). Two slightly unequal dark brown spermathecae (Figure 34h), measuring 53–63 (58, n = 10) by 43–50 (46, n = 6) µm and 48–55 (51, n = 10) by 40–50 (43, n = 7) µm. Third rudimentary spermatheca measuring 8.2 µm (Figure 34h).

**Specimens examined.** Holotype male, on microscope slide, labeled “Holotype *Downeshelea pulla* Santarém, Borkent and Felippe-Bauer”, “*Downeshelea chirusi* (Lane and Wirth)”, “Belize, Hattieville, 8 July 1968, light trap, Haase col.” (CCER); allotype female, on microscope slide, labeled “Allotype *Downeshelea pulla* Santarém, Borkent and Felippe-Bauer”, “*Downeshelea chirusi* (Lane and Wirth)”, same data as holotype except “7–8 July 1968, black light” (CCER). Paratypes labeled as follows: 17 males, on microscope slide, same data as holotype (13 USNM, 4 CCER); 18 females, on microscope slide, same data as allotype (14 USNM, 4 CCER); 1 female, on microscope slide, same data as allotype except “Columbia Forest, July 1968”; 1 female, on microscope slide, labeled “Allotype, *Monohelea chirusi* Lane and Wirth”, “PANAMÁ, Coclé Prov., Puerto Obaldia, 11 November 1952, light trap, Blanton col.” (USNM); 2 females, pinned, labeled “Paratype, *Monohelea chirusi* Lane and Wirth”, same data (USNM; FSP); 1 male, pinned, with genitalia in drop of Canada Balsam on celluloid strip on pin, labeled “Paratype, *Monohelea chirusi* Lane and Wirth”, same data except “Aguadulce, 25 September 1951” (FSP); 1 female, pinned, labeled “Paratype, *Monohelea chirusi* Lane and Wirth”, same data except “Almirante, Bocas del Toro, I. 1953” (FSP); 1 male, 1 female, on microscope slide, “Costa Rica, Guanacaste, Ostional, Rio Montana, 100 m, 13 June 2004, light trap, Gamboa, Briceno, Moraga and Cardenas cols.” (MNCR); 1 male, 2 females, on microscope slide, “Guanacaste, Bagaces, Parque Nacional Palo Verde, Sector Palo Verde, 0–50 m, 6 October–8 November 1999, Malaise, Jiménez col.” (CNCI); 1 male, on microscope slide, same data except Puerto Chamorro, 0–10 m, 15 November 2004, red de barrido, Gamboa col.” (MNCR); 1 female, on microscope slide, “Guanacaste, Abangares, Cerros de Naranjo, 100 m, 12 November 2004, light trap, Porras, Gamboa, Moraga and Cardenas cols.”(MNCR); 2 males, 2 females, on microscope slide, same data except “Camino del ICE, 200 m, 11 November 2004 (1 male, 1 female CCER; 1 male, 1 female MNCR); 3 males, 3 females, on microscope slide, “Guanacaste, La Cruz, Parque Nacional Santa Rosa, Sector Murciélago, 5.5 km ENE del C. Guachipelín, 40 m, 29 June–27 July 1996, Malaise, Araya col.” (MNCR); 3 males, 1 female, on microscope slide, same data except “27 July–27 August 1996” (MNCR).

**Distribution and bionomics.** This species is known from Belize, Costa Rica (Guanacaste) and Panamá (Figure 39). It has been collected from forested and coastal areas, occurring from sea level (Panamá) to 300 m above sea level (Belize and Costa Rica).

**Etymology**. This species name refers to its dark body coloration that differs from the similar but yellowish species *D. chirusi*. (Latin—pulla = dark).

**Taxonomic discussion.** This species is similar to *D. chirusi* by virtue of the pattern of dark spots on the wing, leg coloration and the general aspect of male genitalia. However, *D. chirusi* has a yellowish terminalia, only with distal halves of gonocoxite and gonostylus dark and pale spermathecae, while *D. pulla* sp. nov. has a uniformly dark brown terminalia and dark spermathecae. In both species, the male paramere are expanded medially, but in *D. chirusi* they are broader than in *D. pulla*. Furthermore, there are slight variations of some meristic data as follows: male antennal ratio, length/width of gonocoxite and female hind claws (Table 1). *Downeshelea chirusi* is known from Mexico, Nicaragua (?), Costa Rica and Panama and *D. pulla* occurs in Belize, Costa Rica and Panama. Both species occurs in sympatry in Panama at Puerto Obaldia and Almirante, which are tropical humid areas. In Costa Rica these species are present at different elevations and provinces: *D. chirusi* is found in six of seven provinces up to 1210 m above sea level and is not present in Guanacaste (Figure 21), while *D. pulla* is only present in Guanacaste up to 200 m above sea level (Figure 39). Although Guanacaste is distinct from other provinces because of a severe dry season, the specimens of *D. pulla* were only collected from June to November, during the rainy season.

#### 3.4.30. *Downeshelea quasidentica* Felippe-Bauer and Quintelas

**Figure 35:**
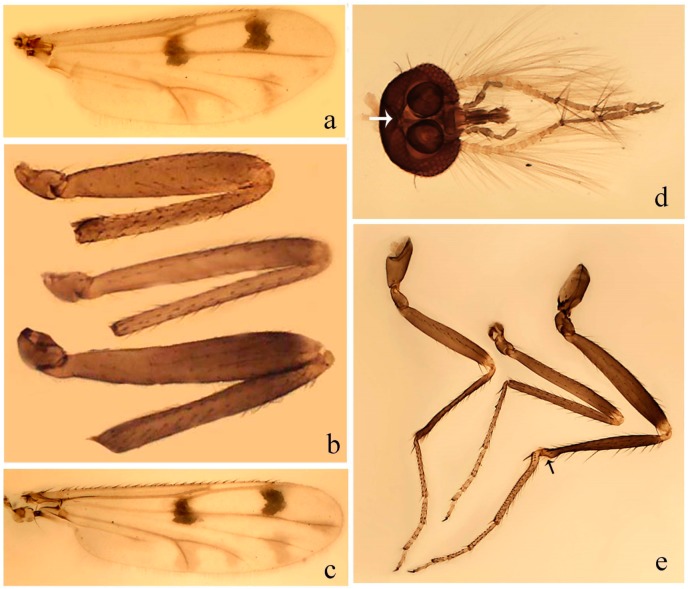
*Downeshelea quasidentica*, male: (**a**) wing; (**b**) fore-, mid-, hind legs (top to bottom), lateral view. *Downeshelea quechua* sp. nov., male: (**c**) wing; (**d**) head, anterior view; arrow: eyes separation (**e**) fore-, mid-, hind legs (left to right), lateral view; arrow: hind tibia apex.

Figure 35a,b, Figure 37c–e, and Figure 39; Table 1.

*Downeshelea quasidentica* Felippe-Bauer and Quintelas, 1993 [16]: 37 (male, Brazil); Borkent and Wirth, 1997 [24]: 98 (in World catalog); Borkent and Spinelli, 2000 [25]: 47 (in catalog south of the USA; distribution); Borkent and Spinelli, 2007 [26]: 81 (in Neotropical catalog; distribution); Borkent, 2016 [22]: 124 (in World catalog); Santarém and Felippe-Bauer, 2019 [2]: 17 (Brazilian distribution).

**Diagnosis.** Male: only species of *Downeshelea* in the Americas with the following combination of character states: r_3_ without apical grayish spot (Figure 35a); legs brown, hind femur darker subapically, hind tibia darker subbasally (Figure 35b); parameres separated, stem sinuous; distal portion as membranous lobe (Figure 37e); aedeagus quadrangular (Figure 37d), with two sclerotized horn-like process medially (Figure 37d). Female unknown.

**Specimens examined.** 1 male, on microscope slide, labeled “Holotype *Downeshelea quasidentica* Felippe-Bauer and Quintelas”, “Brazil, Rio de Janeiro, São Pedro da Aldeia, Estação, Rua Coronel F. Pinheiro, 43 (−22.839199 S, −42.102798 W), 27 March 1989” (CCER); 1 male, on microscope slide, labeled “Paratype *Downeshelea quasidentica* Felippe-Bauer and Quintelas”, same data as holotype (CCER).

**Distribution.** This species is known only from Brazil (Rio de Janeiro) in an urban humid area at 10 m above sea level (Figure 39).

#### 3.4.31. *Downeshelea quechua* sp. nov.

Figure 35c–e, Figure 37f,g, and Figure 39; Table 1.

**Diagnosis**. Male: only species of *Downeshelea* in the Americas with the following combination of character states: r_3_ without apical grayish spot (Figure 35c); legs brown, all femora darker subapically (Figure 35e), hind tibia pale apically (Figure 35e); paramere stem broad basally, slightly divergent in distal ½ (Figure 37g); distal portion ending in two process externally curved, the inner one short, claw-shaped (Figure 37g), outer one spirally (Figure 37g); aedeagus subrectangular (Figure 37f), distal portion with dorsal sclerotized lobe (Figure 37f). Female unknown.

**Description. Male**. **Head** (Figure 35d). Eyes separated dorsomedially by 2.5× width of an ommatidium (Figure 35d). Antenna pale, except basal portion of flagellomere 1, distal portion of flagellomere 10, flagellomeres 11–13 brown; antennal ratio 0.97. Palpus with segment 3 slightly swollen on midportion with broad, deep sensory pit; palpal ratio 2.00.

**Thorax**. Scutum brown, postscutellum darker, pleura pale brown. Wing (Figure 35c) with three pale grayish areas: first near apex of M_1_, not reaching wing margin; second sigmoid-shaped extending from m_1_ to wing margin in m_2_; third over CuA_1,_ CuA_2_ extending into cua_1,_ anal cell, reaching wing margin in CuA_2_; 2nd radial cell twice length of 1st; length 1.10 mm; width 0.35 mm; costal ratio 0.77. Halter pale, distal portion of knob darker. Legs (Figure 35e) brown, hind leg darker, all femora darker subapically, hind tibia pale apically (Figure 35e); hind tibial comb with six spines. Foretarsomere 1 with one basal, one apical spine; midtarsomere 1 without ventral spines; apical spines of tarsomeres 2–4 of fore-, mid-, hind legs: 3-3-4, 3-3-4, 1-1-2; foretarsal ratio 2.38; midtarsal ratio 2.28; hind tarsal ratio 2.33; claws 0.42 length of their respective tarsomere 5. **Abdomen**. Brown. Terminalia (Figure 37f): tergite 9 with quadrate apex, apicolateral process short; sternite 9 straight anteriorly, posterior margin with large convex median lobe bearing three long setae. Gonocoxite brown, moderately stout, 2.10 times longer than basal width; gonostylus brown, nearly straight, 0.75 length of gonocoxite. Parameres (Figure 37g) 1.02 length of aedeagus, fused on basal portion for 0.09 of total length, each with basal arm trilobed, knob flattened; stem sinuous, broad basally, slightly divergent in distal ½, tapering distally; distal portion ending in two process externally curved, the inner one (Figure 37g) short, claw-shaped, the outer one (Figure 37g) spirally. Aedeagus (Figure 37f) subrectangular, sclerotized laterally near base (Figure 37f); basal arch U-shaped, heavily sclerotized, extending to 0.26 of total length; distal portion (Figure 37f) with deep mesal notch, dorsal lobe sclerotized, two strong sclerotized slightly serrate processes.

**Female**. Unknown.

**Specimens examined.** Holotype male, on microscope slide, labeled “Holotype *Downeshelea quechua* Santarém, Borkent and Felippe-Bauer”, “Bolivia, Carrasco National Park entrance, 12 km SW Villa Tunari, 484 m, (65°28.53′ W; 17°23.89′ S), 17 December 2016, A. Borkent col.” (MNHN).

**Distribution and bionomics.** This species is known only from Bolivia (Figure 39) in mature rainforest at 484 m above sea level.

**Etymology.** This species name refers to the Quechua language that is spoken by indigenous Quechua peoples in Bolivia.

**Taxonomic discussion.** The male of *Downeshelea quechua* most closely resembles *D. bifida* and *D. divergentis* in having apices of the parameres divergent with bifid distal portion, but *D. quechua* can be easily distinguished by the distal portion of the paramere with outer process spirally (outer process tapering to pointed tip in *D. bifida*; outer process as a rounded lobe in *D. divergentis*) and the aedeagus with distal sclerotized lobes (aedeagus without distal lobes in both species). Other characters to distinguish the species are in the taxonomic discussion of *D. bifida*.

#### 3.4.32. *Downeshelea rodriguezi* sp. nov.

**Figure 36:**
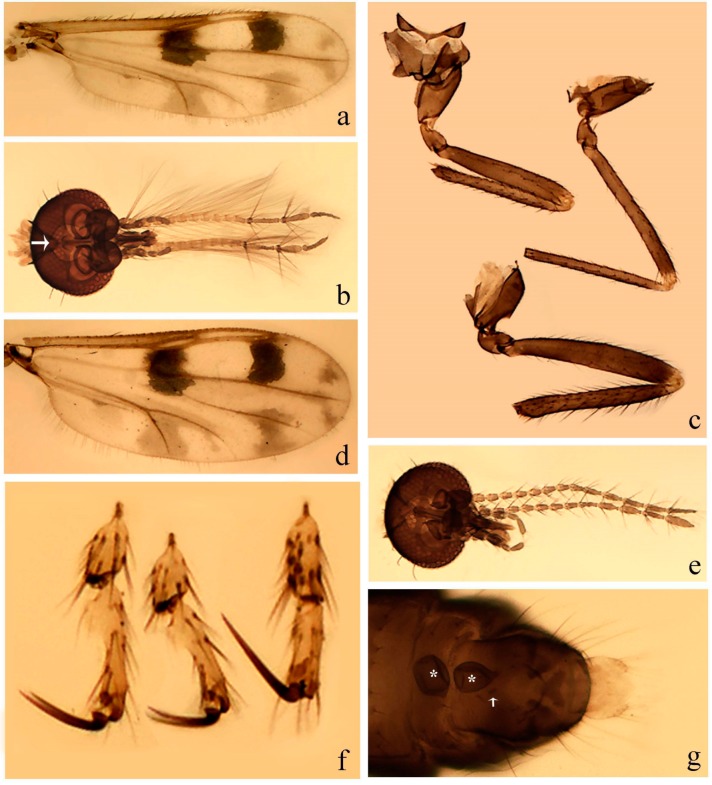
*Downeshelea rodriguezi* sp. nov., male: (**a**) wing; (**b**) head, anterior view; arrow: eyes separation; (**c**) fore-, mid-, hind legs (top to bottom), lateral view. Female: (**d**) wing; (**e**) head, anterior view; (**f**) fore-, mid-, hind legs claws (left to right), lateral view; (**g**) apex of abdomen, ventral view; asterisks: spermathecae; arrow: 3rd rudimentary spermatheca.

**Figure 37:**
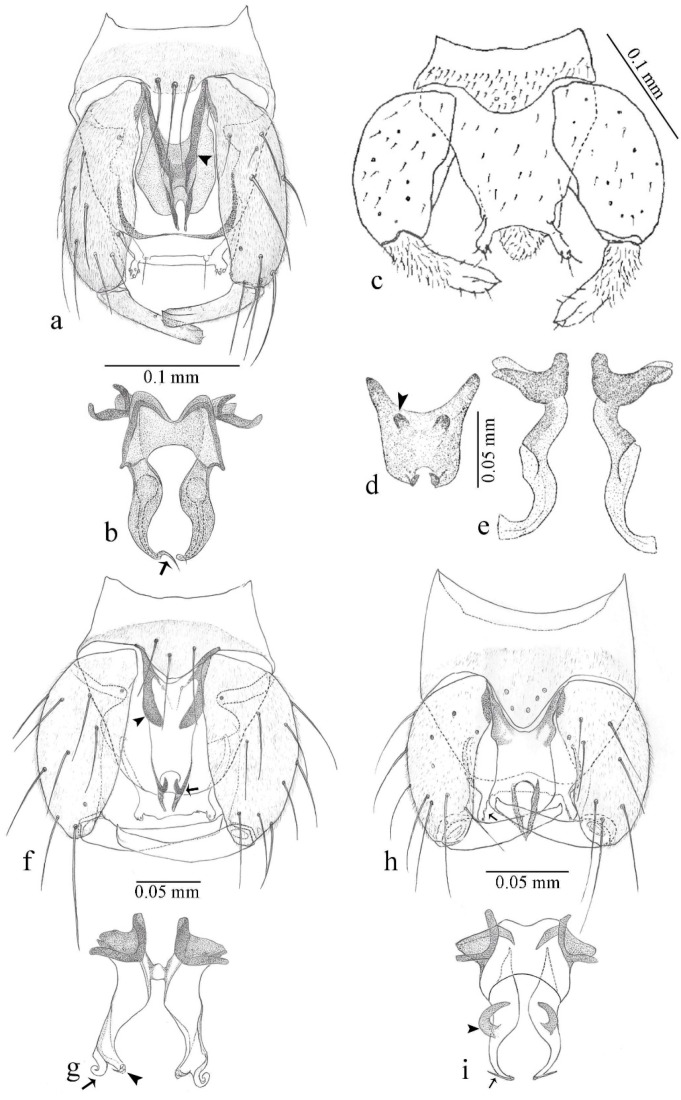
*Downeshelea pulla* sp. nov., male: (**a**) terminalia with aedeagus, ventral view; arrowhead: sclerotized process; (**b**) parameres, ventral view; arrow: distal portion. *Downeshelea quasidentica*, male: (**c**) terminalia, ventral view; (**d**) aedeagus, ventral view; arrowhead: horn-like process; (**e**) parameres, ventral view. *Downeshelea quechua* sp. nov., male: (**f**) terminalia with aedeagus, ventral view; arrowhead: aedeagus lateral sclerotized area; arrow: aedeagus distal portion; (**g**) parameres, ventral view; arrowhead: distal portion inner process; arrow: distal portion outer process. *Downeshelea rodriguezi* sp. nov., male: (**h**) terminalia with aedeagus, ventral view; arrow: tergite 9 apicolateral process; (**i**) parameres, ventral view; arrowhead: median process; arrow: distal portion.

Figure 36a–g, Figure 37h,i, and Figure 39; Table 1.

**Diagnosis**. Male: only species of *Downeshelea* in the Americas with the following combination of character states: wing with extensive dark markings; r_3_ with apical grayish spot (Figure 36a); legs brown, hind tibia slightly darker subbasally, apically (Figure 36c); paramere midportion with stout, curved, horn-like process, the right one slightly greater than left (Figure 37i); distal portion with sharp point, externally directed (Figure 37i); aedeagus rectangular (Figure 37h). Female: only species of *Downeshelea* in the Americas with the following combination of character states: wing (Figure 36d) and legs as described for male; hind leg claw 1.08–1.27 as long as tarsomere 5 (Figure 36f); equal-sized spermathecae (Figure 36g).

**Description. Male**. **Head** (Figure 36b). Eyes separate dorsomedially by width of one ommatidium (Figure 36b). Antenna pale, except basal portion of flagellomere 1, distal portion of flagellomere 10, flagellomeres 11–13 brown; antennal ratio 0.87–0.89 (0.88, n = 2). Palpus with segment 3 swollen on midportion with broad, deep sensory pit; palpal ratio 2.00–2.25 (2.13, n = 2). **Thorax**. Scutum, postscutellum dark brown, pleura pale brown. Wing (Figure 36a) with five grayish area: three in apical portion of r_3_, m_1,_ M_1,_ reaching wing margin in m_1_; one sigmoid-shaped extending from m_1_ to wing margin in m_2_; one over CuA_1,_ CuA_2_ extending from mediocubital fork into cua_1,_ anal cell, reaching wing margin in CuA_2_; 2nd radial cell twice length of 1st; length 0.90–0.97 (0.94, n = 2) mm; width 0.32–0.35 (0.34, n = 2) mm; costal ratio 0.72 (n = 2). Halter dark brown. Legs (Figure 36c) brown, hind leg darker, hind tibia slightly darker subbasally, apically; hind tibial comb with six spines. Foretarsomere 1 with one basal, one apical spine; midtarsomere 1 with 1–2 ventral spines; apical spines of tarsomeres 2–4 of fore-, mid-, hind legs: 2-2-4, 2-2-4, 1-1-2; foretarsal ratio 2.22–2.29 (2.26, n = 2); midtarsal ratio 2.50–2.53 (2.51, n = 2); hind tarsal ratio 2.12–2.17 (2.14, n = 2); claws 0.40–0.50 (0.45, n = 2) length of their respective tarsomere 5. **Abdomen**. Dark brown. Terminalia (Figure 37h): tergite 9 with quadrate apex, apicolateral process (Figure 37h) very long, slender; sternite 9 slightly concave anteriorly, posterior margin with prominent convex median lobe bearing 4–5 long setae. Gonocoxite brown, moderately stout, 2.10–2.21 (2.15, n = 2) times longer than basal width; gonostylus brown, stout, straight, 0.69–0.74 (0.71, n = 2) length of gonocoxite. Parameres (Figure 37i) 1.11–1.13 (1.12, n = 2) length of aedeagus, fused on basal portion by 0.26–0.28 (0.27, n = 2) of total length, each with basal arm trilobed, knob flattened; stem sinuous, expanded basally, tapering distally; midportion (Figure 37i) with stout, curved, horn-like process, the right one slightly greater than left; distal portion (Figure 37i) bent externally to a short sharp point, 0.14–0.17 (0.15, n = 2) of total length. Aedeagus (Figure 37h) rectangular, basal arch V-shaped, heavily sclerotized, extending to 0.37–0.39 (0.38, n = 2) of total length; distal portion with deep mesal notch and two prominent sclerotized processes.

**Female**. Similar to male with usual sexual differences; antenna brown, basal portion of flagellomeres pale; antennal ratio 1.02 (n = 2); palpal ratio 2.25–2.50 (2.38, n = 2) (Figure 36e); mandible with 9 teeth. Wing as in Figure 36d; length 1.00 (n = 2) mm; width 0.42 (n = 2) mm; costal ratio 0.78 (n = 2). Foretarsomere 1 with one basal, one apical spine; midtarsomere 1 with 2–3 ventral spines; spines of tarsomeres 2–4 of fore-, mid legs: 2-2-3, 2-2-3; foretarsal ratio 2.41–2.47 (2.44, n = 2), midtarsal ratio 2.56–2.59 (2.57, n = 2), hind tarsal ratio 2.38–2.48 (2.43, n = 2); fore-, mid legs claws 0.64–0.66 (0.65, n = 2) length of their respective tarsomeres 5; hind leg claw 1.08–1.27 (1.17, n = 2) as long as tarsomere 5 (Figure 36f). Two equal-sized spermathecae (Figure 36g), measuring 45–50 (48, n = 2) by 35–37 (36, n = 2) µm. Third rudimentary spermatheca measuring 7.5 µm (Figure 36g).

**Specimens examined.** Holotype male, on microscope slide, labeled “Holotype *Downeshelea rodriguezi* Santarém, Borkent and Felippe-Bauer”, “Bolivia, 2 km E Carrasco National Park entrance, 12 km SW Villa Tunari, 517 m (65°29.25′ W; 17°04.28′ S), 16 December 2016, A. Borkent col.” (MNHN). Allotype female labeled “Allotype *Downeshelea rodriguezi* Santarém, Borkent and Felippe-Bauer”, same data as holotype (MNHN). Paratypes labeled as follows: 1 male, 1 female, on microscope slide, same data as holotype (CNCI).

**Distribution and bionomics.** This species is known only from Bolivia (Figure 39). It has been found in secondary rainforest at 517 m above sea level. One male (uncertain whether holotype or paratype) had a pollinium attached to its body, indicating the possibility that this species may pollinate a species of orchid.

**Etymology.** This species name refers to Dr. Jaime Ivan Rodriguez Fernandez in recognition of his entomological contributions, particularly in Bolivia.

**Taxonomic discussion.** This species most closely resembles *D. fuscipennis* in the general body coloration, wing pattern and male paramere with a median horn-like process. Characters for distinguishing both species are in the discussion section of that species.

#### 3.4.33. *Downeshelea spatha* sp. nov.

**Figure 38:**
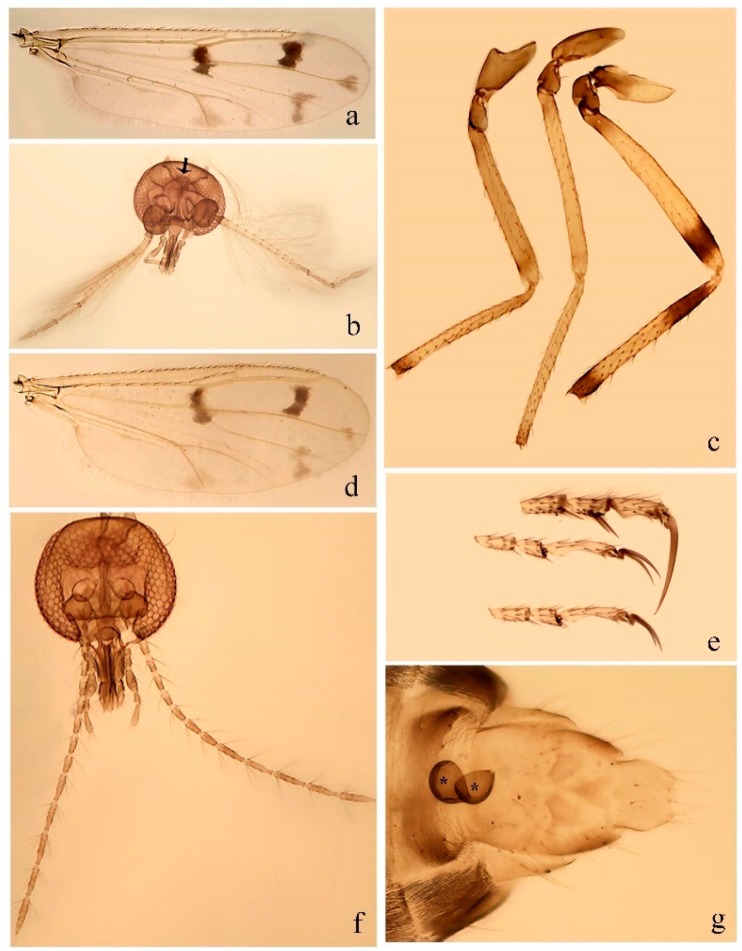
*Downeshelea spatha* sp. nov., male: (**a**) wing; (**b**) head, anterior view; arrow: eyes separation; (**c**) fore-, mid-, hind legs (left to right), lateral view. Female: (**d**) wing; (**e**) fore-, mid-, hind legs claws (top to bottom), lateral view; (**f**) head, anterior view; (**g**) apex of abdomen, ventral view; asterisks: spermathecae.

**Figure 39:**
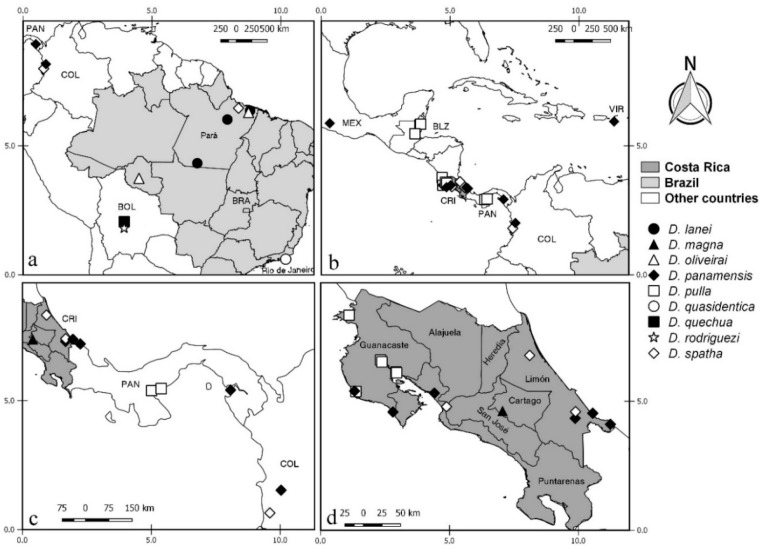
Geographic distribution of *Downeshelea lanei* (**a**), *D. magna* sp. nov. (**c**,**d**), *D. oliveirai* (**a**), *D. panamensis* (**a**,**b**,**c**,**d**), *D. pulla* sp. nov. (**b**,**c**,**d**), *D. quasidentica* (**a**), *D. quechua* sp. nov. (**a**), *D. rodriguezi* sp. nov. (**a**) and *D. spatha* sp. nov. (**a**,**b**,**c**,**d**).

Figure 38a–g, Figure 39, and Figure 43a,b; Table 1.

**Diagnosis**. Male: only species of *Downeshelea* in the Americas with the following combination of character states: r_3_ without apical grayish spot (Figure 38); legs yellowish brown, all femora with dark basal band, fore-, hind femur with subapical dark bands, all tibiae with apical dark band, hind tibia with subbasal dark bands (Figure 38c); gonostylus brown (Figure 43a); paramere stem abruptly tapering distally (Figure 43b); distal portion tapering to pointed tip, directed anteromesally (Figure 43b). Female: only species of *Downeshelea* in the Americas with the following combination of character states: wing (Figure 38d) and legs as described for male; hind leg claw 1.33–1.50 as long as tarsomere 5 (Figure 38e); subequal spermathecae (Figure 38g).

**Description. Male**. **Head** (Figure 38b). Eyes contiguous in lower portion (Figure 38b). Antenna pale, except basal portion of flagellomere 1, distal portion of flagellomere 10, flagellomeres 11–13 brown; antennal ratio 0.98–1.07 (1.02, n = 3). Palpus with segment 3 swollen on midportion with broad, deep sensory pit; palpal ratio 2.00–2.60 (2.39, n = 5). **Thorax**. Scutum yellowish brown, without definite pattern in slide mounted specimens; pleura yellowish brown. Wing (Figure 38a) with three grayish area: first near apex of M_1_, not reaching wing margin; second extending from m_1_ to wing margin in m_2_; third over distal ½ of CuA_2_ slightly extending into cua_1,_ anal cell, reaching wing margin; 2nd radial cell twice length of 1st; length 1.05–1.22 (1.15, n = 5) mm; width 0.37–0.40 (0.38, n = 5) mm; costal ratio 0.75–0.79 (0.77, n = 5). Halter pale, knob brown. Legs (Figure 38c) yellowish brown, faint dark bands in fore-, mid femur basally, fore femur subapically, fore-, midtibia apically; strong dark bands in hind femur basally, subapically, hind tibia subbasally, apically; hind tibial comb with seven spines. Foretarsomere 1 with one basal, one apical spine; midtarsomere 1 with 1–2 ventral spines; apical spines of tarsomeres 2–4 of fore-, mid-, hind legs: 2-2-3, 2-2-3, 1-1-1; foretarsal ratio 2.35–2.58 (2.44, n = 5), midtarsal ratio 2.31–2.52 (2.45, n = 5), hind tarsal ratio 2.12–2.23 (2.15, n = 4); claws 0.38–0.46 (0.42, n = 5) length of their respective tarsomere 5. **Abdomen**. Yellowish dorsally with ventrolateral brown marks on segments 1–7. Terminalia (Figure 43a): pale brown; tergite 9 with quadrate apex, apicolateral process short; sternite 9 straight anteriorly, posterior margin with prominent convex median lobe bearing 3–5 long setae. Gonocoxite brown, slender, 2.61–2.78 (2.70, n = 4) times longer than basal width; gonostylus (Figure 43a) brown, curved, tip blunt, 0.56–0.67 (0.62, n = 5) length of gonocoxite. Parameres (Figure 43b) 1.04–1.13 (1.10, n = 5) length of aedeagus, fused on basal portion by 0.25–0.33 (0.30, n = 5) of total length, each with basal arm trilobed, knob bulbous, anteromedial fusion straight basally; stem broad basally, abruptly tapering distally, distal portion (Figure 43b) abruptly bent, straight, broad, tapering to pointed tip, directed anteromesally, 0.60–0.70 (0.65, n = 5) of total length. Aedeagus (Figure 43a) subrectangular, heavily sclerotized, basal arch U-shaped, extending to 0.32–0.42 (0.36, n = 5) of total length; distal portion with deep mesal notch and two sclerotized, slightly serrate, pointed processes.

**Female**. Similar to male with usual sexual differences; antenna brown; basal portion of flagellomeres paler; antennal ratio 1.08–1.20 (1.13, n = 3); palpal ratio 2.00–2.33 (2.17, n = 5) (Figure 38f); mandible with 9–11 teeth. Wing as in Figure 38d; length 1.12–1.27 (1.22, n = 7) mm; width 0.45–0.53 (0.49, n = 7) mm; costal ratio 0.80–0.84 (0.82, n = 7). Foretarsomere 1 with one basal, one apical spine; midtarsomere 1 with 2–4 (rarely 6) other ventral spines; apical spines of tarsomeres 2–4 of fore-, mid legs: 2-3-4, 2-2-4(3); foretarsal ratio 2.42–2.65 (2.56, n = 7), midtarsal ratio 2.54–2.90 (2.67, n = 7), hind tarsal ratio 2.42–2.72 (2.59, n = 7); fore-, mid legs claws 0.61–0.75 (0.68, n = 7) length of their respective tarsomeres 5; hind leg claw 1.33–1.50 (1.42, n = 7) as long as tarsomere 5 (Figure 38e). Two subequal rounded spermathecae (Figure 38g) measuring 53–63 (58, n = 7) by 45–53 (47, n = 6) µm and 50–60 (53, n = 6) by 38–50 (43, n = 4) µm. Third rudimentary spermatheca measuring 8.2 µm (not visible in Figure 38g).

**Specimens examined.** Holotype male, on microscope slide, labeled “Holotype *Downeshelea spatha* Santarém, Borkent and Felippe-Bauer”, “Costa Rica, Limón, Estación Biológica Hitoy Cerere, Send. Toma de Agua, 100 m, 17 April–8 May 1999, malaise trap, F Umana col.” (MNCR); allotype female, on microscope slide, labeled “Allotype *Downeshelea spatha* Santarém, Borkent and Felippe-Bauer”, “Costa Rica, Limón, Parque Nacional Tortuguero, Agua Fria, Send. Real, 20–50 m, 14–16 August 2004, light trap, Porras, Gamboa, Briceno, Moraga col.” (MNCR). Paratypes labeled as follows: 1 male, 1 female, on microscope slide, same data as holotype (CCER); 1 male, on microscope slide, “Limón, Talamanca, Cahuita, Sector Puerto Vargas, 5 m, 9 January–10 February 2002, red de golpe, E Rojas col.” (MNCR); 1 female, on microscope slide, same data except “9 August–15 October 2002, malaise” (MNCR); 1 female, on microscope slide, same data as holotype except “Valle de la Estrella, 140 m, 17 June–17 July 1999, malaise” (MNCR); 3 females, on microscope slide, “Puntarenas, Garabito, Parque Nacional Carara, Sector Laguna Meandrica, Sítio Quebrada Mona, 100 m, May–June 1990, manual, R Zuniga col.” (MNCR); ); 3 females, on microscope slide, “Brazil, Pará, Belém, APEG forest, February 1970, light trap, THG Aitken col.”(USNM); 1 male, 6 females, on microscope slide, same data except: “April 1970” (2 females USNM); “June 1970” (1 female; USNM); “September 1970” (1 male, 1 female CCER; 2 females USNM); 1 male, on microscope slide, “Colombia, Valle Rio Raposo, April 1965, light trap, VH Lee col.” (USNM); 1 female on microscope slide, same data except August 1965 (USNM)”; 1 female, on microscope slide, labeled “Paratype *Monohelea chiapasi* Lane and Wirth”, same data except “April 1963” (USNM); 2 females on microscope slide, labeled “*Monohelea chiapasi* Lane and Wirth”, same data except “Meta Finca Barbascal, 27–30 April 1964” (USNM).

**Distribution and bionomics.** This species is known from Costa Rica (Puntarenas and Limón), Colombia and Brazil (Pará) (Figure 39). It has been found in forested and coastal areas, occurring from sea level in Colombia, at 15 m in Brazil and in Costa Rica at 20 to 140 m above sea level.

**Etymology**. This species name refers to the similarity of the distal portion of paramere to a dagger. (Latin—spatha = dagger).

**Taxonomic discussion**. *Downeshelea spatha* most closely resembles *D. gladius*. Characters for distinguishing both species from other species with dark bands on the hind legs are in the discussion section of *D. avizi*.

#### 3.4.34. *Downeshelea stonei* (Wirth)

**Figure 40:**
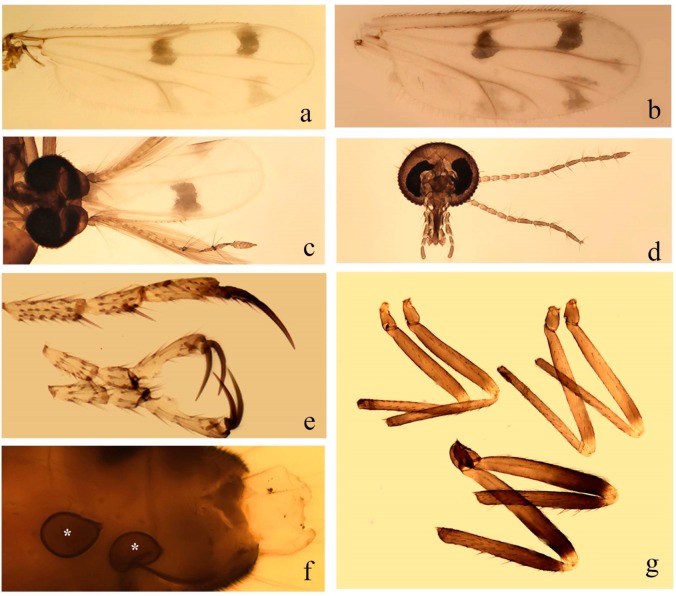
*Downeshelea stonei*, male: (**a**) wing; (**c**) head, anterior view. Female: (**b**) wing; (**d**) head, anterior view; (**e**) fore-, mid-, hind legs claws (bottom to top), lateral view; (**f**) apex of abdomen, ventral view; asterisks: spermathecae; (**g**) fore-, mid-, hind legs (top to bottom), lateral view.

Figure 40a–g, Figure 43c–e, and Figure 46; Table 1.

*Monohelea stonei* Wirth, 1953 [4]: 148 (female, male; USA, Costa Rica and Panamá); Wirth and Williams, 1964 [5]: 303 (Bahamas record); Lane and Wirth, 1964 [6]: 222 (Cuba, Trinidad records; except record for Colombia); Wirth, 1974 [3]: 41 (in catalog south of the USA; distribution); Wirth and Grogan, 1981 [38]: 46 (redescription; distribution).

*Downeshelea stonei*: Wirth and Grogan, 1988: [7] 50 (combination, type species); McKeever et al., 1991 [39]: 95 (male, female mouthparts); Borkent and Wirth, 1997 [24]: 98 (in World catalog); Borkent and Spinelli, 2000 [25]: 47 (in catalog south of the USA; distribution); Borkent and Spinelli, 2007 [26]: 81 (in Neotropical catalog; distribution); Borkent and Grogan, 2009 [33]: 20 (in Nearctic catalog; distribution); Grogan et al., 2010 [34]: 35 (Florida records); Borkent, 2016 [22]: 125 (in World catalog).

**Diagnosis**. Male: only species of *Downeshelea* in the Americas with the following combination of character states: r_3_ without apical grayish spot (Figure 40a); legs brown, fore-, midtibia darker apically, hind femur darker subapically, hind tibia darker subbasally; male paramere stem slightly expanded subapically (Figure 43e), distal portion bent on two directions, broad basally tapering distally to slender pointed tip (Figure 43e); aedeagus rectangular (Figure 43d), two anterolateral curved projections (Figure 43d). Female: only species of *Downeshelea* in the Americas with the following combination of character states: wing (Figure 40b) and legs (Figure 40g) as described for male; hind leg claw 1.27 as long as tarsomere 5 (Figure 40e); slightly unequal spermathecae (Figure 40f).

**Redescription. Male**. **Head** (Figure 40c). Eyes separate (unable to measure; not visible in Figure 40c). Antenna pale, except basal portion of flagellomere 1, distal portion of 10, flagellomeres 11–13 brown; antennal ratio 1.02 (n = 1) (Figure). Palpus with segment 3 slightly swollen on mid portion with palpal pit not visible in slide mounted specimen; palpal ratio 2.60 (n = 1). **Thorax**. Scutum brown, without definite pattern in slide mounted and pinned specimens; pleura pale brown. Wing (Figure 40a) with two grayish areas: one sigmoid-shaped extending from m_1_ to wing margin in m_2_; one over CuA_2_ extending into cua_1_, anal cell, from mediocubital fork to wing margin; faint grayish area in M_1_ not reaching wing margin in some specimens; 2nd radial cell twice length of 1st; length 1.38 (n = 1) mm, width 0.48 (n = 1) mm; costal ratio 0.74 (n = 1). Halter pale, distal portion of knob darker. Legs (as female in Figure 40g) brown, hind leg darker, fore-, midtibia darker apically, hind femur darker subapically, hind tibia darker subbasally; hind tibial comb with six spines; foretarsomere 1 with one basal, one apical spine; midtarsomere 1 with two ventral spines; apical spines of tarsomeres 2–4 of fore-, mid legs 2-2-3, 2-2-3; apical spines of tarsomeres 2–3 of hind leg 1–1, tarsomere 4 absent; fore-, mid-, hind tarsal ratios 2.11 (n = 1), 2.35 (n = 1), 2.42 (n = 1); claws 0.40 (n = 1) length of their respective tarsomere 5. **Abdomen**. Brown. Terminalia (Figure 43c): tergite 9 with truncated apex, apicolateral processes short (not illustrated); sternite 9 with anterior margin not visible in slide mounted specimens, posterior margin with conspicuous convex median lobe bearing two long setae. Gonocoxite brown, stout, 1.70–2.22 (1.95, n = 4) times longer than basal width; gonostylus brown, straight, with broad tip, 0.64–0.75 (0.70, n = 4) length of gonocoxite. Parameres (Figure 43e) 1.0–1.25 (1.11, n = 4) length of aedeagus, generally fused at base for 0.17–0.22 (0.19, n = 2) of total length, separated in some specimens, each with trilobed basal arm; knob flattened; stem sinuous, slightly expanded subapically, tapering apically; distal portion (Figure 43e) bent on two directions, the most basal broad, bent distally to slender pointed tip, 0.33–0.37 (0.35, n = 4) of total length. Aedeagus (Figure 43d) rectangular, heavily sclerotized with two anterolateral curved projections (Figure 43d), two rounded sclerotized areas below basal arch; basal arch sclerotized, concave, extending to 0.25–0.28 (0.26, n = 4) of total length; distal portion with deep mesal notch, forming a bifid apex.

**Female**. Similar to male with usual sexual differences; antenna brown, basal portion of flagellomeres 2–8 slightly pale; antennal ratio 1.03–1.08 (n = 2); palpus with segment 3 with broad, deep sensory pit, palpal ratio 3.00 (n = 1) (Figure 40d); mandible with 10 teeth (n = 1). Wing (Figure 40b) as in male, except for grayish area in M_1_, CuA_1_; length 1.10–1.50 (n = 2) mm; width 0.50–0.70 (n = 2) mm; costal ratio 0.77 (n = 1). Foretarsomere 1 with one basal, one apical spine; midtarsomere 1 with 3–4 ventral spines; apical spines of tarsomeres 2–3 of fore-, mid-, hind legs: 2-2-2; 2-2-2; fore -, mid-, hind tarsal ratios 2.14 (n = 1), 2.36 (n = 1), 2.45 (n = 1); fore-, mid legs claws 0.72 (n = 1) length of their respective tarsomeres 5; hind leg claw 1.27 (n = 1) as long as tarsomere 5 (Figure 40e). Two slightly unequal spermathecae (Figure 40f), measuring 52 by 40 (n = 1) µm and 47 by 35 (n = 1) µm. Third rudimentary spermatheca measuring 7.5 µm (not visible in Figure 40f).

**Specimens examined.** 1 male, pinned, with genitalia in drop of Canada Balsam on celluloid strip on pin, labeled “Allotype *Monohelea stonei* Wirth”, “United States, Louisiana, Baton House, 3 May 1947, Wirth col.” (USNM); 1 female, pinned, labeled “Paratype *Monohelea stonei* Wirth”, same data except “6 May 1947” (FSP); 1 female, pinned, labeled “Paratype *Monohelea stonei* Wirth”, “Florida, Panamá City Beach, Bay County, 6 May 1949, light trap, McElvey col.” (FSP); 1 male, pinned, with genitalia in drop of Canada Balsam on celluloid strip on pin, labeled “Paratype *Monohelea stonei* Wirth”, same data except “Innerarity Point, Escambia County, 4 May 1950, Rathert col.” (FSP); 2 females, pinned, labeled “Paratype *Monohelea stonei* Wirth”, same data except “Santa Rosa Island, 7 July 1949, Butler col.” (FSP); 2 males, pinned, with genitalia in drop of Canada Balsam on celluloid strip on pin, labeled “Paratype *Monohelea stonei* Wirth”, “Virginia, Falls Church, 8 July 1950, Wirth col.” (FSP); 1 male, on microscope slide, same data except “Vero Beach, Indian River, April 1956, light trap” (FSP); 1 female, on microscope slide, same data except “Charlotte County, Englewood beach, 16 June 1960, Jernigan col.” (FSP); 1 female, on microscope slide, labeled “*Monohelea multilineata* (Lutz)” “*Downeshelea stonei* (Wirth)”, “CUBA, Camaguey, La Victoria, 3 July 1957, McGuire col.” (USNM); 1 male, 1 female pinned, with male genitalia in drop of Canada Balsam on celluloid strip on pin, “Panama, Canal Zone, Loma Borracha, 8 July 1952” (FSP).

**Distribution and bionomics.** This species is known from the United States (Iowa, New Jersey, Maryland, Virginia, Tennessee, Mississippi, Georgia, Louisiana and Florida), Bahamas (?), Cuba, Costa Rica (Alajuela) (?) and Panama (Figure 46). It has been found in forested and coastal areas, including seaboard, occurring from sea level to 100 m above sea level.

**Taxonomic discussion**. Wirth [4] described *Downeshelea stonei* based on male and female specimens from the United States, Costa Rica and Panamá. Subsequently, Wirth and Williams [5] recorded this species from the Bahamas and Lane and Wirth [6] examined specimens from Colombia, Cuba, Panama, and Trinidad. The holotype from the United Sates is a female pinned specimen deposited in the USNM (#61094) and could not be studied. Colombia and Costa Rica specimens previously identified as *Downeshelea stonei* show differences in the male genitalia and in some meristic data, indicating that some are *D. alia* sp. nov. The male specimen from Ontario (USA) is misidentified and also corresponds to *D. alia*. Considering the female specimens of both species present faint features to distinguish them, varying in their wide geographical distribution, we recommend the association with males for more confident identification. Specimens from Costa Rica (Alajuela) and the Bahamas were not found for study and due to the lack of further material from the same localities, we maintain the records of *D. stonei* for these countries with a question mark. The discussion about similarities and differences between *D. stonei* and *D. alia* are in the taxonomic discussion of the new species. The male specimen from Trinidad identified by W.W. Wirth [4] had two labels: the first one identifying it as *D. stonei*, the second, with the same handwriting, as *D. guianae*. The specimen is actually *D. guianae*. We presume that Wirth had corrected the misidentification but had not published it.

#### 3.4.35. *Downeshelea tripunctata* sp. nov.

**Figure 41:**
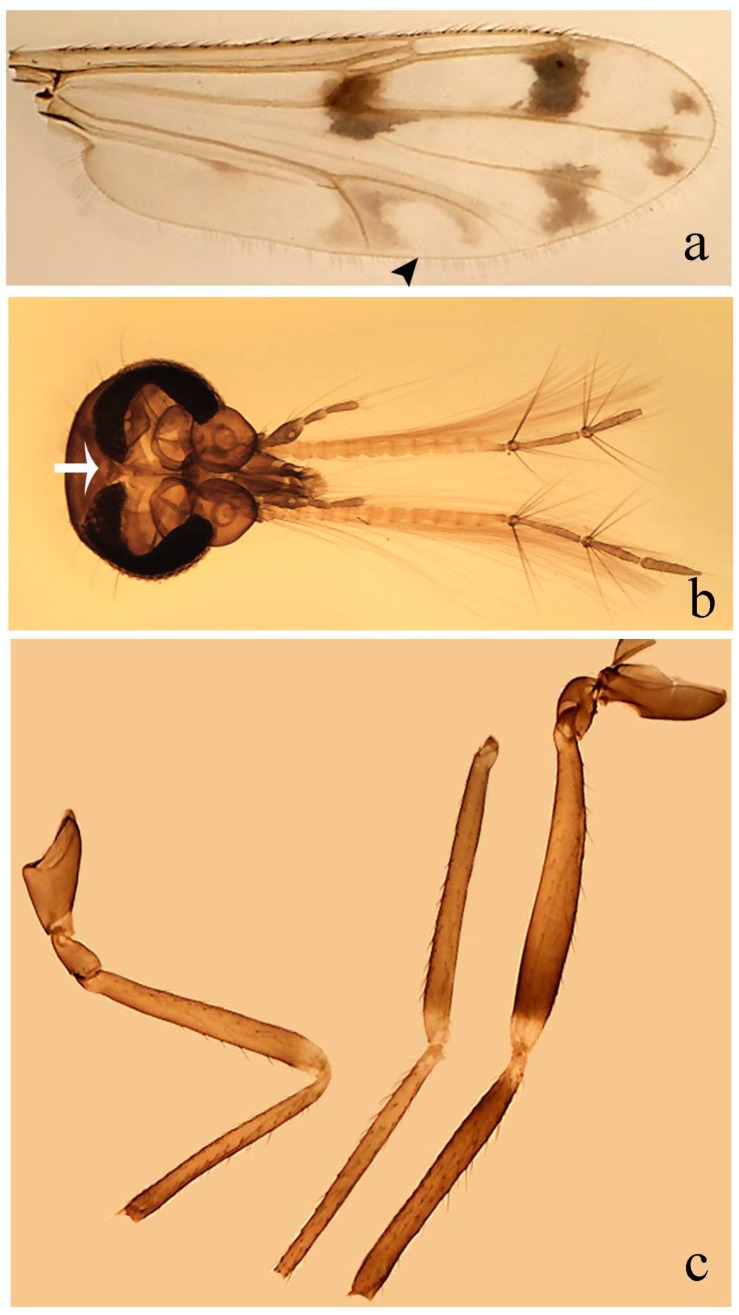
*Downeshelea tripunctata* sp. nov., male: (**a**) wing; arrowhead: cua_1_ margin; (**b**) head, anterior view; arrow: eyes separation; (**c**) fore-, mid-, hind legs (left to right), lateral view.

Figure 41a–c, Figure 43f,g, and Figure 46; Table 1.

**Diagnosis.** Male: only species of *Downeshelea* in the Americas with the following combination of character states: r_3_ with apical grayish spot (Figure 41a); legs brown, hind femur darker subapically (Figure 41c); paramere stem nearly straight (Figure 43g); distal portion short, slender, directed anteriorly, tip pointed bent mesally (Figure 43g); aedeagus rectangular (Figure 43f), with admedian sclerotized process reaching midlength (Figure 43f). Female unknown.

**Description. Male**. **Head** (Figure 41b). Eyes separated dorsomedially by 2.5× width of an ommatidium (Figure 41b). Antenna pale, except basal portion of flagellomere 1, distal portion of flagellomere 10, flagellomeres 11–13 brown; antennal ratio 0.95–0.99 (0.97, n = 2). Palpus with segment 3 swollen on midportion with broad, deep sensory pit; palpal ratio 2.00–2.50 (2.25, n = 2). Thorax. Scutum brown, without definite pattern in slide mounted specimens; pleura pale brown. Wing (Figure 41a) with five grayish areas: three in apical portion of r_3_, m_1,_ M_1,_ not reaching wing margin, spots in m_1,_ M_1_ contiguous in some specimens; one sigmoid-shaped extending from m_1_ to wing margin in m_2_; one over CuA_1,_ CuA_2_ extending into cua_1,_ anal cell, reaching wing margin (Figure 41a); 2nd radial cell twice length of 1st; length 0.97–1.17 (1.07, n = 2) mm; width 0.35–0.40 (0.37, n = 2); costal ratio 0.77–0.81 (0.79, n = 2). Halter brown, distal portion of knob darker. Legs (Figure 41c) brown, hind leg darker, hind femur darker subapically; hind tibial comb with six spines. Foretarsomere 1 with 2 basal, 2 apical spines; midtarsomere 1 with 2–4 ventral spines; apical spines of tarsomeres 2–4 of fore-, mid-, hind legs: 2-2-3(4), 3(4)-2–2, 2-2-2(3); foretarsal ratio 2.43–2.63 (2.53, n = 2); midtarsal ratio 2.44–2.76 2.60, n = 2); hind tarsal ratio 2.08–2.16 (2.12, n = 2); claws 0.38–0.46 (0.42, n = 2) length of their respective tarsomere 5. **Abdomen**. Brown. Terminalia (Figure 43f): tergite 9 with quadrate apex, apicolateral process long, slender; sternite 9 straight anteriorly, posterior margin with large convex median lobe bearing 2–4 long setae. Gonocoxite brown, moderately stout, 2.04–2.33 (2.20, n = 2) times longer than basal width; gonostylus brown, straight, 0.66–0.68 (0.67, n = 2) length of gonocoxite. Parameres (Figure 43g) 0.91–0.97 (0.94, n = 2) length of aedeagus, fused on basal portion for 0.16–0.18 (0.17, n = 2) of total length, each with basal arm trilobed, knob bulbous, anteromedial fusion with moderately deep mesal excavation basally; stem nearly straight; distal portion (Figure 43g) short, slender, bent ventrally, directed anteriorly, tip pointed bent mesally, 0.20–0.34 (0.27, n = 2) of total length. Aedeagus (Figure 43f) rectangular, with admedian sclerotized processes (Figure 43f) reaching midlength of aedeagus, basal arch U-shaped, extending to 0.20 (n = 1) of total length; distal portion with deep mesal notch and two prominent sclerotized pointed processes.

**Female**. Unknown.

**Specimens examined.** Holotype male, on microscope slide, labeled “Holotype *Downeshelea tripunctata* Santarém, Borkent and Felippe-Bauer”, “Costa Rica, Limón, 1 km W. Puerto Viejo, 29 December 1993, A Borkent col.” (CNCI). Paratypes as follows: 1 male, on microscope slide, “Puntarenas, Osa Ciudad, Pto Cortés, Camino Alta Buena Vista, 860 m, 6–8 June 2005, Tp Luz, B Gamboa, J Gutiérrrez, M Moraga, J Azofeifa, Y Cárdenas col.” (MNCR); 1 male, on microscope slide, “ECUADOR, Pastaza Cononaco, 30 May 1976, light trap, J Cohen col.” (USNM).

**Distribution and bionomics**. This species is known from Costa Rica (Puntarenas and Limón) and Ecuador (Figure 46) in coastal and forested areas. In Costa Rica it has been found from the coast up to 860 m above sea level and in Ecuador at 200 m above sea level.

**Etymology**. This species name refers to the presence of three spots in apical portion of the wing.

**Taxonomic discussion.** This species resembles *D. cebacoi*, in having grayish areas in the wing and its general body coloration, but can be distinguished by the male paramere smaller than aedeagus with distal portion short, anteriorly directed with tip bent mesally. *Downeshelea tripunctata* also resembles *D. chirusi* and *D. pulla* in aspects of the male genitalia, but it differ from these species by its brownish body coloration (yellowish in both of the other species), by the legs without dark bands (legs with dark bands in the other species) and by the most dark spots in the wing (paler spots in the wing in the other species).

#### 3.4.36. *Downeshelea venus* sp. nov.

**Figure 42:**
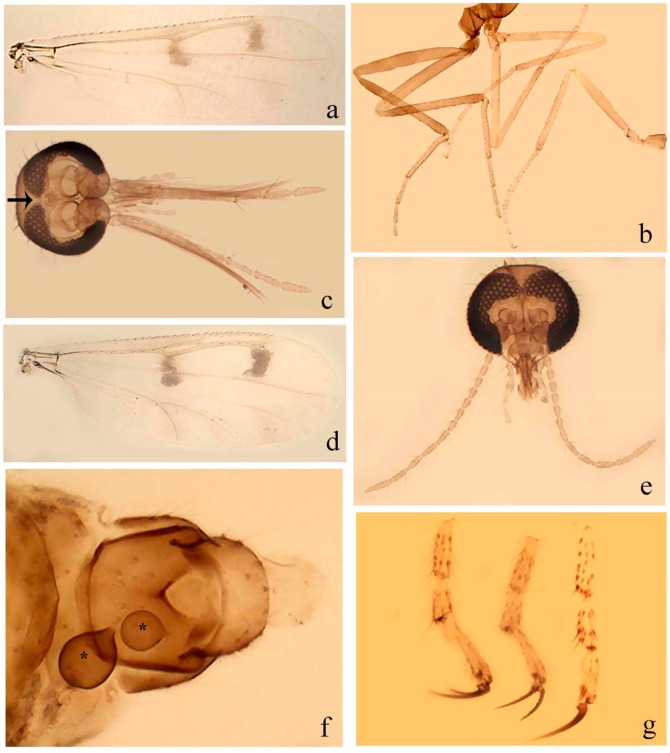
*Downeshelea venus* sp. nov., male: (**a**) wing; (**b**) fore-, mid-, hind legs (left to right), lateral view; (**c**) head, anterior view; arrow: eyes separation. Female: (**d**) wing; (**e**) head, anterior view; (**f**) apex of abdomen, ventral view; asterisks: spermathecae; (**g**) fore-, mid-, hind legs claws (left to right), lateral view.

**Figure 43:**
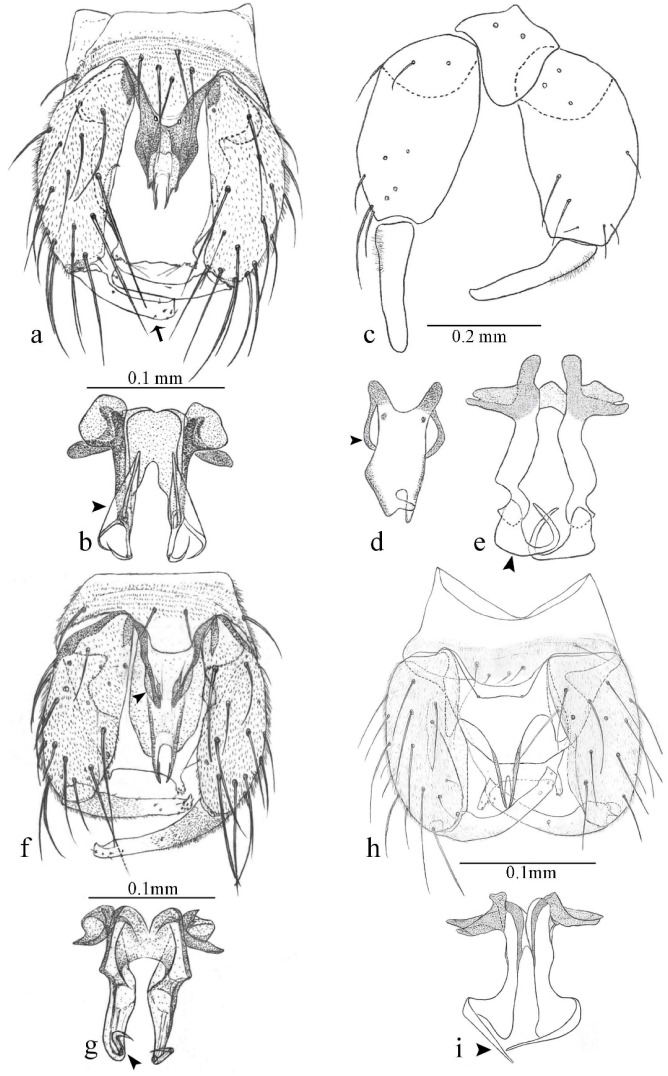
*Downeshelea spatha* sp. nov., male: (**a**) terminalia with aedeagus, ventral view; arrow: gonostylus; (**b**) parameres, ventral view; arrowhead: distal portion. *Downeshelea stonei*, male: (**c**) terminalia, ventral view; (**d**) aedeagus, ventral view; arrowhead: anterolateral projection (**e**) parameres, ventral view; arrowhead: distal portion. *Downeshelea tripunctata* sp. nov., male: (**f**) terminalia with aedeagus, ventral view; arrowhead: aedeagus sclerotized process; (**g**) parameres, ventral view; arrowhead: distal portion. *Downeshelea venus* sp. nov., male: (**h**) terminalia with aedeagus, ventral view; (**i**) parameres, ventral view; arrowhead: distal portion.

Figure 42a–g, Figure 43h,i, and Figure 46; Table 1.

**Diagnosis**. Male: only species of *Downeshelea* in the Americas with the following combination of character states: wing with indistinct grayish spots (Figure 42a); fore, mid legs pale, hind leg pale brown, hind tibia slightly darker subbasally (Figure 42b); foretarsomere 1 without basal spines; distal portion of paramere with broad lobe laterally (Figure 43i); apical portion of lobe with slender projection, posteromesally directed (Figure 43i); aedeagus somewhat Y-shaped, with lateral sclerotized projections (Figure 43h). Female: only species of *Downeshelea* in the Americas with the following combination of character states: wing (Figure 42d) and legs as described for male; hind leg claw 1.00–1.33 as long as tarsomere 5 (Figure 42g); very unequal spermathecae (Figure 42f).

**Description. Male**. **Head** (Figure 42c). Eyes contiguous in lower portion (Figure 42c). Antenna pale except basal portion of flagellomere 1, distal portion of flagellomere 10, flagellomeres 11–13 brown; antennal ratio 0.88–1.05 (0.98, n = 10). Palpus pale brown, with segment 3 slightly swollen on midportion with small, deep sensory pit; palpal ratio 2.50–2.75 (2.57, n = 11). **Thorax**. Scutum yellowish brown, without definite pattern in slide mounted specimens. Wing (Figure 42a) very hyaline, with indistinct grayish spots in M_2_, CuA_1_, CuA_2_; 2nd radial cell 1.8 length of 1st; length 0.85–0.95 (0.91, n = 13) mm; width 0.27–0.35 (0.32, n = 13), costal ratio 0.71–0.76 (0.74, n = 13). Halter pale. Legs (Figure 42b) pale, hind leg pale brown, hind tibia slightly darker subbasally, apically in some specimens; hind tibial comb with seven spines. Foretarsomere 1 with one apical spine, basal spines absent; midtarsomere 1 with 2–5 ventral spines; apical spines of tarsomeres 2–4 of fore-, mid-, hind legs: 2-2-4, 2-2-3, 1-1-2; midtarsomere 1 without basal spines (one spine in some specimens); foretarsal ratio 2.0–2.35 (2.16, n = 13), midtarsal ratio 2.33–2.70 (2.50, n = 13), hind tarsal 1.96–2.20 (2.10, n = 13); claws 0.33–0.45 (0.40, n = 13) length of their respective tarsomere 5. **Abdomen**. Yellowish. Terminalia (Figure 43h): tergite 9 with quadrate apex, apicolateral process long, slender; sternite 9 concave anteriorly, posterior margin with convex median lobe, bearing 3–4 setae. Gonocoxite pale brown, moderately stout, 2.12–2.62 (2.36, n = 12) times longer than basal width; gonostylus pale brown, curved, slender, 0.69–0.78 (0.72, n = 13) length of gonocoxite. Parameres (Figure 43i) 0.89–1.02 (0.97, n = 13) length of aedeagus, fused on basal portion for 0.19–0.26 (n = 13) of total length, each with basal arm trilobed, knob slender; stem slender, straight; distal portion expanded laterodistally, forming broad lobe with apical portion (Figure 43i) bent in a slender projection, with sharp point, posteromesally directed, 0.55–0.70 (0.64) of total length. Aedeagus (Figure 43h) somewhat Y-shaped with large base, basal arch somewhat quadrate, bearing two bristles insertion points, forming lateral sclerotized projections, extending to 0.21–0.38 (0.30, n = 12) of total length; distal portion with moderately deep mesal notch and two sclerotized pointed processes.

**Female**. Similar to male with usual sexual differences; antenna pale; antennal ratio 1.0–1.11 (1.05, n = 12); palpal ratio 1.80–2.25 (2.09, n = 12) (Figure 42e); mandible with 11–13 teeth. Wing as in Figure 42d; length 1.02–1.20 (1.13, n = 12) mm; width 0.42–0.47 (0.45, n = 12) mm; costal ratio 0.75–0.78 (0.76, n = 12). Foretarsomere 1 with one apical spine, basal spines absent (one basal spine in some specimens); midtarsomere 1 with 2–5 ventral spines; apical spines of tarsomeres 2–4 of fore-, mid legs: 2-2-3, 2-2-4; foretarsal ratio 2.11–2.47 (2.26, n = 12), midtarsal ratio 2.39–2.81 (2.56, n = 12), hind tarsal ratio 2.29–2.50 (2.40, n = 12); fore-, mid legs claws 0.69–0.81 (0.76, n = 12) length of their respective tarsomeres 5; hind leg claw 1.00–1.33 (1.22, n = 10) as long as tarsomere 5 (Figure 42g). Two very unequal spermathecae (Figure 42f), the largest ovoid, the smallest rounded, measuring 58–75 (64, n = 12) by 45–55 (51, n = 11) µm and 35–43 (39, n = 12) by 35–40 (37, n = 12) µm. Third rudimentary spermatheca absent.

**Specimens examined.** Holotype male, on microscope slide, labeled “Holotype *Downeshelea venus* Santarém, Borkent and Felippe-Bauer”, “Brazil, Amazonas, Rio Negro, 14 December 1960, light trap, EJ Fittkau col.” (CCER). Allotype female, on microscope slide, labeled “Allotype *Downeshelea venus* Santarém, Borkent and Felippe-Bauer”, same data as holotype except “Rio Tocantins, November 1960” (CCER). Paratypes labeled as follows: 10 males, 17 females, on microscope slide, same data as holotype (4 males, 8 females CCER; 4 males, 7 females USNM; 2 males, 2 females CNCI); 2 males, 9 females, on microscope slide, same data as allotype (1 male, 4 females CCER; 1 male, 5 females USNM).

**Distribution and bionomics.** This species is known only from the Brazilian state of Amazonas (Figure 46). It has been found in riparian environments from 60 to 100 m above sea level.

**Etymology.** This species name refers to Venus, the Roman goddess of love and beauty.

**Taxonomic discussion.***Downeshelea venus* is an unique species in the genus in having a generally very pale yellowish coloration without leg bands, wing with indistinct grayish spots and foretarsomere 1 without basal spines.

#### 3.4.37. *Downeshelea wirthiana* sp. nov.

**Figure 44:**
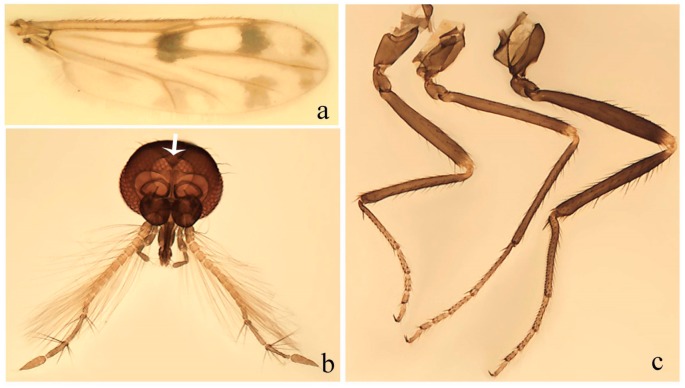
*Downeshelea wirthiana* sp. nov., male: (**a**) wing; (**b**) head, anterior view; arrow: eyes separation; (**c**) fore-, mid-, hind legs (left to right), lateral view.

**Figure 45:**
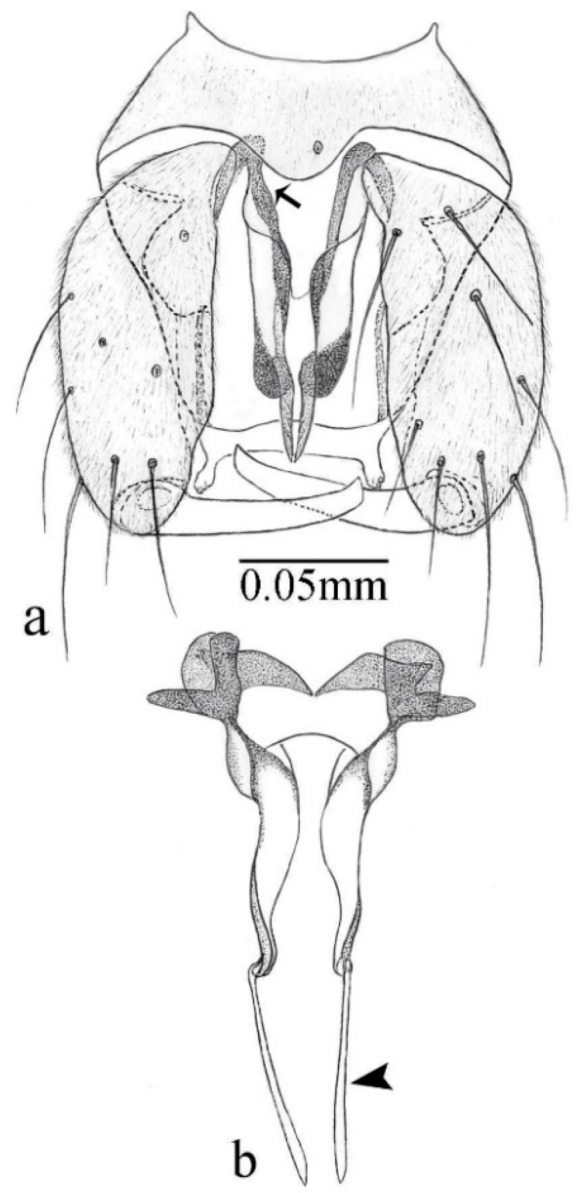
*Downeshelea wirthiana* sp. nov., male: (**a**) terminalia with aedeagus, ventral view; arrow: aedeagus sclerotized lateral projection; (**b**) parameres, ventral view; arrowhead: distal portion.

**Figure 46:**
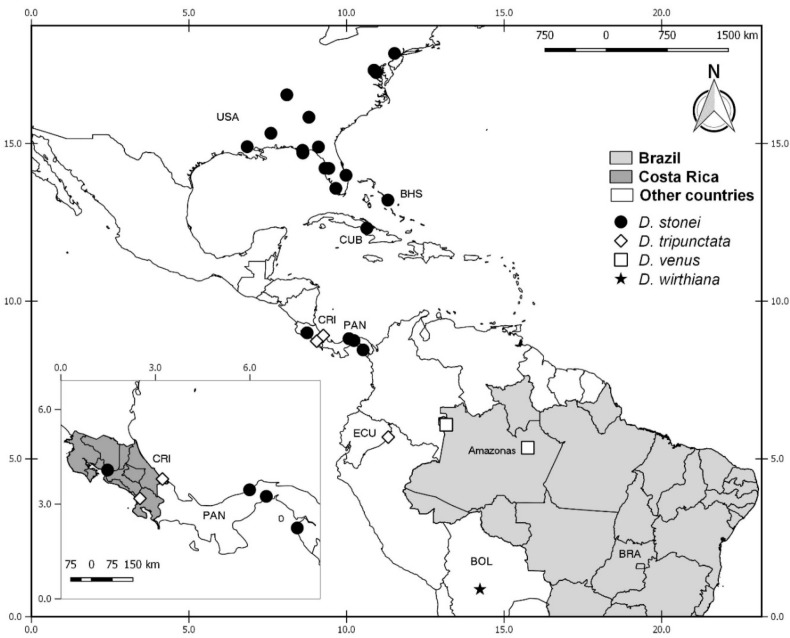
Geographic distribution of *Downeshelea stonei*, *D. tripunctata* sp. nov., *D. venus* sp. nov., and *D. wirthiana* sp. nov.

Figure 44a–c, Figure 45a,b, and Figure 46; Table 1.

**Diagnosis**. Male: only species of *Downeshelea* in the Americas with the following combination of character states: r_3_ with apical grayish spot (Figure 44a); legs brown (Figure 44c); distal portion of paramere very long, slender with flattened point, directed posteriorly (Figure 45b); aedeagus subrectangular (Figure 45a), basal arch with lateral projection heavily sclerotized (Figure 45a). Female unknown.

**Description. Male**. **Head** (Figure 44b). Eyes separated dorsomedially by 2× width of an ommatidium (Figure 44b). Antenna brown; antennal ratio 0.96. Palpus with segment 3 slightly swollen on midportion with broad, deep sensory pit; palpal ratio 2.00. **Thorax**. Scutum brown, postscutellum dark brown; pleura pale brown. Wing (Figure 44a) with four grayish areas: one in apical portion of r_3_, other in M_1_ extending to m_1,_ reaching wing margin; one sigmoid-shaped extending from m_1_ to wing margin in m_2_; one over CuA_1,_ CuA_2_ extending from mediocubital fork into cua_1,_ anal cell, reaching wing margin in CuA_2_; 2nd radial cell twice length of 1st; length 1.02 mm; width 0.35 mm; costal ratio 0.73. Halter brown. Legs (Figure 44c) brown, hind leg darker; hind tibial comb with six spines. Foretarsomere 1 with one basal, one apical spine; midtarsomere 1 without ventral spines; apical spines of tarsomeres 2–4 of fore-, mid-, hind legs: 2-2-3, 2-2-3, 1-1-1; foretarsal ratio 2.21; midtarsal ratio 2.42; hind tarsal ratio 2.32; claws 0.46 length of their respective tarsomere 5. **Abdomen**. Brown. Terminalia (Figure 45a): tergite 9 with quadrate apex, apicolateral process long, slender; sternite 9 concave anteriorly, posterior margin with short convex median lobe bearing one long setae. Gonocoxite brown, slender, 2.62 times longer than basal width; gonostylus brown, nearly straight, 0.62 length of gonocoxite. Parameres (Figure 45b) 1.04 length of aedeagus, fused on basal portion by 0.21 of total length, each with basal arm trilobed, knob bulbous; stem sinuous at midlength, expanded basally, tapering distally; distal portion (Figure 45b) very long, slender, with flattened point, directed posteriorly, 0.65 of total length. Aedeagus (Figure 45a) subrectangular; basal arch shallow, with lateral projection (Figure 45a) heavily sclerotized, extending to 0.09 of total length; distal portion with deep mesal notch, heavily sclerotized laterally, and two prominent sclerotized processes.

**Female**. Unknown.

**Specimens examined.** Holotype male, on microscope slide, labeled “Holotype *Downeshelea wirthiana* Santarém, Borkent and Felippe-Bauer”, “Bolivia, 2 km E Carrasco National Park entrance, 12 km SW Villa Tunari, 517 m (65°29.25′ W; 17°04.28′ S), 16 December 2016, A. Borkent col.” (MNHN).

**Distribution and bionomics.** This species is known only from Bolivia (Figure 46). It was collected in secondary rainforest at 517 m above sea level.

**Etymology.** This species name refers to Dr. Willis Wirth, for his significant contribution to the taxonomic studies of Worldwide Ceratopogonidae fauna.

**Taxonomic discussion.***Downeshelea wirthiana* closely resembles *D. fuscipennis*, *D. kuna* sp. nov. and *D. rodriguezi* sp. nov. in having a wing pattern with grayish spots and a dark brown body. Characters for distinguishing these species are in the discussion section of *D. fuscipennis. D. wirthiana* also resembles *D. cebacoi* in aspects of the male genitalia, but it differs from this species by the dark brown legs and the very long distal portion of the paramere, slender with flattened point.

### 3.5. Unnamed Species of Downeshelea

We studied five female specimens that have wing markings that define the genus. These morphotypes have single features that do not match with any named species of *Downeshelea*. As we have only females which do not differ strongly, we prefer to describe them as two morphotypes and await the collection of males before giving them names.

#### 3.5.1. *Downeshelea* sp. 1

Figure 47a–d.

**Figure 47:**
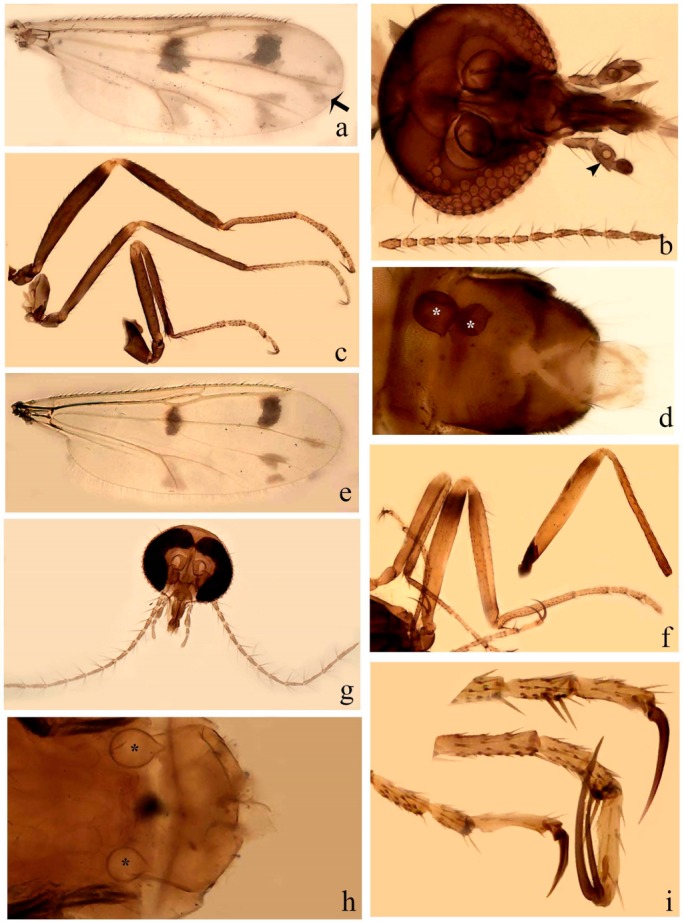
*Downeshelea* sp. 1, female: (**a**) wing; arrow: grayish spot in m_1_ (**b**) head and antenna, anterior view; arrowhead: palpal segment 3; (**c**) fore-, mid-, hind legs (bottom to top), lateral view; (**d**) apex of abdomen, ventral view; asterisks: spermathecae. *Downeshelea* sp. 2, female: (**e**) wing; (**f**) fore-, mid-, hind legs (left to right), lateral view; (**g**) head, anterior view; (**h**) apex of abdomen, ventral view; asterisks: spermathecae; (**i**) fore-, mid-, hind legs claws (bottom to top), lateral view.

**Female.** Antenna (Figure 47b) brown, basal portion of flagellomeres slightly pale, except flagellomere 1; antennal ratio 0.91. Palpus with segment 3 (Figure 47b) short, swollen with broad, deep sensory pit; palpal ratio 1.50; mandible with 9 teeth. Wing (Figure 47a) with four grayish area: first in apical portion of cells r_3_, reaching wing margin; second on M_1_, extending to wing margin in m_1_ (Figure 47a); third sigmoid-shaped extending from median portion of m_1_ to wing margin in m_2_; fourth over CuA_1,_ CuA_2_ extending from mediocubital fork into cua_1,_ anal cell, reaching wing margin in CuA_2_; 2nd radial cell twice length of 1st; length 1.20 mm; width 0.48 mm; costal ratio 0.79. Halter brown. Legs (Figure 47c) brown, hind leg darker; hind tibial comb with seven spines. Foretarsomere 1 with one basal, two apical spines; midtarsomere 1 with 3–4 ventral spines; hind tarsomere 1 with one basal, two apical spines; apical spines of tarsomeres 2–4 of fore-, mid legs: 2-2-4, 2-2-4; foretarsal ratio 2.50; midtarsal ratio 2.50; hind tarsal ratio 2.43; fore-, mid legs claws 0.72 length of their respective tarsomeres 5; hind leg claw 1.27 as long as tarsomere 5. Two unequal spermathecae (Figure 47d) measuring 50 by 48 µm and 38 by 33 µm. Third rudimentary spermatheca measuring 7.5 µm (not visible in Figure 47d).

**Specimens examined**. 1 female on microscope slide labeled “Bolivia, Dept. Santa Cruz, Refugio Los Volcanes, 5.1 km NE Bermejo (63°35.9′ W;18°6.3′ S), 1047 m, 6–28 December 2016, malaise, A. Borkent col.” (MNHN).

**Remarks.** This morphotype is unique in the genus in having a small 3rd palpal segment and the presence of two apical spines in hind tarsomere 1. Also, the presence of grayish spots in apical portion of r_3_ and M1, extending to wing margin in m_1,_ do not match with any known species of the genus. The female was collected from virgin rainforest.

#### 3.5.2. *Downeshelea* sp. 2

Figure 47e–i.

**Female.** Antenna brown; basal portion of flagellomeres slightly pale; antennal ratio 0.92–0.98 (0.95, n = 4). Palpus with segment 3 slightly swollen on midportion with broad, deep sensory pit, palpal ratio 2.60–3.00 (2.79, n = 4) (Figure 47g); mandible with 11–12 teeth. Wing (Figure 47e) with four grayish areas: first near apex of M_1_ not reaching wing margin; second near apex of M_2_; third in m_2_ reaching wing margin; fourth over CuA_2_ reaching wing margin; 2nd radial cell 2.6 length of 1st; length 1.20–1.38 (1.30, n = 4) mm; width 0.48–0.50 (0.49, n = 4) mm; costal ratio 0.80–0.83 (0.81, n = 4). Halter pale brown, distal portion of knob darker. Legs (Figure 47f) yellowish brown, fore-, mid femur darker subapically, fore-, midtibia darker apically; hind femur with subapical, hind tibia with subbasal, apical dark band; hind tibial comb with seven spines. Foretarsomere 1 with one basal, one apical spine; midtarsomere 1 with 5–6 ventral spines; apical spines of tarsomeres 2–4 of fore-, mid legs: 2-2-4, 2-2-2; foretarsal ratio 2.63–2.71 (2.66, n = 4), midtarsal ratio 2.48–2.80 (2.64, n = 4), hind tarsal ratio 2.63–2.81 (2.69, n = 4); foreleg claws unequal, 0.55–0.62 (0.59, n = 4) and 0.70–0.77 (0.73, n = 4) as long as tarsomere 5; mid leg claws greater, unequal, 1.00–1.11 (1.06, n = 4) and 1.20–1.54 (1.42, n = 4) as long as tarsomere 5; hind leg claw 1.43–1.48 (1.45, n = 3) as long as tarsomere 5 (Figure 47i). Two subequal spermathecae (Figure 47h), measuring 53–73 (63, n = 4) by 45–55 (51, n = 4) µm and 50–60 (55, n = 2) by 40–53 (48, n = 4) µm. Third rudimentary spermatheca measuring 10 µm (not visible in Figure 47h).

**Specimens examined**. 1 female, on microscope slide labeled “Colombia, Valle Rio Raposo, February 1964, light trap, V.H. Lee col.” (USNM); 3 females same data except “3–4 June 1964”; “28 July 1964”; October 1964” (USNM).

**Remarks.** The specimens were previously identified as *D. colombiae* by Wirth, based on the wing spots and the leg coloration pattern. However, the presence of unequal claws on the fore- and mid legs, with the foreleg claws smaller than those of the midleg, make this morphotype unique within the genus.

## 4. Conclusions

In this paper, we redescribe *Downeshelea*, providing new and updated features to better characterize the genus, based on Neotropical specimens. Some new records are noted, 10 species are redescribed, and 18 new species are described, increasing the number of species known to 60 worldwide, with 46 of these present in the Neotropical region. Most species are known from a few specimens and only one locality and large areas in the Neotropical Region have never been sampled for *Downeshelea*. This strongly suggests that more species are yet to be discovered and strongly indicates the need for more studies in this region. Species from other biogeographic regions, where few studies have been done and descriptions are dated, are also generally poorly known.

Species of *Downeshelea* are unknown from the Palearctic Region. One species, *D*. *notialisinica* Yu and Hao, is known from China [40] but from the province Guangxi, which belongs to the Oriental Region [41]. The remaining species are restricted to the Afrotropical, Oriental, and Australasian Regions. As such, *Downeshelea* is largely a pantropical group. It seems likely that more species will also be found in the Old World tropics.

Based on distributions, some bionomic features of Neotropical *Downeshelea* species can be provided. They are generally found in humid areas often associated with the rainy season in each locality. Many are found in coastal forested areas, with mangrove, and otherwise are primarily in riparian and humid forested areas. The majority of species are restricted to localities below 500 m in elevation (34 species), with 20 of these recorded only below 100 m in elevation. Nine species have a wider altitudinal distribution, occurring up to 1850 m: *D. cebacoi* (up to 1450 m), *D. chirusi* (up to 1210 m), *D. curta* (from 80 to 745 m), *D. eclectica* (up to 1850 m)*, D. fluminensis* (up to 1100 m), *D. gladius* (up to 860 m), *D. grogani* (up to 1010 m), *D. panamensis* (up to 1250 m) and *D. tripunctata* (up to 860 m). Of these higher altitude species, *D. chirusi*, *D. eclectica*, *D. fluminensis*, *D. grogani*, and *D. panamensis* also have a wide geographical distribution, indicating they can tolerate a greater environmental variation. Only three species—*D. jurgeni*, *D. magna* and *D. moravia*—were found exclusively at high altitudes, from 1600 to 1850 m in Costa Rica, indicating the importance of studying habitats in high altitudes there and elsewhere.

In this paper, we included some important morphological features that can be used to identify some females, but we strongly recommended the association with males, which present more diagnostic characters, primarily in their genitalia. Two female morphotypes are described but remain unnamed, indicating the need of further studies of characters within the genus. This taxonomic study will provide the basis for future phylogenetic approaches to elucidate the probable synapomorphies that determine whether *Downeshelea* is monophyletic and its relationship to other Ceratopogonidae genera. This analysis will contribute to a better understanding of the phylogeny of the Ceratopogonidae family, whose relationships between many genera widely distributed in the Neotropical Region are still unknown. We emphasize, as well, the importance of body and leg coloration for species recognition, being crucial aspects for the separation of some very close and sympatric species (as in *D. chirusi* and *D. pulla*, for example).

Finally, the biology of these species is poorly known and the immature stages of species of *Downeshelea* are unknown. Downes [8] showed that female adults feed on adult Chironomidae, which can also explain the association of *Downeshelea* with aquatic habitats.

## Figures and Tables

**Table 1:** Numerical characters of importance for identification of species of *Downeshelea* for both males and females. Species arranged alphabetically; NA: not available; x: not measurable; n: number of male (♂) and female (♀) specimens measured**.**

Species	Wing Length (mm)		Antennal Ratio		Midtarsomere 1 Ventral Spines		Gonocoxite Length/Width	Gonostylus/Gonocoxite	Paramere/Aedeagus	Female Mandible # Of Teeth	Female Fore-, Mid legs Claw/Tarsomere 5	Female Hind Leg Claw/Tarsomere 5
	Male	Female	Male	Female	Male	Female						
*D. alia* (n = 16 ♂, 11 ♀)	0.95–1.15	0.95–1.15	0.88–1.00	1.00–1.13	0	0	2.03–2.48	0.65–0.78	0.85–1.00	8–10	0.63–0.75	1.16–1.63
*D. avizi* (n = 2 ♂, 4 ♀)	1.35	1.35–1.37	1.13	1.12	3	3–4	2.32–2.40	0.71–0.73	1.35–1.43	11	0.73	1.69
*D. bahiana* (n = 1 ♂)	1.22	NA	0.96	NA	3	NA	2.03	0.71	1.00	NA	NA	NA
*D. balboa* (n = 1 ♂, 2 ♀)	1.11	1.13–1.26	x	1.02	x	x	1.80	0.78	1.05	x	x	x
*D. bicornis* (n = 1 ♂)	1.08	NA	0.86	NA	2–3	NA	1.80	0.74	1.20	NA	NA	NA
*D. bifida* (n = 8 ♂, 3 ♀)	0.92–1.17	1.12–1.27	1.00–1.12	1.03–1.09	0-	0	1.93–2.12	0.73–0.80	1.00–1.06	10	0.68–0.78	1.18–1.43
*D. blantoni* (n = 2 ♂, 2 ♀)	1.08	1.11–1.20	x	x	x	x	1.90–2.00	0.63–0.64	1.06–1.13	x	x	x
*D. capra* (n = 15 ♂, 8 ♀)	0.91–1.15	0.92–0.97	0.91–1.03	1.00–1.06	0	0	2.19–2.46	0.61–0.69	0.93–1.05	8–10	0.56–0.73	1.07–1.23
*D. carioca* (n = 6 ♂, 3 ♀)	1.02–1.37	0.95–1.25	0.93–0.99	x	3–5	3–5	2.15–2.31	0.58–0.63	1.13–1.39	9–11	0.56–0.61	1.17–1.25
*D. casimirensis* (n = 1 ♂)	0.85	NA	0.91	NA	4	NA	2.41	0.81	1.10	NA	NA	NA
*D. castroi* (n = 1 ♂, 1 ♀)	1.47	1.32	1.01	x	4	8	2.17	0.55	1.11	9	0.65	1.31
*D. cebacoi* (n = 6 ♂, 2 ♀)	1.10–1.40	1.30–1.35	1.03–1.10	1.05 -1.09	2–3	5–6	2.40	0.60–0.67	1.27–1.33	12	0.67–0.74	1.32
*D. charrua* (n = 1 ♂, 1 ♀)	1.40	1.56	0.82	0.99	2–4	3–4	2. 40	0.68	0.85	11–12	0.60	1.40
*D. chiapasi* (n = 3 ♂, 2 ♀)	x	1.26–1.45	x	x	x	0	2.00	0.64–0.70	1.15–1.29	10	0.78–0.83	1.35–1.46
*D. chirusi* (n = 17 ♂, 17 ♀)	1.00 -1.27	1.12–1.47	1.00–1.11	1.00–1.11	3–4	3–4	2.27–2.64	0.58–0.70	0.96–1.18	11	0.67–0.80	1.06–1.30
*D. colombiae* (n = 9 ♂, 2 ♀)	1.07–1.25	1.32–1.40	0.99–1.05	1.05–1.09	0–2	1–2	2.03–2.33	0.67–0.75	0.91–0.98	9–10	0.73–0.80	x
*D. costaricensis* (n = 10 ♂, 8 ♀)	0.95–1.10	1.02–1.15	0.90–0.99	0.97–1.12	4–5	4–5	1.71–2.50	0.58–0.66	0.93–1.05	10	0.50–0.75	0.89–1.20
*D. curta* (n = 4 ♂, 4 ♀)	0.85–0.90	0.95–1.15	0.93 -1.02	0.97–1.08	1–2	2–5	1.75–1.96	0.65–0.71	1.22–1.39	7–8	0.56–0.75	1.14–1.31
*D. deanei* (n = 1 ♂, 4 ♀)	0.99	1.07–1.15	x	1.00–1.06	x	3–7	2.20	0.77	1.30	9–10	0.65–0.72	1.11–1.25
*D. divergentis* (n = 1 ♂)	1.15	NA	1.14	NA	0	NA	2.74	0.71	1.13	NA	NA	NA
*D. eclectica* (n = 33 ♂, 33 ♀)	0.92–1.37	1.02–1.30	0.90–1.08	0.97–1.10	7–8	6–9	2.30–2.69	0.55–0.68	0.79–0.98	11–12	0.54–0.81	1.15–1.53
*D. fluminensis* (n = 9 ♂)	1.17–1.30	NA	0.87–0.97	NA	1–2	NA	1.93–2.29	0.57–0.69	1.33–1.69	NA	NA	NA
*D. fuscipennis* (n = 3 ♂, 11 ♀)	0.92–1.00	0.85–1.05	0.94–1.04	0.95–1.10	2	1–3	1.88–2.04	0.72–0.77	1.04–1.26	9–10	0.63–0.77	1.00–1.27
*D. gladius* (n = 5 ♂, 4 ♀)	1.22–1.45	1.20–1.47	0.98–1.03	0.96–1.04	0	0	2.32–2.57	0.54–0.60	1.04–1.18	9	0.74–0.85	1.29–1.45
*D. grogani* (n = 8 ♂)	1.03–1.25	NA	0.92–0.97	NA	1–4	NA	2.17–2.33	0.71–0.78	1.10–1.22	NA	NA	NA
*D. guianae* (n = 5 ♂, 3 ♀)	0.96 -1.00	1.07–1.15	0.85–0.99	1.02–1.10	0	0	2.25–2.50	0.71–0.74	1.07–1.11	9–11	0.65–0.72	1.13–1.33
*D. jarina* (n = 7 ♂, 5 ♀)	0.85–0.95	0.92–1.02	0.88–0.97	1.05–1.10	3–4	3–6	2.04–2.36	0.58–0.64	1.00–1.09	9–10	0.53–0.67	1.17–1.31
*D. jurgeni* (n = 9 ♂, 3 ♀)	1.10–1.22	1.32–1.43	0.93–1.00	1.00–1.04	1 -2	3	1.92–2.17	0.69–0.78	1.05–1.35	11	0.59–0.70	1.07–1.29
*D. kuna* (n = 1 ♂)	0.97	NA	1.10	NA	0	NA	2.13	0.63	1.41	NA	NA	NA
*D. lanei* (n = 2 ♂, 2 ♀)	1.32–1.35	1.35–1.54	0.92	0.97–1.15	1–5	1–3	2.00	0.63	1.40	10–12	0.68–0.73	1.10
*D. litorale* (n = 8 ♂, 5 ♀)	1.00–1.17	1.05–1.25	1.00–1.07	1.06–1.17	2–5	2–5	2.15–2.36	0.58–0.68	1.06–1.23	11–12	0.66–0.87	1.00–1.40
*D. magna* (n = 11 ♀)	NA	1.63–2.00	NA	0.80–0.96	NA	5–7	NA	NA	NA	11–12	0.65–0.77	1.19–1.43
*D. marambaia* (n = 1 ♂)	1.05	NA	1.00	NA	3	NA	1.91–2.17	0.65–0.66	1.16–1.17	NA	NA	NA
*D. moravia* (n = 1 ♂, 1 ♀)	1.37	1.57	1.02	x	0	0	2.30	0.69	1.02	11	0.64	1.29
*D. multilineata* (n = 11 ♂, 5 ♀)	0.97–1.15	1.12–1.17	0.91–1.06	1.02	2 – 5	5–6	1.90–2.40	0.58–0.67	0.96–1.21	11	0.61–0.72	1.18–1.22
*D. oliveirai* (n = 5 ♂, 10 ♀)	1.13–1.32	1.08–1.46	0.94–0.99	1.08–1.17	3 – 4	3–4	1.92–2.22	0.60–0.72	0.85–0.97	11	0.60–0.76	1.06–1.18
*D. panamensis* (n = 14 ♂, 8 ♀)	0.90–1.15	0.97–1.17	0.90–1.00	1.12–1.19	2 – 3	2–5	2.12–2.42	0.60–0.67	1.13–1.31	8–10	0.56–0.68	1.07–1.25
*D. pulla* (n = 13 ♂, 10 ♀)	1.02–1.22	1.15–1.40	0.93–0.99	1.05–1.16	2 – 3	2–4	2.67–3.15	0.53–0.69	1.02–1.24	10–11	0.70–0.79	1.33–1.44
*D. quasidentica* (n = 2 ♂)	1.05–1.15	NA	x	NA	1 – 2	NA	2.30	0.72	1.60	NA	NA	NA
*D. quechua* (n = 1 ♂)	1.10	NA	0.97	NA	0	NA	2.10	0.75	1.02	NA	NA	NA
*D. rodriguezi* (n = 2 ♂, 2 ♀)	0.90–0.97	1.00	0.87–0.89	1.02	1 – 2	2–3	2.10–2.21	0.69–0.74	1.11–1.13	9	0.64–0.66	1.08–1.27
*D. spatha* (n = 5 ♂, 7 ♀)	1.05–1.22	1.12–1.27	0.98–1.07	1.08–1.20	1- 2	2–4	2.61–2.78	0.56–0.67	1.04–1.13	9–11	0.61–0.75	1.33–1.50
*D. stonei* (n = 4 ♂, 2 ♀)	1.38	1.10–1.50	1.02	1.03–1.08	2	3–4	1.70–2.22	0.64–0.75	1.00–1.25	10	0.72	1.27
*D. tripunctata* (n = 3 ♂)	0.97–1.17	NA	0.95–0.99	NA	2–4	NA	2.04–2.33	0.66–0.68	0.91–0.97	NA	NA	NA
*D. venus* (n = 13 ♂, 12 ♀)	0.85–0.95	1.02–1.20	0.88–1.05	1.00–1.11	2–5	2–5	2.12–2.62	0.69–0.78	0.89–1.02	11–13	0.69–0.81	1.00–1.33
*D. wirthiana* (n = 1 ♂)	1.02	NA	0.96	NA	0	NA	2.62	0.62	1.04	NA	NA	NA

## References

[1] Borkent A., Dominiak P. Catalog of the Biting Midges of the World (Diptera: Ceratopogonidae). Zootaxa.

[2] Santarém M.C.A., Felippe-Bauer M.L. Brazilian Species of Biting Midges. https://portal.fiocruz.br/sites/portal.fiocruz.br/files/documentos/brazilian_species_of_biting_midges_2019.pdf.

[3] Wirth W.W., Departamento de Zoologia de São Paulo (1974). Chapter 14. Family Ceratopogonidae. A Catalog of the Diptera of the Americas South of the United States.

[4] Wirth W.W. (1953). American biting midges of the heleid genus *Monohelea*. Proc. US Natl. Mus..

[5] Wirth W.W., Williams R.W. (1964). New species and records of North American *Monohelea* (Diptera: Ceratopogonidae). Ann. Entomol. Soc. Am..

[6] Lane J., Wirth W.W. (1964). The biting midge genus *Monohelea* Kieffer in the Neotropical Region (Diptera, Ceratopogonidae). Stud. Entomol..

[7] Wirth W.W., Grogan W.L. (1988). The Predaceous Midges of the World (Diptera: Ceratopogonidae; Tribe Ceratopogonini).

[8] Downes J. (1978). Feeding and mating in the insectivorous Ceratopogoninae (Diptera). Mem. Entomol. Soc. Can..

[9] Yasumatsu K., Wongsiri T., Tirawat C., Wongsiri N., Lewvanich A. (1981). Contributions to the Development of Integrated Rice Pest Control in Thailand. Dept. Agr. Min. Agr. Coop. Gov. Thail. Jpn. Int. Coop. Agency.

[10] Santarém M.C.A., Borkent A., Spinelli G.R., Felippe-Bauer M.L. (2018). New Neotropical species of *Downeshelea* Wirth and Grogan and redescription of *D. multilineata* (Lutz) (Diptera: Ceratopogonidae). J. Nat. Hist..

[11] Ratanaworabhan N.C., Wirth W.W. (1972). The biting midge genus *Monohelea* Kieffer in the Oriental Region. Pac. Insects.

[12] Debenham M. (1972). Australian and New Guinea ‘picture-wing’ species of the genus *Monohelea* Kieffer (Diptera: Ceratopogonidae). Aust. J. Zool. Suppl. Ser..

[13] Clastrier J., Delécolle J.C. (1990). Description d’un nouveau genre et de nouvelles espéces africaines des genres *Allohelea* Kieffer, *Monohelea* Kieffer, *Downeshelea* Wirth & Grogan, *Boreohelea* nov. gen. (Diptera: Ceratopogonidae). Ann. Soc. Entomol. Fr..

[14] Borkent A., Spinelli G.R., Grogan W.L., Brown B.V., Borkent A., Cumming J.M., Wood D.M., Woodley N.E., Zumbado M.A. (2009). Chapter 29: Ceratopogonidae (biting midges, purrujas). Manual of Central America Diptera.

[15] Felippe-Bauer M.L., Quintelas A.R. (1993). *Downeshelea bicornis*, a new Neotropical predaceous midge from Brazil (Diptera: Ceratopogonidae). Mem. Inst. Oswaldo Cruz..

[16] Felippe-Bauer M.L., Quintelas A.R. (1993). Two new Brazilian predaceous midges of the genus *Downeshelea* Wirth and Grogan (Diptera: Ceratopogonidae). Mem. Inst. Oswaldo Cruz..

[17] Felippe-Bauer M.L., Spinelli G.R. (1994). Two new Neotropical species of *Monohelea* Kieffer and *Downeshelea* Wirth and Grogan (Diptera: Ceratopogonidae). Mem. Inst. Oswaldo Cruz..

[18] Felippe-Bauer M.L., Quintelas A.R., Spinelli G.R. (1995). A new Neotropical predaceous midge, *Downeshelea deanei*, and redescription of *Downeshelea guianae* (Wirth) (Diptera: Ceratopogonidae). Mem. Inst. Oswaldo Cruz..

[19] Felippe-Bauer M.L., Silva C.S. (2008). *Downeshelea oliveirai*, a new Neotropical predaceous midge from northern Brazil (Diptera, Ceratopogonidae). Iheringia Sér. Zool..

[20] Felippe-Bauer M.L., Silva T.N., Ribeiro E.S., Borkent A. (2011). A new species of *Downeshelea* Wirth & Grogan and a redescription of the male of *Downeshelea cebacoi* (Lane & Wirth) (Diptera: Ceratopogonidae). Zootaxa.

[21] Huerta H., Felippe-Bauer M.L., Spinelli G.R. (2012). A new species and new records of *Downeshelea* Wirth & Grogan in Neotropical Mexico (Diptera: Ceratopogonidae). Zootaxa.

[22] Borkent A. World Species of Biting Midges (Diptera: Ceratopogonidae). http://wwx.inhs.illinois.edu/files/4514/6410/0252/CeratopogonidaeCatalog.pdf.

[23] Borkent A. (2014). The pupae of the Biting Midges of the world (Diptera: Ceratopogonidae), with a generic key and analysis of the phylogenetic relationships between genera. Zootaxa.

[24] Borkent A., Wirth W.W. (1997). World species of biting midges (Diptera: Ceratopogonidae). Bull. Am. Mus. Nat. Hist..

[25] Borkent A., Spinelli G.R. (2000). Catalog of New World biting midges south of the United States (Diptera: Ceratopogonidae). Contrib. Entomol. Int..

[26] Borkent A., Spinelli G.R., Adis J., Arias J.R., Rueda-Delgado G., Wantzen K.M. (2007). Neotropical Ceratopogonidae (Diptera: Insecta). Aquatic Biodiversity in Latin America (ABLA).

[27] Huerta H., Ibáñez-Bernal S., Felippe-Bauer M.L. (1999). New records of biting midges (Diptera: Ceratopogonidae) in Mexico. Entomol. Y Vectores.

[28] Lutz A. (1914). Contribuição para o conhecimento das ceratopogoninas do Brazil. Mem. Inst. Oswaldo Cruz..

[29] Macfie J.W.S. (1940). Ceratopogonidae (Diptera) from British Guiana and Trinidad. Part 2. Proc. R Entomol. Soc. Lond. Ser. B Taxon.

[30] Johannsen O.A.A. (1943). Generic synopsis of the Ceratopogonidae (Heleidae) of the Americas, a bibliography, and a list of the North American species. Ann. Entomol. Soc. Am..

[31] Lane J. (1945). Redescrição de ceratopogonídeos neotrópicos (Diptera: Ceratopogonidae). Rev. Entomol..

[32] Wilkening A.J., Kline D.L., Wirth W.W. (1985). An annotated checklist of the Ceratopogonidae (Diptera) of Florida with a new synonymy. Fla. Entomol..

[33] Borkent A., Grogan W.L. (2009). Catalog of the new world biting midges north of Mexico (Diptera: Ceratopogonidae). Zootaxa.

[34] Grogan W.L., Hribar L.J., Murphree C.S., Cilek J.E. (2010). New records of biting and predaceous midges from Florida, including species new to the fauna of the United States (Diptera: Ceratopogonidae). Insecta Mundi.

[35] Tavares O., Silva-Pereira A.J. (1978). Duas espécies novas do gênero *Monohelea* Kieffer, 1917, do Estado do Rio de Janeiro, Brasil (Diptera, Ceratopogonidae). Rev. Bras. Biol..

[36] Borkent A., Brown B.V., Adler P.H., Amorim D.D.S., Barber K., Bickel D., Boucher S., Brooks S.E., Burger J., Burington Z.L. (2018). Remarkable fly (Diptera) diversity in a patch of Costa Rican cloud forest: Why inventory is a vital science. Zootaxa.

[37] Brown B.V., Borkent A., Adler P.H., Amorim D.D.S., Barber K., Bickel D., Boucher S., Brooks S.E., Burger J., Burington Z.L. (2018). Comprehensive inventory of true flies (Diptera) at a tropical site. Commun. Biol..

[38] Wirth W.W., Grogan W.L. (1981). Natural History of Plummers Island, Maryland. XXV. Biting midges (Diptera: Ceratopogonidae). 3. The species of the tribe Stilobezziini. Bull. Biol. Soc. Wash..

[39] McKeever S., Hagan D.V., Grogan W.L. (1991). Comparative study of mouthparts of 10 species of predaceous midges of the tribe Ceratopogonini (Diptera, Ceratopogonidae). Ann. Entomol. Soc. Am..

[40] Yu Y.X., Liu J.H., Liu G.P., Liu Z.J., Hao B.S., Yan G., Zhao T.S. (2005). Ceratopogonidae of China, Insecta, Diptera.

[41] Szadziewski R., Borkent A., Dominiak P. Ceratopogonidae. Fauna Europaea. www.faunaeur.org.

